# Prevalence of osteoporosis and associated factors among Chinese adults: a systematic review and modelling study

**DOI:** 10.7189/jogh.15.04009

**Published:** 2025-01-17

**Authors:** Yi Liu, Xuanyin Huang, Ke Tang, Jing Wu, Jiali Zhou, He Bai, Liying Zhou, Shiyi Shan, Zeyu Luo, Jin Cao, Peige Song, Igor Rudan

**Affiliations:** 1 Centre for Clinical Big Data and Statistics of the Second Affiliated Hospital, Zhejiang University School of Medicine, School of Public Health, Zhejiang University School of Medicine, Hangzhou, China; 2 Centre for Global Health, Usher Institute, University of Edinburgh, Edinburgh, Scotland, UK

## Abstract

**Background:**

Osteoporosis is a degenerative disease of bone metabolism. The epidemiology of osteoporosis varies by age, sex, and geography. There is a lack of information on the prevalence of osteoporosis among Chinese adults. In this study, we aimed to estimate the prevalence of osteoporosis among Chinese adults by age, sex, and skeletal sites and explore the main associated factors.

**Methods:**

We searched six bibliographic databases to identify epidemiological studies that reported the prevalence of osteoporosis among Chinese adults published between January 1990 and February 2022. We applied a multilevel mixed-effects meta-regression to estimate the age-specific prevalence of osteoporosis. We presented the age-specific prevalence of osteoporosis by sex, diagnostic criteria (World Health Organization (WHO) and Chinese (CHN) diagnostic criteria), and specific skeletal site (the lumbar spine, femoral neck, and ward’s triangle). We used the population data from the seventh National Census of Mainland China to estimate the number of Chinese adults with osteoporosis in 2020. Major associated factors for osteoporosis were pooled by a random-effects meta-analysis. We also estimated the regional prevalence and cases of osteoporosis among 31 provinces in mainland China in 2020 using an ‘associated factor-based model.’

**Results:**

We included 129 articles in this systematic review and modelling study. 32 were based on the WHO diagnostic criteria and 17 on the CHN diagnostic criteria. Additionally, we included 83 articles in the associated factor analysis. The prevalence of osteoporosis increased with age and was consistently higher in females than males, regardless of diagnostic criteria and skeletal sites. Whether based on the WHO criteria (13.54%, 95% confidence interval (CI) = 10.25, 18.11) or the CHN criteria (29.49%, 95% CI = 18.29, 43.5), the prevalence of osteoporosis among Chinese adults aged 20–89 years in 2020 was highest when measuring the ward’s triangle, which translated to 145.86 million (95% CI = 110.41, 195.03) and 317.54 million (95% CI = 196.95, 468.47) affected adults, respectively. The prevalence of osteoporosis was the highest in Northeast China under both the WHO criteria (15.50%, 95% CI = 11.78, 20.59) and the CHN criteria (32.36%, 95% CI = 20.33, 46.8), with the ward being the measured skeletal site. Marital status, current smoking, underweight, hypertension, fracture history, longer menopause years and menopause were positively associated with osteoporosis.

**Conclusions:**

Osteoporosis remains a significant public health concern in China, particularly among females and the elderly. Meanwhile, the prevalence of osteoporosis varies considerably by region, skeletal site and diagnostic criteria. It is essential to establish clear and unified diagnostic criteria and implement high-quality epidemiological studies for osteoporosis in China. Additionally, targeted preventive strategies that focus on individuals with characteristics associated with osteoporosis are required to mitigate the impact of this condition.

**Registration:**

PROSPERO: CRD42024564441.

Osteoporosis is a systemic skeletal disorder characterised by decreased bone density and microstructural deterioration. It leads to increased bone fragility and susceptibility to fractures [1]. This ‘silent’ disease significantly increases the risk of fractures, scoliosis, and pain, contributing to increased morbidity, mortality, and economic burden on health care systems [2]. According to the World Health Organization (WHO), osteoporosis is defined based on a T-score <2.5 standard deviations (SD) at the lumbar spine, hip, or mid-radius as measured by dual-energy x-ray absorptiometry (DXA) [1]. Utilising this criterion, the prevalence of osteoporosis has been estimated at 19.7%, with the prevalence increasing with advanced age [3]. As the global population continues to age, the burden of osteoporosis is also on the rise, making it a significant public health concern.

However, the assessment of osteoporosis prevalence is challenged by inconsistent diagnostic criteria and measurement methodologies among studies. For example, selecting either lateral or anteroposterior spine DXA measurements can lead to significant differences in osteoporosis classification. Similarly, comparisons between the ward’s triangle and other skeletal sites can also lead to notable disparities in osteoporosis classification [4]. These variations highlight the importance of carefully considering measurement techniques and skeletal sites when assessing osteoporosis. Additionally, when comparing the prevalence of osteoporosis between studies, it is crucial to account for the differences in assessment methods employed in each study.

The aetiology of osteoporosis is multifactorial, involving a complex interplay of genetic, comorbidity, environmental, and lifestyle factors [5–13]. Epidemiological investigations have identified several major associated factors, including age, sex, body mass index, physical inactivity, smoking, excessive alcohol consumption, and dietary factors such as low calcium and vitamin D intake [10,12,14–16]. Notably, age and sex are two of the most significant factors, with the prevalence of osteoporosis increasing with advanced age and being much higher in women, particularly after menopause [3,14,17–20]. Understanding these factors is essential to developing effective prevention and management strategies for osteoporosis.

In China, the prevalence of osteoporosis is a growing public health issue, paralleled by the country’s rapid demographic transition and the ageing of its population [21]. For reasons such as lower peak bone mass in Asian populations compared to white populations, China recommends using a T-score <2.0 SD, or 25% reduction in bone mineral density (BMD), for osteoporosis diagnosis [22]. However, current studies conducted in Chinese populations display considerable heterogeneity in diagnostic criteria, WHO or Chinese (CHN), and measurement of skeletal sites, with significant variations in the reported prevalence of osteoporosis [23]. These discrepancies present considerable challenges for assessing the national epidemiology of osteoporosis. A previous meta-analysis in China, which included 69 articles, pooled osteoporosis prevalence between 2003–15 among the Chinese population. However, the study included a mixture of different diagnostic criteria and skeletal sites and did not explore the main factors associated with osteoporosis [23]. To fill this knowledge gap, we set out to determine the prevalence of osteoporosis in the general Chinese adult population by widely adopted criteria (WHO and CHN) and skeletal sites, as well as its variation by age, sex and region. We also investigated the major factors associated with osteoporosis.

## METHODS

This systematic review and modelling study was registered on PROSPERO (registration number CRD42024564441). We followed the Preferred Reporting Items for Systematic Reviews and Meta-Analyses (PRISMA) guidelines and the Guidelines for Accurate and Transparent Health Estimates Reporting (GATHER) statement [24,25].

### Search strategy and selection criteria

We searched three Chinese and three English bibliographic databases, namely China National Knowledge Infrastructure, Wangfang and CQVIP, PubMed, EMBASE and Medline, to identify all epidemiologic studies on osteoporosis prevalence among Chinese people. We searched for studies that were published between January 1990 and February 2022. We did not apply any language restrictions to the search process. We developed specific comprehensive search strategies combined with the terms ‘osteoporosis’ (osteopenia or osteoporosis or OP or bone density or bone mineral density or BMD or bone loss), ‘prevalence’ (inciden* or prevalen* or epidemiolog*) and ‘China’ (China or Chinese, only in English databases) (Table S1 in the **Online Supplementary Document**). Moreover, we examined the reference lists of all included studies and related reviews to supplement the database searches.

Four authors (YL, XH, KT, and HB) independently conducted the title and abstract screening and full-text review. Conflicts were resolved through a discussion with a senior researcher (PS). To better represent the population of Chinese adults, we included only studies conducted among the general population in China that reported osteoporosis prevalence based on cross-sectional investigations or cross-sectional analysis of cohorts. In order to reduce bias to the maximum extent, we excluded studies that did not include clear assessment methods of osteoporosis or that relied only on self-reported diagnosis. We excluded studies conducted among specific populations (*e.g.* based on increased body mass index (BMI) status or conducted in children only) as they were not representative of the general population. Additionally, we excluded studies that were published in the form of abstracts, letters, reviews, viewpoints, or case reports. Wherever several studies were based on an overlapping study population, we included only the most representative study (*i.e.* either the one that was most recent, had the largest sample, or had the most comprehensive description).

### Data extraction and quality assessment

Data were independently extracted by the same co-authors of this study (YL, XH, KT, and HB). Discrepancies were resolved by their consensus or by following the discussions with PS. We developed and used a pilot-tested and refined extraction form to extract the following data from all included articles:

1. Study characteristics: author (s), publication year, study setting (urban, rural or mixed), study location (province, city, latitude, and longitude), investigation year, sampling method;
2. Sample characteristics: the proportion of female participants and age (age range, mean or median age, or midpoint of the age range);
3. Osteoporosis assessment: diagnostic criteria, skeletal site (s), measurement method;
4. Prevalence estimates: sample size, number of osteoporosis cases, and prevalence by age group, sex, region, diagnostic criteria, and skeletal site, where available.

For articles with censored age group data, we assumed that the age intervals for these groups were the same as those reported for the other age groups within the same articles. For the included studies that evaluated the associated factors using multivariable logistic regressions, we additionally extracted the definitions of associated factors, reference groups, odds ratios (ORs), and 95% confidence intervals (CIs).

Three researchers (YL, XH, and KT) independently assessed the quality of all included articles according to five domains – sample population, sample size, participation rate, outcome assessment, and analytical methods (Table S2 in the **Online Supplementary Document**) [26]. The maximum score of ten represents the overall quality, with each domain ranging from zero to two. Higher total scores indicated better quality of study methodology.

### Statistical analysis

#### Epidemiological modelling of the osteoporosis prevalence

To reduce heterogeneity, we only included studies that defined osteoporosis using the diagnostic criteria of the WHO (T-score≤–2.5 SD) or CHN (T-score≤–2 SD, or 25% reduction of BMD) and categorised them accordingly. In addition, the included studies assessed osteoporosis at various skeletal sites. However, we only chose those that assessed the following specific sites: lumbar spine, femoral neck, or ward’s triangle.

Given the well-acknowledged high discrepancy in prevalence between males and females, we constructed the prevalence estimation models separately by sex. First, we used a univariable meta-regression to explore the association of osteoporosis prevalence and each cluster-level factor, including age, publication year, investigation year, study setting, and latitude (Table S3 in the **Online Supplementary Document**). Considering the limited number of data points included in each model, age was the only factor included in the final multivariable models. To control the effects of multiple data points from the same study, we added a random effect (u_i) into the model. Finally, we estimated the age-specific prevalence of osteoporosis by diagnostic criteria and by skeletal site for males and females, respectively.

#### Estimation of the national prevalence and cases of osteoporosis in 2020

We calculated the number of Chinese adults with osteoporosis by diagnostic criteria and skeletal site in males and females by multiplying the age-specific osteoporosis prevalence estimates with the corresponding population size obtained from the seventh National Census of Mainland China [27]. This was done for every five-year age group, ranging from 20–89 years. Then, we derived the overall prevalence and cases of osteoporosis among Chinese adults in the year 2020 from those age-specific estimates.

#### Meta-analysis of factors associated with osteoporosis

We used a random-effects meta-analysis to pool the ORs for major associated factors of osteoporosis. Due to limited information on associated factors in the included studies, we included all reported associated factors across these studies, regardless of the diagnostic criteria and skeletal sites used. As a rule, we only included factors that had been investigated in at least three individual studies using a multivariate logistic regression. To ensure consistency among the associated factors across all included studies, we converted them to a unified unit based on the most commonly used unit in the included studies. We assessed the heterogeneity between studies using Cochran’s Q statistic (*P*-value <0.05 indicates significant heterogeneity) and the *I^2^* statistic (≥50% indicates substantial heterogeneity) [28,29]. Two researchers (XH and KT) assessed the evidence credibility of associated factors and divided them into five categories: class I (convincing evidence), class II (highly suggestive evidence), class III (suggestive evidence), class IV (weak evidence), and NS (non-significant) (Table S4 in the **Online Supplementary Document**).

#### Estimation of the regional prevalence and cases of osteoporosis in 2020

We also estimated the regional prevalence and cases of osteoporosis in 2020 using an ‘associated factor-based model.’ To examine the distribution of osteoporosis in different regions, we classified China into six geographic regions (Table S5 in the **Online Supplementary Document**). Four major associated factors (overweight, obesity, hypertension, and current smoking) were found to have a significant association with the prevalence of osteoporosis in the previous step and have subnational prevalence data [30–32]. Therefore, we included them in the ‘associated factor-based model.’ Using the same ‘associated factor-based’ approach, we also estimated the prevalence and the number of cases of osteoporosis among 31 provinces in mainland China in 2020.

We conducted all analyses using *R*, version 4.3.2 (R Core Team, Vienna, Austria). We draw China map using ArcMap, version 10.8 (Environmental Systems Research Institute, Redlands, CA, USA). Statistical tests were two-sided, and a *P* < 0.05 indicated statistical significance.

## RESULTS

### Study selection and characteristics

**Figure 1:**
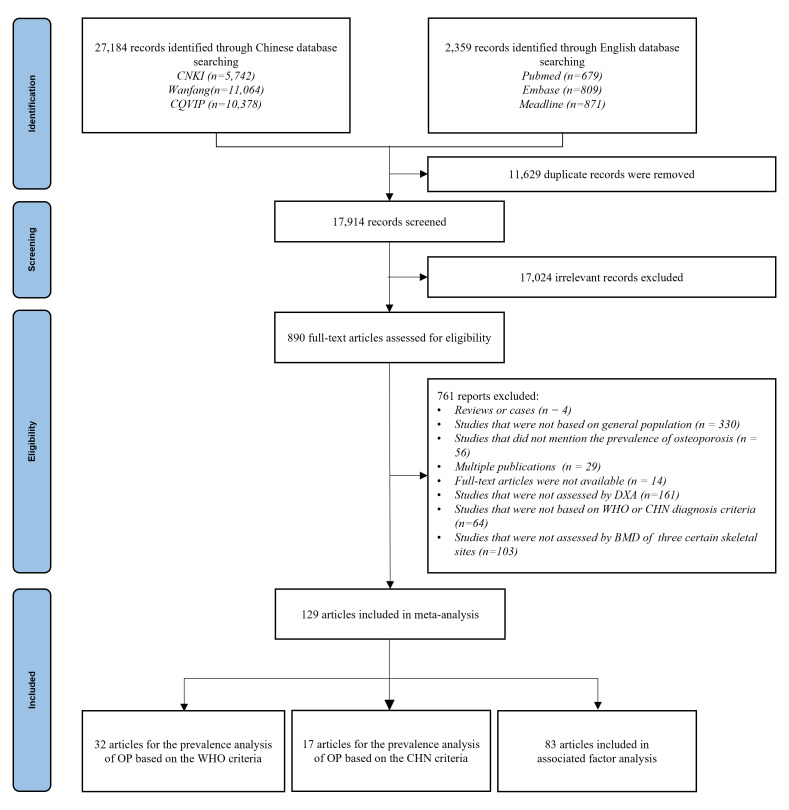
Study selection and flowchart.

**Figure 2:**
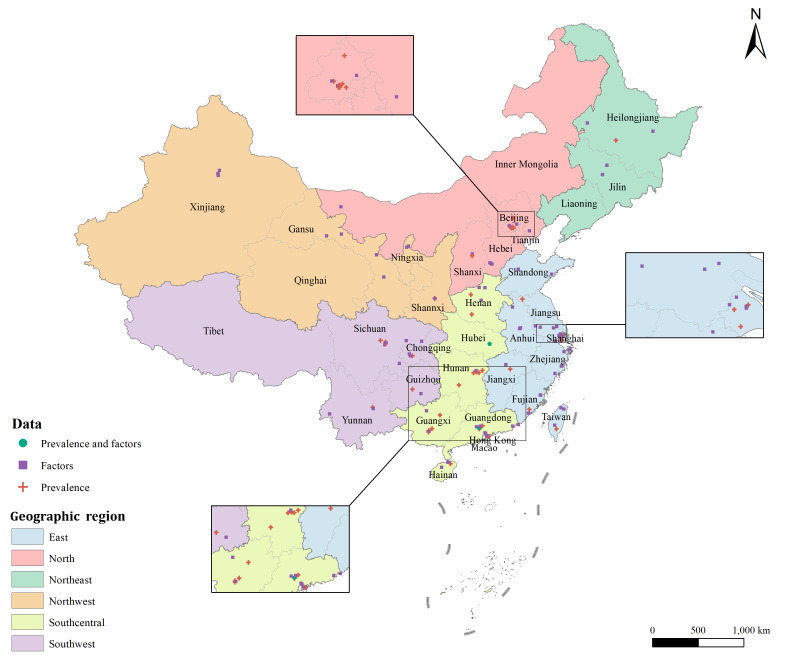
Geographical distribution of articles included in this study (n = 129).

We identified a total of 27 184 records from the Chinese database and 2359 records from the English database. After removing duplicates and an initial screening of titles and abstracts, we assessed the full text of 890 articles. Finally, we included 129 articles in our systematic review and analysis, among which 32 were included in the prevalence analysis based on the WHO diagnosis criteria, 17 articles in the prevalence analysis based on the CHN diagnosis criteria, and 83 articles in the associated factor analysis (**Figure 1**). Articles included in our study covered 27 provinces presented in the distribution map (**Figure 2**). The detailed characteristics and quality appraisal of all included articles are illustrated in Tables S6–8 in the **Online Supplementary Document**.

### National prevalence and cases of osteoporosis in China in 2020

**Figure 3:**
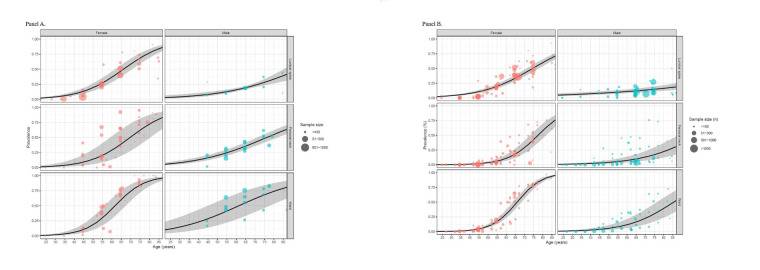
Estimated prevalence of osteoporosis by age, sex, and skeletal site in China in 2020.

In general, the prevalence of different skeletal sites in different genders increased with age, and osteoporosis in females was higher overall than in males. For the groups aged >55 years, females were at a higher risk of osteoporosis than males, regardless of the skeletal site that was measured (**Figure 3**).

Under the WHO criteria, measuring osteoporosis at the ward site showed the highest prevalence in China at 13.54% (95% CI = 10.25, 18.11). Among females, the prevalence was also highest when measured at the ward, reaching 19.26%, increasing from 0.16% in the 20–24 age group (95% CI = 0.1, 0.24) to 95.65% in the 85–90 age group (95% CI = 93.74, 97.0). In males, however, the highest rate was at the lumbar spine (8.63%; 95% CI = 6.10, 12.12), rising from 4.75% in the 20–24 age group (95% CI = 2.9, 7.71) to 18.65 in the 85–90 age group (95% CI = 13.58, 25.07). When applying these estimates to the 2020 Chinese population size, there were 145.86 million cases of osteoporosis in China when measured at the ward site (95% CI = 110.41, 195.03). Among females, the number of cases was 102.49 million in the ward (95% CI = 87.12, 119.32), while in males, the figure was 47.02 million at the lumbar spine (95% CI = 33.22, 66.01) (Tables S9–10 in the **Online Supplementary Document**).

Under the CHN criteria, measuring osteoporosis at the ward site showed the highest prevalence in both sexes. 29.49% (95% CI = 18.29, 43.5) for the total population, 25.92% (95% CI = 18.47, 34.72) for females, and 32.97% (95% CI = 18.11, 52.08) for males. In females, the prevalence increased from 0.61% in the 20–24 age group (95% CI = 0.26, 1.46) to 96.27% in the 85–90 age group (95% CI = 92.04, 98.29), while in males, it increased from 9.85% (95%CI = 3.8, 23.17) to 80.88% (95% CI = 62.36, 91.52). When applying these estimates to the 2020 Chinese population size, there were 317.54 million cases of osteoporosis in China at the ward (95% CI = 196.95, 468.47), including 137.92 million cases in females (95% CI = 98.26, 184.73) and 179.62 million cases in males (95% CI = 98.69, 283.74) (Tables S9–10 in the **Online Supplementary Document**).

### Osteoporosis-associated factors among the Chinese population

**Figure 4:**
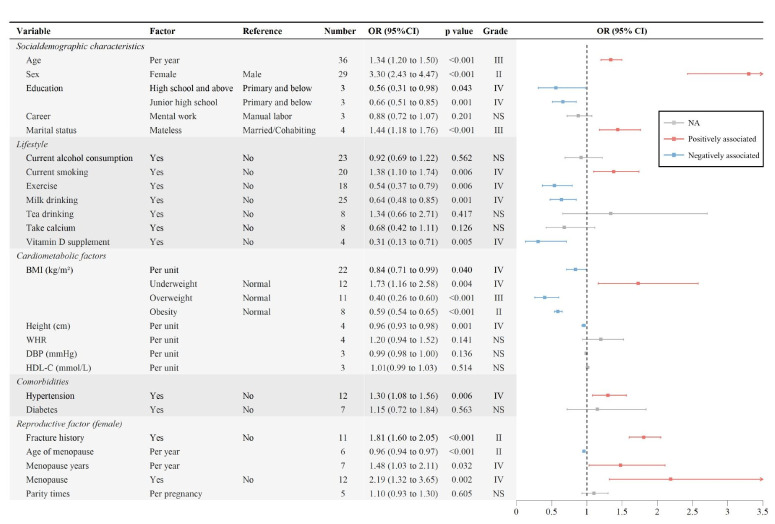
Pooled odds ratios and evidence grade for the associated factors of osteoporosis. BMI – body mass index, DBP – diastolic blood pressure, HDL-C – high-density lipoprotein cholesterol, WHR – waist-hip rate.

After clustering all reported associated factors for osteoporosis by their definitions, we found that 24 individual factors were investigated in at least three studies and thus included in our analysis. Apart from older age and females, we found another seven factors were positively associated with osteoporosis, including marital status, current smoking, underweight, hypertension, fracture history, longer menopause years, and menopause. Also, seven other factors were negatively associated with osteoporosis, including higher education, exercise, milk drinking, vitamin D supplements, higher BMI, larger height, and older age of menopause (**Figure 4**; Figure S1 in the **Online Supplementary Document**).

### Regional prevalence and cases of osteoporosis in China in 2020

**Figure 5:**
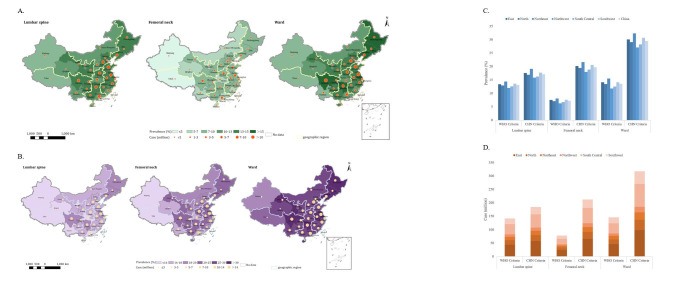
Estimated regional prevalence and cases of osteoporosis by skeletal site in China in 2020. **Panel A.** Geographical distribution of regional prevalence and cases according to WHO diagnostic criteria. **Panel B.** Geographical distribution of regional prevalence and cases according to CHN diagnostic criteria. **Panel C.** Comparison of regional prevalence under WHO and CHN diagnostic criteria. **Panel D.** Comparison of regional case counts under WHO and CHN diagnostic criteria.

Under the WHO criteria, among the six geographic regions, Northeast China had the highest overall prevalence at 15.50% (95% CI = 11.78, 20.59), with 22.22% in females (95% CI = 18.80, 25.95) and 8.68% in males (95% CI = 4.66, 15.16) when measured at the ward site. East China had the highest overall number of cases, totalling 46.36 million (95% CI = 35.26, 61.66), including 32.76 million females (95% CI = 27.93, 38.04) and 13.60 million males (95% CI = 7.33, 23.62) (**Figure 5**; Tables S11–12 in the **Online Supplementary Document**). When focusing on provinces, Sichuan province had the highest overall prevalence of osteoporosis at 16.14% (95% CI = 12.31, 21.37), with 22.67% in females (95% CI = 19.43, 26.17) and 9.67% in males (95% CI = 5.25, 16.61). Shandong province had the highest overall number of cases, totalling 11.07 million (95% CI = 8.46, 14.65), including 7.95 million females (95% CI = 6.77, 9.22) and 3.13 million males (95% CI = 1.69, 5.43) (**Figure 5**; Tables S13–14 in the **Online Supplementary Document**).

Under the CHN criteria, among the six geographic regions, Northeast China had the highest overall prevalence at 32.36% (95% CI = 20.33, 46.8), with 29.86% in females (95% CI = 21.22, 39.76) and 34.9% in males (95% CI = 19.43, 53.94) when measured at the ward site. East China had the highest overall number of cases, totalling 98.77 million (95% CI = 61.81, 144.45), including 43.77 million females (95% CI = 31.37, 58.27) and 55 million males (95% CI = 30.44, 86.18) (**Figure 5**; Tables S11–12 in the **Online Supplementary Document**). When focusing on provinces, Sichuan province had the highest prevalence of osteoporosis at 33.47% (95% CI = 21.25, 48.22), with 29.84% in females (95% CI = 21.63, 39.28) and 37.08% in males (95% CI = 20.86, 57.09). Guangdong province had the highest overall number of cases, totalling 24.33 million (95% CI = 14.39, 37.84), including 9.42 million females (95% CI = 6.54, 13.04) and 14.92 million males (95% CI = 7.85, 24.80) (**Figure 5**; Tables S13–14 in the **Online Supplementary Document**).

## DISCUSSION

Based on a comprehensive systematic review of 129 articles on the prevalence of osteoporosis conducted in China, this model-based study estimated the prevalence of osteoporosis among Chinese adults by diagnostic criteria, age, gender, and skeletal sites. For both genders, the prevalence of osteoporosis increased with advanced age and in all skeletal sites. A slightly higher prevalence could be observed when the diagnosis was based on the CHN criteria than the WHO criteria. When the WHO diagnostic criteria were used, the highest prevalence in males was 8.63% in the lumbar spine, with 47.02 million cases, while in females, the highest prevalence was 19.26% at the ward, with 102.49 million cases. Whereas, based on the CHN diagnostic criteria, both males and females reached the highest prevalence at the ward. The highest osteoporosis prevalence in males was 32.97%, translating to 179.62 million cases, and the highest prevalence in females was 25.92%, with an estimated 137.92 million cases. Furthermore, marital status, current smoking, underweight, hypertension, fracture history, longer menopause years and menopause were confirmed to be associated with a higher risk of osteoporosis. Regarding these two different diagnostic criteria, the populations of the Northeast China region and Sichuan province had the highest prevalence of osteoporosis in the ward.

A recent study revealed that age-standardised disability-adjusted life years due to osteoporosis in middle socio-demographic index countries were estimated to be 200 per 100 000 population in 2020 [33]. Our finding indicated that the prevalence of osteoporosis increased with age and was higher among the older female population in China, which was similar to earlier cross-sectional studies and meta-analyses [23,34–38]. With the ongoing growth of the elderly population in China, the burden of osteoporosis is expected to increase significantly in the next few decades, which is causing a pressing public health challenge. The relationship of osteoporosis with age is mainly due to a reduction of bone formation and low bone turnover, leading to cortical porosity [39]. Generally, the older female population suffers from more severe osteoporosis than the male population. The prevalence of osteoporosis in females increased sharply after the age of 50, with more than three-quarters of females aged >85 years old suffering from osteoporosis. A possible explanation is the rapid hormonal change in postmenopausal women, during which the deficiency of oestrogen is unable to fill in the gap between the low rate of bone formation and the high rate of bone resorption, disrupting skeletal homeostasis and resulting in a rapid cortical thinning and low bone mineral density [40–42]. Since men have thicker cortices than women, the target female cortical bone loss during menopause explains the reason for the higher osteoporosis prevalence in the older woman population [41]. Regarding the age-related risk of osteoporosis, it is important to strengthen the detection of osteoporosis among the elderly population. For instance, policymakers could stipulate that bone mineral density detection should be included in the physical examination of older adults who have reached a certain age. As for the gender disparity, women should pay more attention to osteoporosis and receive timely treatment after being diagnosed, and providing corresponding testing and economic benefits for women could contribute to this certain situation.

Furthermore, we also discovered a difference when diagnosing osteoporosis at the three different skeletal sites. A higher prevalence can be observed at a lumbar spine and ward for both diagnostic criteria, which is in line with cross-sectional results in both Caucasian females and the Chinese population [43,44]. One potential mechanism lies in the difference between the two types of osseous tissue. As cancellous bone mass, the ward and lumbar spine turnover is much faster than cortical bone turnover, this leads to a continuous, progressive bone loss [45,46]. The differences between diagnostic skeletal sites highlight the significance of establishing more uniform diagnostic criteria. To be specific, when allocating resources and formulating policies based on the prevalence of osteoporosis between regions and genders, it should be noted that the dependent data are derived from the same skeletal site. This is conducive to a more rational allocation of resources, including medical resources, financial support and so on.

Opposed to the previous meta-analysis, the prevalence of osteoporosis diagnosed with the CHN criteria led to slightly higher estimates than the WHO criteria, especially for men assessed at the ward [23]. One possible explanation is that the previous meta-analysis included not only the general population but also the data from hospitals, which had been excluded from our analysis [23]. The difference between the prevalence of the two diagnosis criteria brings undiagnosed Chinese osteoporosis patients with BMD between 2.0–2.5 SDs into the count. Furthermore, the difference may also indicate that the WHO diagnosis criteria, which were originally applied to Caucasian women, might not be appropriate for the Chinese general population [47–49] because Asians tend to have slightly lower BMD than Caucasians [50]. Nevertheless, the limited samples of our study need to be considered, too, and further elaboration on the BMD and diagnosis criteria of the Chinese general population may contribute to some extent.

Apart from advanced age and female gender, we also identified marital status, current smoking, underweight, hypertension, fracture history, longer menopause years and menopause as the factors positively associated with osteoporosis. All the associated factors in our study were also identified as relevant in previous studies [34,41,51,52], especially the postmenopausal status, hypertension and current smoking [6,7,53]. Another key insight of our analysis is that high BMI, high education level, height, exercise, milk drinking, vitamin D supplements, and later age of menopause were confirmed to be protective against osteoporosis. Our findings were mostly in accordance with published evidence [54–58]. However, some researchers argued that the benefit of vitamin D can only be observed in individuals with low intake and under the conditions of calcium stress or deficiency [59–61]. Hence, future studies should pursue further the association between the intake of vitamin D, calcium and osteoporosis. Considering the risk factors for osteoporosis mentioned above, policymakers and relative medical workers should lay emphasis on osteoporosis tests for individuals with risk factors. While informing people to avoid risk factors, a more comprehensive and detailed examination for high-risk groups should be carried out.

A multicentre, large-scale study conducted by Zeng and his colleagues discovered that, compared with the other four provinces nationwide, the population in southwest China (Sichuan province) had the highest prevalence of osteoporosis. This is very similar to our results [44]. The regional prevalence differences in China are mainly due to geographical factors, such as sunlight, climate, and height. The diverse lifestyles and food patterns may also contribute. The multicentre study mentioned above also discovered that the northeast China (Liaoning province) population had the lowest prevalence of osteoporosis, which we couldn’t confirm [44]. Our data in northeast China are mostly gathered in Heilongjiang province, so the confined population and the coverage limitation in both our study and Zeng’s study might explain the difference.

To the best of our knowledge, this study provided the first nationwide systematic analysis of the prevalence and associated factors of osteoporosis based on consistent diagnostic criteria that used testing of the skeletal sites in the general population. This study shed light on the at-risk population, prompting postmenopausal women and men aged >50 years to add BMD tests to their regular health checks, especially for those with positively associated factors that were identified. Despite the fact of growing burden of disease and the threat to the Chinese health care system and society, the hazards of osteoporosis remain underestimated, and the awareness of osteoporosis in Chinese people still needs to improve. To prevent osteoporosis on a regular basis, experts suggest that people should maintain a well-balanced diet with appropriate calcium and dairy supplements, keep BMI>20 kg/m^2^, exercise regularly, and cease to smoke [41]. Likewise, measures for fall prevention should be taken to avoid fractures, the main complication of osteoporosis.

Benefiting from a comprehensive search strategy and dual review process, our study pooled data from 129 articles, including more information than any previous meta-analysis of osteoporosis prevalence in the general Chinese population. The abundant information enabled us to conduct a reliable investigation nationwide and extrapolate study results to the general population. Another strength of our study is that we adopted two widely recognised diagnostic criteria of osteoporosis in three certain skeletal sites so that the comparison between studies and over time can be ensured. When assessing the potential associated factors with osteoporosis, the bias inherent to univariate study design can be reduced by only including the studies based on multivariate design.

However, several limitations of our study should not be ignored. Despite our dedicated efforts to determine the osteoporosis prevalence in each province of China, included studies were limited in terms of coverage, particularly in central and western China. Most of the included studies were conducted in the capital city of each province, neglecting the rural areas where osteoporosis prevalence is higher [34]. This oversight may lead to a substantial underestimation of osteoporosis prevalence in the central and western provinces of China. Policymakers should consider providing additional medical funding and allocating more health care personnel to rural areas to improve osteoporosis screening in these populations and gather comprehensive epidemiological data. This effort is of great importance for future research and related practical initiatives. Meanwhile, the lack of standardised diagnosis criteria, measurement methods, and skeletal sites over the past few decades created additional challenges. When analysing the factors associated with osteoporosis, we did not adhere to a specific definition of osteoporosis. This lack of standardisation may have introduced biases to our results. However, it provided an opportunity to explore as many potential factors as possible that could be associated with the risk of osteoporosis. Therefore, to better explore the finer dimensions and identify risk factors, a scale-up of high-quality data with well-accepted diagnostic criteria and an approach to the epidemiology of osteoporosis is required.

## CONCLUSIONS

In conclusion, we analysed the associated factors of osteoporosis and estimated its future prevalence. We revealed that osteoporosis will continue to be a serious public health problem in China, and the burden on females is more serious, requiring attention. In the future, greater priority and related resources for osteoporosis from the government and relevant departments will be required.

## Additional material


Online Supplementary Document
