# Supplementary material for: Prevalence of osteoporosis and associated factors among Chinese adults: a systematic review and modelling study
Source: J Glob Health. 2025 Jan 17;15:04009. doi: 10.7189/jogh.15.04009 (PMC11737814; doi:10.7189/jogh.15.04009)
Supplement: Online Supplementary Document [file jogh-15-04009-s001.pdf]

Supplement to: Liu Y, Huang X, Tang K, Wu J, Zhou J, Bai H, Zhou L, Shan S, Luo Z, Cao J, Song P, Rudan I; on behalf of the Global Health Epidemiology Research Group (GHERG). Prevalence of osteoporosis and associated factors among Chinese adults: a systematic review and modelling study. J Glob Health. 2025;15:04009.

Supplementary Appendix

Table S1. Search Strategies in different bibliographic databases 2
Table S2. Quality assessment scale for rating the risk of bias 4
Table S3. Meta-regression models of cluster-level factors related to the prevalence of osteoporosis 5
Table S4. Evidence credibility grading criteria 8
Table S5. Provinces in geographical regions of mainland China 9
Table S6. Full list of the included articles (n=129) 10
Table S7. Quality scores for assessing the risk of bias in the included articles (n=129) 17
Table S8. Characteristics of included article 22
Table S9. Estimated age- and sex-specific prevalence of osteoporosis in China in 2020 31
Table S10. Estimated age- and sex-specific number of cases of osteoporosis in China in 2020 33
Table S11. Estimated geographical regions- and sex-specific prevalence of osteoporosis in China in 2020 35
Table S12. Estimated geographical regions- and sex-specific number of cases of osteoporosis in China in 2020 36
Table S13. Estimated provinces- and sex-specific prevalence of osteoporosis in China in 2020 37
Table S14. Estimated provinces- and sex-specific number of cases of osteoporosis in China in 2020 41
Figure S1. Meta-analyses of associated factors for osteoporosis 45
eMothed. Detailed process of the estimation of the prevalence of osteoporosis 59
Reference 62

This supplementary material has been provided by the authors to give readers additional information about their work.

Table S1. Search Strategies in different bibliographic databases

Database	Access date	Subject category	Sub-database	Search terms	Number of Identified records
CNKI	27th February, 2022	Medicine & Public Health	Journal, Featured journal, Doctoral dissertation, Master dissertation	(SU%='骨量减少'+'骨质疏松'+'骨质丢失') AND (SU%='发病率'+'发生率'+'患病率'+'罹患率'+'现患率'+'流行'+'现况')<br>(SU%='guliangjianshao'+'guzhishusong'+'guzhidiushi') AND (SU%='fabinglv'+'fashenglv'+'huanbinglv'+'lihuanlv'+'xianhuanlv'+'liuxing'+'xiankuang')	5742
Wanfang	27th February, 2022	Medicine & Public Health	Journal articles, Dissertations	((主题:"骨量减少" OR 主题:"骨质疏松" OR 主题:"骨质丢失") AND (主题:"发病率" OR 主题:"发生率" OR 主题:"患病率" OR 主题:"现患率" OR 主题:"罹患率"))<br>((subject:"guliangjianshao" OR subject:"guzhishusong" OR subject:"guzhidiushi") AND (subject:"fabinglv" OR subject:"fashenglv" OR subject:"huanbinglv" OR subject:"xianhuanlv" OR subject:"lihuanlv"))	11064
CQVIP	27th February, 2022	Medicine & Public Health	Not applicable	(M=(骨量减少 OR 骨质疏松 OR 骨质丢失) OR R=(骨量减少 OR 骨质疏松 OR 骨质丢失)) AND (M=(发病率 OR 发生率 OR 患病率 OR 现患率 OR 罹患率 OR 流行 OR 现况) OR R=(发病率 OR 发生率 OR 患病率 OR 现患率 OR 罹患率 OR 流行 OR 现况))<br>(M=(guliangjianshao OR guzhishusong OR guzhidiushi) OR R=(guliangjianshao OR guzhishusong OR guzhidiushi)) AND (M=(fabinglv OR fashenglv OR huanbinglv OR xianhuanlv OR lihuanlv OR liuxing OR xianzhuang) OR R=(fabinglv OR fashenglv OR huanbinglv OR xianhuanlv OR lihuanlv OR liuxing OR xianzhuang))	10389
Pubmed	27th February, 2022	Not applicable	Not applicable	(((((osteopenia[Title/Abstract] OR osteoporosis[Title/Abstract] OR OP[Title/Abstract] OR bone density[Title/Abstract] OR bone mineral density[Title/Abstract] OR BMD[Title/Abstract] OR bone loss[Title/Abstract])) AND (inciden*[Title/Abstract] OR prevalen*[Title/Abstract] OR epidemiolog*[Title/Abstract])) AND (China[Title/Abstract] OR Chinese[Title/Abstract] OR Hongkong[Title/Abstract] OR Macao[Title/Abstract] OR Taiwan[Title/Abstract])) AND (humans[Filter])) AND (("1990/01/01"[Date - Publication] : "2022/02/27"[Date - Publication]))	679

Database	Access date	Subject category	Sub-database	Search terms	Number of Identified records
Embase	27th February, 2022	Not applicable	Not applicable	1 'osteoporosis'/exp OR 'osteoporosis':ab,ti OR 'op':ab,ti<br>2 'osteopenia'/exp OR 'osteopenia':ab,ti<br>3 'bone density'/exp OR 'bone mineral density':ab,ti OR 'bmd':ab,ti OR 'bone loss':ab,ti<br>4 'china'/exp OR chinese:ab,ti<br>5 'hongkong':ab,ti OR 'macao':ab,ti OR 'taiwan':ab,ti<br>6 'prevalence'/exp OR prevalen*:ab,ti<br>7 'incidence'/exp OR inciden*:ab,ti<br>8 'epidemiology'/exp OR epidemiolog*:ab,ti<br>9 #1 OR #2 OR #3<br>10 #4 OR #5<br>11 #6 OR #7 OR #8<br>12 #9 AND #10 AND #11<br>13 #9 AND #10 AND #11 AND [humans]/lim AND [1990-2022]/py<br>14 #13 AND [embase]/lim NOT ([embase]/lim AND [medline]/lim)	809
Medline	27th February, 2022	Not applicable	Not applicable	1 exp osteoporosis/<br>2 osteoporosis. ab,ti. or osteopenia. ab,ti. or OP. ab,ti.<br>3 exp bone density/<br>4 bone mineral density. ab,ti. or BMD. ab,ti.<br>5 bone loss. ab,ti.<br>6 exp prevalence/or prevalen*.ab,ti<br>7 exp incidence/or inciden*.ab,ti<br>8 exp epidemiology/or epidemiolog*.ab,ti<br>9 exp China/or Chinese.ab,ti<br>10 Hongkong. ab,ti. or Macao. ab,ti. or Taiwan. ab,ti.<br>11 1 or 2 or 3 or 4 or 5<br>12 6 or 7 or 8<br>13 9 or 10<br>14 11 and 12 and 13<br>15 limit 14 to (humans and yr="1990 -Current"	871

Table S2. Quality assessment scale for rating the risk of bias

Bias type	Low risk (score=2)	Moderate risk (score=1)	High risk (score=0)
Selection (sample population)	1) Sample from the general population, not a select group;<br>2) Consecutive unselected population;<br>3) Rationale for case and control selection explained.	1) Sample selected from large population but selection criteria not defined;<br>2) Sample selection ambiguous but may be representative;<br>3) Rationale for cases and. controls not explained;<br>4) Eligibility criteria not explained;<br>5) Analysis to adjust for sampling strategy bias.	1) Highly select population making it difficult to generalize finding;<br>2) Sample selection ambiguous and sample unlikely to be representative.
Selection (sample size)	1) Sample size calculation performed and adequate.	1) Sample size calculation performed and reasons for not meeting sample size given;<br>2) Sample size calculation not performed but all eligible persons studied.	1) Sample size estimation unclear or only sub-sample studied.
Selection (participation rate)	1) High response rate (>85%).	1) Moderate response rate (70-85%).	1) Low response rate (<70%);<br>2) Response rate not reported.
Performance bias (outcome assessment)	1) Diagnosis using consistent criteria and direct examination.	1) Assessment from administrative database or register;<br>2) Assessment from hospital record or interviewer.	1) Assessment from non-validated data or generic estimate from the overall population.
Performance bias (analytical methods to control for bias)	1) Analysis appropriate for the type of sample (subgroup analysis/regression etc.).	1) Analysis does not account for common adjustment.	1) Data confusing.

Table S3. Meta-regression models of cluster-level factors related to the prevalence of osteoporosis

Variable	No. of data points	β	95% CI		*P*-value	No. of data points	β	95% CI		*P*-value
**Univariable meta-regression**										
**WHO diagnostic criteria**										
	**Male**					**Female**				
**Lumbar spine**										
Age	41	0.0235	0.0146	0.0323	<0.0001	83	0.0726	0.0686	0.0765	<0.0001
Publication year	41	-0.0134	-0.0633	0.0365	0.5981	83	-0.0138	-0.0483	0.0207	0.4325
Investigation year	41	-0.0125	-0.0628	0.0378	0.6265	83	-0.0128	-0.0467	0.0212	0.461
Study setting										
Mixed	20	Reference				54	Reference			
Urban	21	0.1764	-0.5555	0.9083	0.6367	21	0.0106	-0.5718	0.5931	0.9715
Rural	0		—			8	0.1613	-0.972	1.2945	0.7803
Latitude	41	0.0189	-0.0311	0.0689	0.4584	78	-0.0153	-0.0567	0.0261	0.4686
**Femoral neck**										
Age	62	0.0642	0.0522	0.0762	<0.0001	91	0.122	0.1144	0.1296	<0.0001
Publication year	62	-0.0708	-0.2322	0.0907	0.3904	91	-0.0313	-0.0968	0.0343	0.3499
Investigation year	62	-0.0367	-0.174	0.1007	0.6008	91	-0.0304	-0.0911	0.0302	0.3256
Study setting										
Mixed	45	Reference				62	Reference			
Urban	17	-0.828	-2.1624	0.5064	0.2239	21	-0.89	-1.5699	-0.2102	0.0103
Rural	0		—			8	-0.5366	-1.8898	0.8166	0.437
Latitude	62	-0.0346	-0.1286	0.0595	0.4712	91	-0.0251	-0.0808	0.0306	0.3775
**Ward's triangle**										
Age	50	0.0798	0.0712	0.0883	<0.0001	74	0.1468	0.1403	0.1534	<0.0001
Publication year	50	-0.0433	-0.2225	0.1358	0.6354	74	-0.0701	-0.1495	0.0093	0.0837
Investigation year	50	-0.0017	-0.1585	0.1552	0.9832	74	-0.0451	-0.1258	0.0356	0.2736
Study setting										

Mixed	46	Reference				62	Reference			
Urban	4	-1.4012	-3.1588	0.3564	0.1182	4	-1.437	-2.4363	-0.4376	0.0048
Rural	0		—			8	-0.3457	-1.6728	0.9814	0.6097
Latitude	46	-0.0699	-0.1869	0.047	0.2413	70	-0.0602	-0.1246	0.0043	0.0673
**CHN diagnostic criteria**										
			**Male**					**Female**		
**Lumbar spine**										
Age	11	0.0499	0.0296	0.0701	＜0.0001	29	0.0886	0.081	0.0962	＜0.0001
Publication year	11	-0.0022	-0.0489	0.0445	0.9263	29	-0.0172	-0.0698	0.0355	0.5228
Investigation year	11	-0.0022	-0.0489	0.0445	0.9263	29	-0.0136	-0.0663	0.0391	0.6138
Study setting										
Mixed	6	Reference				19	Reference			
Urban	5	-0.1926	-0.7448	0.3597	0.4943	10	-0.3337	-0.8685	0.2011	0.2214
Rural	0		—			0		—		
Latitude	11	0.0345	-0.0035	0.0724	0.0750	29	0.0074	-0.0464	0.0612	0.7887
**Femoral neck**										
Age	18	0.0527	0.044	0.0614	＜0.0001	37	0.0933	0.0841	0.1025	＜0.0001
Publication year	18	-0.0412	-0.13	0.0475	0.3624	37	0.0331	-0.2658	0.332	0.8281
Investigation year	18	-0.0413	-0.1524	0.0698	0.4662	37	0.0363	-0.2907	0.3633	0.8277
Study setting										
Mixed	18	Reference				32	Reference			
Urban	0		—			5	0.059	-3.5025	3.6206	0.9741
Rural	0		—			0		—		
Latitude	18	0.0364	-0.0134	0.0862	0.1519	37	0.1132	-0.035	0.2614	0.1345
**Ward's triangle**										
Age	14	0.0563	0.0454	0.0671	＜0.0001	33	0.1283	0.1152	0.1415	＜0.0001
Publication year	14	0.1514	0.0679	0.235	0.0004	33	0.0982	-0.3092	0.5056	0.6367
Investigation year	14	0.2050	0.1029	0.3072	＜0.0001	33	0.1004	-0.3566	0.5574	0.6667
Study setting										

Mixed	14	Reference				33	Reference			
Urban	0					0			—	
Rural	0			—		0			—	
Latitude	14	-0.1068	-0.1896	-0.024	0.0114	33	0.134	0.0047	0.2633	0.0422
**Multivariable meta-regression**										
**WHO diagnostic criteria**										
		**Male**					**Female**			
**Lumbar spine**										
Intercept	41	-3.5134	-4.1902	-2.8366	＜0.0001	83	-5.4009	-5.7444	-5.0575	＜0.0001
Age	41	0.0235	0.0146	0.0323	＜0.0001	83	0.0726	0.0686	0.0765	＜0.0001
**Femoral neck**										
Intercept	62	-6.3901	-7.4389	-5.3413	＜0.0001	91	-9.4408	-10.1678	-8.7137	＜0.0001
Age	62	0.0642	0.0522	0.0762	＜0.0001	91	0.122	0.1144	0.1296	＜0.0001
**Ward's triangle**										
Intercept	50	-6.8245	-7.7344	-5.9145	＜0.0001	74	-9.6827	-10.2149	-9.1505	＜0.0001
Age	50	0.0798	0.0712	0.0883	＜0.0001	74	0.1468	0.1403	0.1534	＜0.0001
**CHN diagnostic criteria**										
		**Male**					**Female**			
**Lumbar spine**										
Intercept	11	-4.6775	-6.028	-3.3271	＜0.0001	29	-5.8598	-6.4686	-5.251	＜0.0001
Age	11	0.0499	0.0296	0.0701	＜0.0001	29	0.0886	0.081	0.0962	＜0.0001
**Femoral neck**										
Intercept	18	-4.0163	-4.6449	-3.3877	＜0.0001	37	-6.5008	-7.6382	-5.3635	＜0.0001
Age	18	0.0527	0.044	0.0614	＜0.0001	37	0.0933	0.0841	0.1025	＜0.0001
**Ward's triangle**										
Intercept	14	-3.452	-4.5954	-2.3086	＜0.0001	33	-7.9132	-8.9834	-6.843	＜0.0001
Age	14	0.0563	0.0454	0.0671	＜0.0001	33	0.1283	0.1152	0.1415	＜0.0001

Table S4. Evidence credibility grading criteria

Category	Criteria
Convincing evidence (class I)	1) *P*-value $< 1\times10^{-6}$<br>2) More than 1000 cases<br>3) 95% prediction interval excluding the null value<br>4) $I^2 < 50\%$<br>5) No small-study effects and excess significance bias
Highly suggestive evidence (class II)	1) *P*-value $< 1\times10^{-6}$<br>2) More than 1000 cases<br>3) A statistically significant result reported in the largest
Suggestive evidence (class III)	1) *P*-value $< 1\times10^{-3}$<br>2) More than 1000 cases
Weak evidence (class IV)	1) *P*-value < 0.05
Non-significant (NS)	1) *P*-value > 0.05

Table S5. Provinces in geographical regions of mainland China

Geographical regions	Province
North	Beijing
	Tianjin
	Hebei
	Shanxi
	Inner Mongolia
East	Shanghai
	Jiangsu
	Zhejiang
	Anhui
	Fujian
	Jiangxi
	Shandong
South Central	Henan
	Hubei
	Hunan
	Guangdong
	Guangxi
	Hainan
Southwest	Chongqing
	Sichuan
	Guizhou
	Yunnan
	Tibet
Northwest	Shaanxi
	Gansu
	Qinghai
	Ningxia
	Xinjiang
Northeast	Liaoning
	Jilin
	Heilongjiang

Table S6. Full list of the included articles (n=129)

ID	Reference
1	沈惠良,雍宜民,周玉芳.北京市老年人腰椎与前臂骨密度的调查及相关分析[J].中国骨质疏松杂志,1996,(01):18-22.
2	汤健,叶冬青,张建湘等.合肥市郊区骨质疏松症的现况研究[J].中国骨质疏松杂志,1997,(02):36-38.
3	廖二元,伍贤平,邓小戈等.对中国长沙地区女性骨密度情况的调查[J].中华内分泌代谢杂志,2000,(04):5-9.
4	王文志,马锦富,杨定焯等.成都地区中老年人群骨密度调查[J].中国骨质疏松杂志,2000,(01):43-46.
5	吴青,陶国枢,牟善初.老年骨质疏松症患病率的调查[J].解放军保健医学杂志,2000(03):18-19.
6	余建国,姚安晋,张东等.绝经后骨质疏松症腰椎骨密度研究[J].实用放射学杂志,2000(01):35-36.
7	潘京海. 原发性骨质疏松症患病及影响因素研究[D]. 北京:北京大学,2001.
8	颜晓东,黄忠,朱敏嘉等.绝经后妇女血清调钙激素水平与骨代谢关系探讨[J].中国骨质疏松杂志,2001,(03):20-22.
9	周乃珍,陈治卿,余玲玲等.老年男性骨密度值与体重、体重指数关系探讨[J].浙江医学,2001(03):16-17.
10	安珍,王文志,杨定焯等.成都地区城乡人群原发性骨质疏松调查[J].中国骨质疏松杂志,2002,(03):46-49.
11	陈友华.绝经后女性骨密度测定的临床价值与护理探讨[J].护士进修杂志,2002,(01):9-10.DOI:10.16821/j.cnki.hsjx.2002.01.004.
12	聂伟志. 上海地区妇女骨密度的生物力学影响因素及股骨颈抗骨折能力的初步调查[D]. 上海:上海中医药大学,2002.
13	王平芳,杨雅,廖二元等.长沙农村中老年妇女骨密度调查[J].中国医师杂志,2002,(12):1307-1310.
14	王锐,孔西建,孟庆阳.健康女性腰椎骨密度与年龄和绝经的关系[J].中医正骨,2002(05):13-14+64.
15	林伟,邓力平,邬恒夫等.广州地区 1530 例骨密度分析及骨质疏松发病率研究[J].中国骨质疏松杂志,2003(03):71-72+84.
16	张毅,曾平,王凤兰.北京社区健康成人骨密度及骨质疏松患病调查[J].世界医学杂志,2004,8(5):62-64
17	陈玉平,蔡德鸿,刘雪琴等.社区中老年人骨质疏松症的患病危险因素调查[J].中国临床康复,2005,(15):156-157.
18	李敏. 上海城区 40 岁以上女性骨质疏松患病率及危险因素研究[D]. 上海:复旦大学,2005. DOI:10.7666/d.y952123.
19	周起敬,明庆华,徐汝昌等.昆明女性髋部骨密度及骨质疏松调查[J].中国骨质疏松杂志,2005(01):66-68.
20	Zhang Z L, Qin Y J, Huang Q R, et al. Bone mineral density of the spine and femur in healthy Chinese men[J]. Asian journal of andrology, 2006, 8(4): 419-427.
21	蒙元劲,韦金一,龙柳艳等.柳州市中老年人骨质疏松患病率调查[J].齐齐哈尔医学院学报,2006(15):1851.
22	李毅,于秋滨,陶天遵等.中老年妇女骨质疏松症的流行病学调查[J].中国骨质疏松杂志,2007(04):263-266.
23	马蓓蕾. 中老年妇女骨质疏松营养相关危险因素的研究[D]. 上海:上海交通大学,2007.
24	潘海林,苏宏业,黎英荣等.广西南宁地区成年女性骨密度与年龄关系的研究[J].广西医科大学学报,2007,(06):884-885.DOI:10.16190/j.cnki.45-1211/r.2007.06.025.
25	张毅,曾平,李宁.北京崇文区中老年原发性骨质疏松症的发生及其影响因素:分层多阶段整群抽样调查[J].中国组织工程研究与临床康复,2007,(27):5332-5335.

ID	Reference
26	曾平,张毅,李宁华.北京市东城区 289 名健康中老年人骨密度测量(英文)[J].中国组织工程研究与临床康复,2008(02):397-400.
27	程晓光,杨定焯,周琦等.中国女性的年龄相关骨密度、骨丢失率、骨质疏松发生率及参考数据库——多中心合作项目[J].中国骨质疏松杂志,2008(04):221-228.
28	张盘德,冯彦林,张自茂.佛山地区中老年人骨密度测定及骨质疏松患病率分析[J].中国骨质疏松杂志,2008(08):567-569.
29	陈文远,张寿,丁晓莉.海口地区中老年人骨密度调查研究[J].中国骨肿瘤骨病,2009,8(05):265-267.
30	付玉娟,张超远,聂伟等.南阳城区正常人群骨密度分析及骨质疏松患病率研究[J].中国煤炭工业医学杂志,2009,12(01):30-31.
31	Ho S C, Lau E M C, Woo J, et al. The prevalence of osteoporosis in the Hong Kong Chinese female population[J]. Maturitas, 1999, 32(3): 171-178.
32	Liao E Y, Wu X P, Deng X G, et al. Age-related bone mineral density, accumulated bone loss rate and prevalence of osteoporosis at multiple skeletal sites in Chinese women[J]. Osteoporosis International, 2002, 13: 669-676.
33	Wu X P, Liao E Y, Zhang H, et al. Determination of age-specific bone mineral density and comparison of diagnosis and prevalence of primary osteoporosis in Chinese women based on both Chinese and World Health Organization criteria[J]. Journal of bone and mineral metabolism, 2004, 22: 382-391.
34	Lynn H S, Lau E M C, Au B, et al. Bone mineral density reference norms for Hong Kong Chinese[J]. Osteoporosis international, 2005, 16: 1663-1668.
35	Wu X P, Hou Y L, Zhang H, et al. Establishment of BMD reference databases for the diagnosis and evaluation of osteoporosis in central southern Chinese men[J]. Journal of bone and mineral metabolism, 2008, 26: 586-594.
36	Lin Y C, Pan W H. Bone mineral density in adults in Taiwan: results of the Nutrition and Health Survey in Taiwan 2005-2008 (NAHSIT 2005-2008)[J]. Asia Pacific journal of clinical nutrition, 2011, 20(2): 283-291.
37	孙伟方,裴继强,刘波等.舟山海岛老年人患骨质疏松症影响因素的研究[J].中国预防医学杂志,2009,10(04):263-266.DOI:10.16506/j.1009-6639.2009.04.028.
38	汪明星,房明亮,王建强等.徐州地区 1572 例健康人群骨密度和骨质疏松症调查[J].徐州医学院学报,2010,30(03):167-170.
39	刘辉文,陈劲勇,张国华等.湖南邵阳地区 4000 例健康成人骨量横断面调查及骨质疏松症危险因素分析[J].中国骨质疏松杂志,2011,17(12):1087-1091.
40	刘柳,张巧,彭年春.贵阳市城区成年人骨质疏松症现状调查[J].中华骨质疏松和骨矿盐疾病杂志,2011,4(02):108-112.
41	陶黎,王翠侠,刘颖等.北京老年妇女膳食钙摄入水平与骨量关系[J].中国骨质疏松杂志,2011,17(05):386-388.
42	田峰. 基于广义偏线性模型的 40~65 岁女性原发性骨质疏松症筛检工具研究[D]. 北京:中国中医科学院,2011.
43	涂萍,邓波,徐定波等.南昌地区 20 岁以上人群骨量变化规律及与骨代谢指标相关性研究[J].中国骨质疏松杂志,2011,17(03):249-252.
44	吴非同,胡传来,方炎福等.社区中老年人群骨质疏松影响因素研究[J].中国全科医学,2011,14(14):1540-1542.

ID	Reference
45	孙凤,郁凯,陶庆梅等.台湾 35～74 岁男性体检者骨质疏松 5 年发病风险预测模型[J].中国骨质疏松杂志,2012,18(10):905-911.
46	王晨秀,霍亚南,林安华等.南昌市部分社区中老年女性骨质疏松流行现状调查及影响因素分析[J].江西医药,2012,47(12):1037-1040.
47	杨妍.北京市区中老年女性骨量降低危险因素的筛查评估分析[D].首都体育学院,2012.
48	李绒,楼婷.高学历中年男性骨质疏松影响因素调查[J].中国康复理论与实践,2013,19(03):280-282.
49	阮璐雅,章海凌,郑景晨等.温州市社区绝经后妇女骨质疏松症发生因素分析[J].中国乡村医药,2013,20(23):61-62.DOI:10.19542/j.cnki.1006-5180.2013.23.033.
50	吴凌云,马红梅,唐世琪.武汉地区老年人骨质疏松患病率及其影响因素[J].职业与健康,2013,29(22):2892+3057.DOI:10.13329/j.cnki.zyyjk.2013.22.061.
51	徐光铮,张惠琴,王慧.1504 名徐家汇街道 65 岁以上老人骨质疏松调查及相关因素分析[J].上海医药,2013,34(16):51-53.
52	张珍珍. 皖北地区 40～65 岁女性骨质疏松危险因素分析[D].安徽医科大学,2014.
53	Lo S S T. Bone health status of postmenopausal Chinese women[J]. Hong Kong Medical Journal, 2015, 21(6): 536.
54	Lee C Y, Chen P E, Tung T H. Clinical epidemiology of osteoporosis among elderly fishing and agricultural population in Taipei, Taiwan[J]. Journal of Men's Health, 2020, 16(1): 53-62.
55	张付坤,王永峰,刘光义等.某市社区中老年人群骨质疏松影响因素研究[J].安徽医药,2014,18(04):637-640.
56	寇利琼.老年女性骨质疏松相关危险因素分析[J].华西医学,2014,29(08):1457-1460.
57	宫笑微,李荣滨,高飞等.黑龙江省西部地区骨质疏松流行病学调查[J].齐齐哈尔医学院学报,2014,35(21):3204-3205.
58	黄雪良,黄仕春,黄浩等.深圳市社区老人骨质疏松状况及综合干预效果[J].职业与健康,2014,30(23):3441-3443.DOI:10.13329/j.cnki.zyyjk.2014.0040.
59	罗巧彦,杨茂君,徐勇等.长江上游女性骨质疏松患病率及相关危险因素调查[J].中国骨质疏松杂志,2014,20(07):833-838.
60	田静,仝林虎.内蒙古西部地区蒙古族骨质疏松症的发病及其危险因素分析[J].中国骨质疏松杂志,2014,20(12):1473-1477.
61	庞丹丹. 吉林省德惠市成人骨质疏松症现状调查及影响因素分析[D].吉林大学,2015.
62	寇南楠. 云南省德宏州傣族人群骨质疏松症流行病学调查及相关影响因素研究分析[D].昆明医科大学,2017.
63	李晓莲. 汕头市社区中老年人群骨质疏松症患病情况及影响因素研究[D]. 广东:汕头大学,2016.
64	石益斌,郑松柏,沈利岩等.上海市某社区老年人群骨质疏松现况及相关因素分析[J].上海预防医学,2016,28(01):24-29.DOI:10.19428/j.cnki.sjpm.2016.01.007.
65	赵兰芳,王俊,王雪君.城市社区居民骨质疏松症影响因素分析[J].预防医学,2016,28(10):984-986+991.DOI:10.19485/j.cnki.issn1007-0931.2016.10.004.
66	杨通宇,陈新春,刘照时等.少数民族地区农村女性骨质疏松患病及影响因素分析[J].中国公共卫生,2016,32(03):266-269.

ID	Reference
67	范文强,耿秀琴,边彩月等.新乡市部分社区人群骨质疏松症流行病学调查及相关影响因素 Logistic 回归分析[J].中国骨质疏松杂志,2016,22(02):179-182.
68	唐毅,杨靖,曹洪义等.成都地区不同年龄城乡妇女维生素 D 和骨代谢指标及骨密度的状况分析[J].中国骨质疏松杂志,2016,22(06):756-760.
69	林玲,穆海祥,蔡波.南通市中老年骨质疏松现状及影响因素[J].中国老年学杂志,2016,36(24):6261-6263.
70	刘斌.甘肃省 20-80 岁汉族和裕固族骨质疏松症患病情况及影响因素分析[D].北京协和医学院,2017.
71	齐文雪. 乌鲁木齐天山区 748 例维吾尔族居民跟骨骨密度测定及相关因素探讨[D].新疆医科大学,2017.
72	杨睿斐. 甘肃省成人骨质疏松流行病学调查及相关因素分析[D].宁夏医科大学,2018.
73	赵永琴. 山西省太原市部分城区成年人群骨密度相关影响因素的研究[D].山西医科大学,2018.
74	丁雪勇,徐希彦.重庆市南岸区中老年骨质疏松症患病率调查[J].检验医学与临床,2017,14(06):833-834.
75	郈彩珍,杜颖鑫,吴宏霞等.宁夏 1925 名正常体检人群的骨密度及与人体成份的关系[J].宁夏医科大学学报,2017,39(05):559-563.DOI:10.16050/j.cnki.issn1674-6309.2017.05.017.
76	曹刚,胡妍,刘海洋等.银川市西夏区中青年居民骨密度测定结果及早期干预效果分析[J].宁夏医学杂志,2017,39(11):1040-1041.DOI:10.13621/j.1001-5949.2017.11.1040.
77	谭晓霞,陈施晓,杨梓熔等.汕头海岛社区中老年女性骨质疏松症患病现状及其相关因素调查[J].现代预防医学,2017,44(15):2756-2759+2764.
78	姚立彬,张林华,李百占等.邢台农村老年人骨质疏松症患病情况及相关因素研究[J].预防医学情报杂志,2017,33(10):957-960.
79	张军,张清,张凤梅等.济南市城市社区居民骨质疏松症患病情况及其影响因素[J].职业与健康,2017,33(22):3134-3137.DOI:10.13329/j.cnki.zyyjk.2017.0929.
80	覃素娇,罗颖华,罗珍玉等.广西南宁市区绝经后女性骨质疏松症流行情况及相关因素研究[J].中国骨质疏松杂志,2017,23(07):942-946.
81	张恒林,谢文凯,羊才丰等.儋州农村地区老年人群骨质疏松症的患病率及其危险因素分析[J].中国骨质疏松杂志,2017,23(06):812-817.
82	林彦杰,李勇峰,王辉辉.非糖尿病女性绝经后骨质疏松发生率及其独立危险因素分析[J].中国民康医学,2017,29(19):19-21.
83	叶兆莲,张龙英,王云等.海南省人民医院健康体检人群骨质疏松状况及其相关影响因素分析[J].中国医学前沿杂志(电子版),2017,9(06):51-54.
84	王熙然,白玉蓉,裴育等.北京某社区 40 岁以上女性原发性骨质疏松症流行现状调查[J].中华保健医学杂志,2017,19(02):132-134.
85	Ko C H, Yu S F, Su F M, et al. High prevalence and correlates of osteoporosis in men aged 50 years and over: A nationwide osteoporosis survey in Taiwan[J]. International Journal of Rheumatic Diseases, 2018, 21(12): 2112-2118.

ID	Reference
86	Zhang X D, Lin J S, Yang Y, et al. Comparison of three tools for predicting primary osteoporosis in an elderly male population in Beijing: a cross-sectional study[J]. Clinical Interventions in Aging, 2018: 201-209.
87	陈静,方红丽.昆明地区体检人群骨质疏松流行状况及影响因素分析[J].微循环学杂志,2018,28(02):58-60+65.
88	陈婷,唐文革,丁贤彬等.重庆市 40 岁及以上城市居民骨质疏松症现状调查[J].中国慢性病预防与控制,2018,26(05):338-342.DOI:10.16386/j.cjpccd.issn.1004-6194.2018.05.005.
89	范子寒,林吉生,费琦等.社区 1349 例中老年男性骨密度变化规律及骨质疏松患病率调查[J].临床和实验医学杂志,2018,17(22):2453-2456.
90	李荣锐,王天枢,魏巍等.农村地区老年人骨质疏松患病率及影响因素[J].中国老年学杂志,2018,38(14):3534-3537.
91	李炎.广西毛南族、瑶族和苗族成年人骨密度现状及骨质疏松症危险因素的分析[D].广西医科大学,2018.
92	马峥.厦门市中老年居民骨质疏松影响因素调研[J].中国卫生工程学,2018,17(01):53-55.DOI:10.19937/j.issn.1671-4199.2018.01.017.
93	田利民,杨睿斐,魏莲花等.甘肃省绝经女性和老年男性骨质疏松流行病学调查及相关影响因素分析[J].中华骨质疏松和骨矿盐疾病杂志,2018,11(02):142-148.
94	王小舟,胡雪萍,陆海君.老年原发性骨质疏松症危险因素及预防措施探讨[J].中国农村卫生事业管理,2018,38(06):846-847.
95	夏劲节,魏咏兰,曾伟.成都市城区 40 岁及以上女性骨质疏松流行现状及相关因素分析[J].现代预防医学,2018,45(01):54-57.
96	杨立进,陈博来,胡伟雄等.广州市多社区 1529 名中老年人骨质疏松患病率及骨质疏松性骨折发生率分析[J].中国骨质疏松杂志,2018,24(10):1341-1345.
97	朱庆军,李海珍,侯勇强等.煤矿工人骨质疏松流行现状及其影响因素[J].工业卫生与职业病,2018,44(01):32-34.DOI:10.13692/j.cnki.gywsyzyb.2018.01.008.
98	柴波.太原市小店区绝经后妇女骨质疏松症及椎体骨折患病率的流行病学研究[D].山西医科大学,2019.
99	赖培茜.绝经后原发性骨质疏松症影响因素的分析研究[D].广东药科大学,2019.DOI:10.27690/d.cnki.ggdyk.2019.000256.
100	罗艳燕,王婷,何燕.南充市某医院健康体检人群中骨质疏松检出情况及相关因素分析[J].预防医学情报杂志,2019,35(09):995-1000.
101	王德杰,郑傲,潘杰.电网男职工骨质疏松症现状调查及危险因素分析[J].中国现代药物应用,2019,13(12):28-30.DOI:10.14164/j.cnki.cn11-5581/r.2019.12.012.
102	文新强,李楠,王光明.更年期女性骨密度及其影响因素分析[J].中国妇幼健康研究,2019,30(08):964-967.
103	曾佳,兰红勤,王玮.1245 例长沙地区体检人群骨密度现状调查及影响因素分析[J].临床医学研究与实践,2020,5(12):9-11.DOI:10.19347/j.cnki.2096-1413.202012004.
104	顾巧萍,吴丽平,孙微.绝经后骨质疏松症的患病率及危险因素研究[J].中国妇幼保健,2020,35(22):4158-4162.DOI:10.19829/j.zgfybj.issn.1001-4411.2020.22.001.

ID	Reference
105	蓝超华,姚卫光.骨质疏松风险评估模型的构建[J].广西科学,2020,27(06):676-685.DOI:10.13656/j.cnki.gxkx.20200803.001.
106	李金龙.福州鼓楼区中老年人骨质疏松症危险因素及其中医证型的临床研究[D].福建中医药大学,2020.DOI:10.27021/d.cnki.gfjzc.2020.000219.
107	李雪,沈静,杨蕾等.乌鲁木齐市米东区≥50 岁居民骨密度与膳食模式的关系研究[J].职业与健康,2020,36(11):1522-1526.DOI:10.13329/j.cnki.zyyjk.2020.0404.
108	刘宇,刘敏,沈冲等.江苏省句容市农村地区 60 岁以上老年人骨质疏松流行情况及影响因素研究[J].实用老年医学,2020,34(06):580-584.
109	乔豆.河南农村成年人骨质疏松症患病率及影响因素[D].郑州大学,2020.DOI:10.27466/d.cnki.gzzdu.2020.004204.
110	桑乃华,刘菲,尹小兵.上海市静安区 1680 例老年人骨骼健康状况影响因素分析[J].实用预防医学,2020,27(07):874-876.
111	吴斌,董忠,李金龙.福州地区中老年人群骨质疏松症危险因素分析及中医证型与骨密度的关系[J].中医学,2020,9(6):468-474
112	姚立彬,张林华,李百战等.邢台山区老年人骨质疏松症流行病学调查及危险因素分析[J].解放军预防医学杂志,2020,38(02):58-60.DOI:10.13704/j.cnki.jyyx.2020.02.019.
113	张娅惠,薛秋艳,谷洁等.北京海淀部分人群骨质疏松流行病学及相关危险因素研究[J].现代生物医学进展,2020,20(13):2545-2550.DOI:10.13241/j.cnki.pmb.2020.13.031.
114	朱晓炜,王夏冬,李凯.张家港市中老年人群骨质疏松风险评估及危险因素调查[J].现代预防医学,2020,47(05):874-878.
115	宗欣,石秀娟,徐艳艳等.青岛市城阳区老年体检者骨质疏松症流行情况及相关因素调查[J].应用预防医学,2020,26(03):227-229+231.
116	曹志勇.1639 例老年人骨质疏松现状及相关因素分析[J].中华养生保健,2021,(6):15-16.
117	陈晓梅,祝进梅,李杰等.成都市 40 岁以上淡水养殖农户骨质疏松现状及相关因素研究[J].现代预防医学,2021,48(12):2175-2179+2188.
118	洪丽荣,谭晓霞,卢东辉等.深圳市体检人群骨质疏松症患病状况及其影响因素研究[J].中国医学创新,2021,18(13):78-82.
119	乔文婧.吉林省部分地区社区居民原发性骨质疏松症的患病情况及影响因素分析[D].吉林大学,2021.DOI:10.27162/d.cnki.gjlin.2021.004710.
120	沈静,杨蕾,李雪等.956 例中老年居民膳食模式与骨密度水平的相关研究[J].现代预防医学,2021,48(08):1391-1394.
121	孙晓明,邹祖琴,曹永蓓.南京市鼓楼区中老年女性人群骨密度现状及影响因素研究[J].华南预防医学,2021,47(05):583-587.
122	夏梦嘉,顾巧萍,赵晓燕.绝经后女性骨密度调查及骨质疏松危险因素分析[J].中国妇幼保健,2021,36(10):2331-2333.DOI:10.19829/j.zgfybj.issn.1001-4411.2021.10.045.
123	邢小丽,郑原媛,林秋萍.海口市绝经期女性骨质疏松情况及其影响因素[J].华南预防医学,2021,47(08):1069-1071+1075.

ID	Reference
124	杨弦弦,唐文革,汤成等.重庆市 40 岁及以上居民骨质疏松症流行现状及影响因素分析[J].中国慢性病预防与控制,2021,29(10):741-745.DOI:10.16386/j.cjpccd.issn.1004-6194.2021.10.005.
125	张文,郑伟,高磊等.乌鲁木齐地区老年骨质疏松患病状况及危险因素[J].中国老年学杂志,2021,41(14):2997-3000.
126	周柳娇,李吉,雷钧.中老年人群骨质疏松症影响因素分析[J].预防医学,2021,33(02):188-191.DOI:10.19485/j.cnki.issn2096-5087.2021.02.021.
127	任晓岚,李明阳,胡继宏等.甘肃省裕固族成年女性骨质疏松患病情况及影响因素分析[J].中国公共卫生,2022,38(02):235-240.
128	杨弦弦,丁贤彬,唐文革等.重庆市监测人群骨质疏松症患病率及其影响因素分析[J].公共卫生与预防医学,2022,33(01):90-94.
129	章铁立,魏戌,谢雁鸣,朱立国,高景华,申浩,柴言,孙梦华,张成,孙凯,唐彬,姜俊杰,支英杰,于忱忱.北京市社区中老年人群骨量评估及骨质疏松症检出率分析[J].中国骨伤,2020,33(10):916~92

Table S7. Quality scores for assessing the risk of bias in the included articles (n=129)

ID	Author	Year Published	Quality score					
			Sample population	Sample size	Participation	Outcome assessment	Analytical methods	Total scores
1	HL Shen, et al.	1996	2	1	2	2	1	8
2	J Tang, et al.	1997	1	1	2	2	2	8
3	EY Liao, et al.	2000	2	1	2	2	1	8
4	WZ Wang, et al.	2000	2	1	2	2	1	8
5	W Qing, et al.	2000	2	1	2	2	1	8
6	JG Yu, et al.	2000	2	1	2	2	1	8
7	JH Pan, et al.	2001	1	1	2	2	1	7
8	XD Yan, et al.	2001	1	1	2	2	1	7
9	NZ Zhou, et al.	2001	2	1	2	2	1	8
10	Z An, et al.	2002	2	1	2	2	1	8
11	YH Chen, et al.	2002	1	1	2	2	1	7
12	WZ Nie, et al.	2002	1	1	2	2	1	7
13	PF Wang, et al.	2002	2	1	2	2	1	8
14	R Wang, et al.	2002	1	1	2	2	1	7
15	W Lin, et al.	2003	2	1	2	2	1	8
16	Y Zhang, et al.	2004	2	1	2	2	1	8
17	YP Chen, et al.	2005	2	1	2	2	1	8
18	M Li	2005	1	1	2	2	2	8
19	QJ Zhou, et al.	2005	1	1	2	2	1	7
20	ZL Zhang, et al.	2006	2	1	2	2	1	8
21	YJ Meng, et al.	2006	1	1	2	2	1	7
22	Y Li, et al.	2007	2	1	2	2	1	8
23	BL Ma, et al.	2007	2	1	2	2	2	9
24	ML Pan, et al.	2007	1	1	2	2	1	7
25	Y Zhang, et al.	2007	2	1	2	2	2	9

ID	Author	Year Published	Quality score					
			Sample population	Sample size	Participation	Outcome assessment	Analytical methods	Total scores
26	P Zeng, et al.	2008	2	1	2	2	1	8
27	XG Chen, et al.	2008	2	1	2	2	2	9
28	PD Zhang, et al.	2008	1	1	2	2	1	7
29	WY Chen, et al.	2009	2	1	2	2	1	8
30	YJ Fu, et al.	2009	1	1	2	2	1	7
31	Ho SC, et al.	1999	1	1	2	2	1	7
32	Liao EY, et al.	2002	2	1	2	2	1	8
33	Wu XP, et al.	2004	2	1	2	2	1	8
34	Lynn HS, et al.	2005	1	1	2	2	2	8
35	Wu XP, et al.	2008	2	1	2	2	1	8
36	Lin YC, et al.	2011	1	1	0	2	2	6
37	WF Sun, et al.	2009	2	1	1	2	2	8
38	MX Wang, et al.	2010	2	1	2	2	1	8
39	HW Liu, et al.	2011	2	1	2	2	2	9
40	L Liu, et al.	2011	2	1	1	2	1	7
41	L Tao, et al.	2011	2	1	2	2	1	8
42	F Tian	2011	2	1	2	2	2	9
43	P Tu, et al.	2011	2	1	2	2	1	8
44	FT Wu,et al.	2011	2	1	2	2	2	9
45	F Sun, et al.	2012	1	1	2	1	2	7
46	CX Wang, et al.	2012	2	1	2	2	2	9
47	Y Yang, et al.	2012	2	1	2	2	2	9
48	R Li, et al.	2013	2	1	2	2	2	9
49	ZG Shen, et al.	2013	2	1	2	2	2	9
50	LY Wu,et al	2013	2	1	2	2	2	9
51	GZ Xu, et al.	2013	2	1	2	2	2	9
52	ZZ Zhang, et al.	2013	2	1	2	2	2	9

ID	Author	Year Published	Quality score					
			Sample population	Sample size	Participation	Outcome assessment	Analytical methods	Total scores
53	SS Lo, et al.	2015	1	1	0	2	2	6
54	CY Lee, et al.	2016	1	1	2	1	2	7
55	FK Zhang,et al.	2014	2	1	2	2	2	9
56	LQ Kou	2014	1	1	2	2	2	8
57	XW Gong,et al.	2014	1	1	2	2	2	8
58	XL Huang,et al.	2014	2	1	2	2	2	9
59	QY Luo,et al.	2014	2	1	2	2	2	9
60	J Tian,et al.	2014	1	1	2	2	2	8
61	DD Pang	2015	2	1	2	2	2	9
62	NN Kou	2016	2	1	2	2	2	9
63	XL Li	2016	2	2	2	2	2	10
64	YB Shi,et al.	2016	1	1	2	2	2	8
65	LF Zhao,et al.	2016	1	1	2	2	2	8
66	TY Yang,et al.	2016	2	1	2	2	2	9
67	WQ Fan,et al.	2016	1	1	2	2	2	8
68	Y Tang,et al.	2016	2	1	2	2	2	9
69	L Lin,et al.	2016	1	1	2	2	2	8
70	B Liu	2017	2	2	2	2	2	10
71	WX Qi	2017	2	1	2	2	2	9
72	RP Yang	2017	2	1	2	2	2	9
73	YQ Zhao	2017	2	1	2	2	2	9
74	XY Ding,et al.	2017	2	1	2	2	1	8
75	CZ Bing,et al.	2017	1	1	2	2	2	8
76	G Cao,et al.	2017	1	1	2	2	2	8
77	XX Tan,et al.	2017	2	1	0	2	2	7
78	LB Yao,et al.	2017	2	1	2	2	2	9
79	J Zhang,et al.	2017	1	1	2	2	1	7

ID	Author	Year Published	Quality score					
			Sample population	Sample size	Participation	Outcome assessment	Analytical methods	Total scores
80	SJ Tan,et al.	2017	1	1	2	2	2	8
81	HL Zhang,et al.	2017	2	1	2	2	2	9
82	YJ lin,et al.	2017	1	1	2	2	2	8
83	ZL Ye,et al.	2017	1	1	2	2	2	8
84	XR Wang,et al.	2017	2	1	2	2	2	9
85	Ko CH,et al.	2017	2	1	2	2	2	9
86	Zhang X,et al.	2018	2	1	2	2	2	9
87	J Chen,et al.	2018	2	1	2	2	2	9
88	T Chen,et al.	2018	2	1	2	2	2	9
89	ZH Fan,et al.	2018	2	1	2	2	1	8
90	RR Li,et al.	2018	2	1	2	2	2	9
91	Y Li	2018	2	1	2	2	2	9
92	Z Ma,et al.	2018	2	1	2	2	2	9
93	LM Tian,et al.	2018	2	1	2	2	2	9
94	XZ Wang,et al.	2018	2	1	2	2	2	9
95	JJ Xia, et al.	2018	1	1	2	2	2	8
96	LJ Yang, et al.	2018	2	1	2	2	1	8
97	QJ Zhu,et al.	2018	2	1	2	2	2	9
98	B Cai	2019	2	2	2	2	2	10
99	PQ Lai,et al.	2019	1	2	2	2	2	9
100	YY Luo,et al.	2019	1	1	2	2	2	8
101	DJ Wang，et al	2019	1	1	2	2	2	8
102	XQ Wen,et al.	2019	0	1	2	2	2	7
103	J Zeng,et al.	2020	1	1	2	2	2	8
104	QP Gu,et al.	2020	1	1	2	2	2	8
105	CH Lan,et al.	2020	0	1	2	2	2	7
106	JL Li	2020	1	2	2	2	2	9

ID	Author	Year Published	Quality score					
			Sample population	Sample size	Participation	Outcome assessment	Analytical methods	Total scores
107	X Li,et al.	2020	2	1	2	2	2	9
108	Y Liu,et al.	2020	2	1	2	2	2	9
109	D Qiao	2020	2	2	2	2	2	10
110	NH Sang,et al.	2020	2	2	2	2	2	10
111	B Wu,et al.	2020	1	1	2	2	2	8
112	LB Yao,et al.	2020	2	1	2	2	2	9
113	YH Zhang,et al.	2020	1	1	2	2	2	8
114	XW Zhu,et al.	2020	2	2	2	2	2	10
115	X Zong,et al.	2020	1	1	2	2	2	8
116	ZY Cao,et al.	2021	1	1	2	2	1	7
117	XM Chen,et al.	2021	2	2	2	2	2	10
118	LR Hong	2021	1	1	2	2	2	8
119	WJ Qiao	2021	2	1	2	2	2	9
120	J Shen,et al.	2021	1	1	2	2	2	8
121	XM Sun,et al.	2021	0	1	2	2	2	7
122	MJ Xia,et al.	2021	1	1	2	2	2	8
123	XL Xing,et al.	2021	1	1	2	2	2	8
124	XX Yang,et al.	2021	1	1	2	2	2	8
125	W Zhang,et al.	2021	2	1	2	2	2	9
126	HJ Zhou,et al.	2021	1	1	2	2	2	8
127	XL Ren,et al.	2021	2	1	2	2	2	9
128	XX Yang,et al.	2021	1	1	2	2	2	8
129	YL Zhang,et al.	2020	2	1	2	2	1	8

Table S8. Characteristics of included article

ID	Author	Year Published	Province	Study setting	Investi-gation year	Defini-tions	Method of examination	Bones	Age range (years)	Tested sample	Cases	Included in prevalence analysis	Included in associated factors analysis
1	HL Shen, et al.	1996	Beijing	Mixed	1994	WHO	DXA	Lumbar spine	60-93	625	235	Yes	No
2	J Tang, et al.	1997	Anhui	Mixed	NR	Other	Spa-4 Bone Mineral Analyzer	Mixed	>0	23572	3610	No	Yes
3	EY Liao, et al.	2000	Hunan	Mixed	1997	WHO	DXA	Lumbar spine	30-96	1518	227	Yes	No
								Femoral neck		1518	121		
								Ward		1518	331		
4	WZ Wang, et al.	2000	Sichuan	Mixed	1997	CHN	DXA	Femoral neck	>40	1196	647	Yes	No
								Ward	>40	1196	669		
5	W Qing, et al.	2000	Beijing	Mixed	1997	CHN	DXA	Femoral neck	60-94	436	248	Yes	No
								Ward	60-94	436	231		
6	JG Yu, et al.	2000	Shannxi	Mixed	1997	WHO	DXA	Lumbar spine	42-72	300	123	Yes	No
7	JH Pan, et al.	2001	Mixed	Mixed	1998	WHO	DXA	Ward	50-85	3039	937	Yes	No
8	XD Yan, et al.	2001	Guangxi	Mixed	1998	CHN	DXA	Femoral neck	46-72	142	2	Yes	No
								Ward	46-72	142	10		
9	NZ Zhou, et al.	2001	Fujian	Mixed	1998	CHN	DXA	Lumbar spine	60-70	182	33	Yes	No
10	Z An, et al.	2002	Sichuan	Mixed	1999	CHN	DXA	Femoral neck	>40	1240	479	Yes	No
								Ward	>40	1240	689		
11	YH Chen, et al.	2002	Guangxi	Mixed	1999	CHN	DXA	Femoral neck	40-71	128	6	Yes	No
								Ward	40-71	128	16		
12	WZ Nie, et al.	2002	Shanghai	Mixed	1999	CHN	DXA	Ward	40-85	401	252	Yes	No

ID	Author	Year Published	Province	Study setting	Investi-gation year	Defini-tions	Method of examination	Bones	Age range (years)	Tested sample	Cases	Included in prevalence analysis	Included in associated factors analysis
13	PF Wang, et al.	2002	Hunan	Rural	1999	WHO	DXA	Lumbar spine	40-84	627	108	Yes	No
								Femoral neck		627	48		
								Ward		627	131		
14	R Wang, et al.	2002	Henan	Mixed	1999	CHN	DXA	Lumbar spine	19-73	338	75	Yes	No
15	W Lin, et al.	2003	Guangdong	Mixed	1998	WHO	DXA	Ward	40-89	1197	394	Yes	No
16	Y Zhang, et al.	2004	Beijing	Urban	NR	WHO	DXA	Femoral neck	24-85	317	42	Yes	No
								Ward		317	25		
17	YP Chen, et al.	2005	Guangdong	Mixed	2003	CHN	DXA	Femoral neck	50-97	133	25	Yes	No
18	M Li	2005	Shanghai	Urban	NR	WHO	pDXA	Mixed	40-90	1840	245	No	Yes
19	QJ Zhou, et al.	2005	Yunnan	Mixed	2002	CHN	DXA	Femoral neck	50-90	157	49	Yes	No
								Ward	50-90	157	103		
20	ZL Zhang, et al.	2006	Shanghai	Mixed	NR	WHO	DXA	Lumbar spine	50-89	1084	59	Yes	No
								Femoral neck		1084	68		
21	YJ Meng, et al.	2006	Guangxi	Mixed	2003	CHN	DXA	Femoral neck	40-88	1230	572	Yes	No
22	Y Li, et al.	2007	Heilongjiang	Mixed	NR	WHO	DXA	Lumbar spine	46-70	155	12	Yes	No
23	BL Ma, et al.	2007	Shanghai	Mixed	NR	WHO	DXA	Lumbar spine	40-70	319	67	Yes	No
24	ML Pan, et al.	2007	Guangxi	Mixed	2003	CHN	DXA	Femoral neck	20-87	384	116	Yes	No
								Ward	20-87	384	193		
25	Y Zhang, et al.	2007	Beijing	Mixed	NR	WHO	DXA	Femoral neck	40-85	308	106	Yes	No

ID	Author	Year Published	Province	Study setting	Investi-gation year	Defini-tions	Method of examination	Bones	Age range (years)	Tested sample	Cases	Included in prevalence analysis	Included in associated factors analysis
								Ward		308	129		
26	P Zeng, et al.	2008	Beijing	Mixed	1998	WHO	DXA	Femoral neck	45-85	289	41	Yes	No
								Ward		289	25		
27	XG Chen, et al.	2008	Mixed	Mixed	NR	WHO	DXA	Lumbar spine	50-89	5083	1437	Yes	No
28	PD Zhang, et al.	2008	Guangdong	Mixed	2007	WHO	DXA	Lumbar spine	40-84	839	309	No	Yes
								Ward		414	64		
29	WY Chen, et al.	2009	Hainan	Mixed	NR	WHO	DXA	Femoral neck	>40	1010	348	Yes	No
								Ward		1010	396		
30	YJ Fu, et al.	2009	Henan	Mixed	2006	CHN	DXA	Lumbar spine	40-89	727	238	Yes	No
31	Ho SC, et al.	1999	Hongkong	Mixed	NR	WHO	DXA	Femoral neck	>41	480	160	Yes	No
								Ward		480	234		
								Lumbar spine		2255	428		
32	Liao EY, et al.	2002	Hunan	Mixed	NR	WHO	DXA	Femoral neck	30-96	2255	203	Yes	No
								Ward		2255	583		
						WHO		Lumbar spine	30-90	2553	397		
33	Wu XP, et al.	2004	Hunan	Mixed	1999		DXA	Femoral neck		2553	200	Yes	No
						CHN		Lumbar spine	30-90	2553	530		
34	Lynn HS, et al.	2005	Hongkong	Mixed	NR	WHO	DXA	Lumbar spine	40-94	3217	1204	Yes	No
35	Wu XP, et al.	2008	Hunan	Mixed	2003	CHN	DXA	Femoral neck	50-85	766	212	Yes	No

ID	Author	Year Published	Province	Study setting	Investi-gation year	Defini-tions	Method of examination	Bones	Age range (years)	Tested sample	Cases	Included in prevalence analysis	Included in associated factors analysis
								Ward	50-85	766	562		
36	Lin YC, et al.	2011	Taiwan	Mixed	2007	WHO	DXA	Lumbar spine	>50	649	63	Yes	No
								Femoral neck		649	122		
37	WF Sun, et al.	2009	Zhejiang	Urban	NR	Other	DXA	Mixed	54-82	186	67	No	Yes
38	MX Wang, et al.	2010	Jiangsu	Mixed	2007	WHO	DXA	Femoral neck	15-89	1572	393	Yes	No
								Ward		1572	493		
39	HW Liu, et al.	2011	Hunan	Mixed	2009	WHO	DXA	Femoral neck	25-87	4000	92	Yes	No
								Ward		4000	208		
40	L Liu, et al.	2011	Guizhou	Urban	2010	WHO	DXA	Lumbar spine	20-75	1215	103	Yes	No
								Femoral neck		1215	30		
41	L Tao, et al.	2011	Beijing	Urban	NR	WHO	DXA	Femoral neck	60-86	415	12	Yes	No
42	F Tian	2011	mixed	Mixed	2009	Other	DXA	Mixed	40-65	1740	397	No	Yes
43	P Tu, et al.	2011	Jiangxi	Urban	NR	WHO	DXA	Lumbar spine	20-89	576	127	Yes	No
								Femoral neck		576	9		
44	FT Wu,et al.	2011	Anhui	Urban	NR	WHO	Ultrasound Bone Sonometers	Mixed	>40	820	126	No	Yes
45	F Sun, et al.	2012	Taiwan	Mixed	2002	Other	DXA	Mixed	35-74	7801	505	No	Yes
46	CX Wang, et al.	2012	Jiangxi	Urban	2011	WHO	Ultrasound Bone Sonometers	Mixed	>40	5999	648	No	Yes
47	Y Yang, et al.	2012	Beijing	Urban	2008	CHN	DXA	Lumbar spine	40-83	518	171	Yes	No
								Femoral neck	40-83	518	175		

ID	Author	Year Published	Province	Study setting	Investi-gation year	Defini-tions	Method of examination	Bones	Age range (years)	Tested sample	Cases	Included in prevalence analysis	Included in associated factors analysis
48	R Li, et al.	2013	Beijing	Mixed	NR	WHO	DXA	Lumbar spine	49.08±6.012	306	34	No	Yes
49	LY Ruan,et al.	2013	Zhejiang	Urban	NR	Other	Ultrasound Bone Sonometers	Mixed	45-75	912	313	No	Yes
50	LY Wu,et al	2013	Hubei	Mixed	NR	WHO	DXA	Lumbar spine	>60	183	126	Yes	Yes
51	GZ Xu, et al.	2013	Shanghai	Mixed	2012	WHO	Ultrasound Bone Sonometers	Mixed	>65	1504	352	No	Yes
52	ZZ Zhang, et al.	2013	Anhui	Mixed	2011	WHO	DXA	Mixed	40-65	804	210	No	Yes
53	SS Lo, et al.	2015	Hongkong	Mixed	2010	WHO	DXA	Lumbar spine	58.2 ± 6.4	1507	383	No	Yes
54	CY Lee, et al.	2016	Taiwan	Mixed	2010	WHO	DXA	NR	>65	4360	1499	No	Yes
55	FK Zhang,et al.	2014	Anhui	Urban	NR	WHO	Ultrasound Bone Sonometers	Mixed	>40	820	126	No	Yes
56	LQ Kou	2014	Sichuan	Urban	2011	WHO	DXA	Mixed	>60	276	137	No	Yes
57	XW Gong,et al.	2014	Heilongjiang	Mixed	2011	WHO	Ultrasound Bone Sonometers	Mixed	NR	500	107	No	Yes
58	XL Huang,et al.	2014	Guangdong	Urban	2013	WHO	DXA	Mixed	61-85	2285	185	No	Yes
59	QY Luo,et al.	2014	Sichuan	Mixed	2013	WHO	DXA	Mixed	30-90	373	204	No	Yes
60	J Tian,et al.	2014	Inner Mongolia	Mixed	2013	WHO	Ultrasound Bone Sonometers	Mixed	27-74	300	43	No	Yes
61	DD Pang	2015	Jilin	Mixed	2013	WHO	Ultrasound Bone Sonometers	Mixed	21-88	1331	82	No	Yes
62	NN Kou	2016	Yunnan	Rural	2015	WHO	Ultrasound Bone Sonometers	Mixed	20-79	160	23	No	Yes
63	XL Li	2016	Guangdong	Mixed	2016	WHO	Ultrasound Bone Sonometers	Mixed	40-93	483	188	No	Yes
64	YB Shi,et al.	2016	Shanghai	Mixed	2014	WHO	Ultrasound Bone Sonometers	Mixed	>65	594	360	No	Yes
65	LF Zhao,et al.	2016	Zhejiang	Mixed	2014	WHO	DXA	Mixed	40-80	368	82	No	Yes
66	TY Yang,et al.	2016	Guizhou	Rural	2014	WHO	Ultrasound Bone Sonometers	Mixed	20-75	19973	3392	No	Yes

ID	Author	Year Published	Province	Study setting	Investi-gation year	Defini-tions	Method of examination	Bones	Age range (years)	Tested sample	Cases	Included in prevalence analysis	Included in associated factors analysis
67	WQ Fan,et al.	2016	Henan	Mixed	2014	WHO	Ultrasound Bone Sonometers	Mixed	31-82	4280	501	No	Yes
68	Y Tang,et al.	2016	Sichuan	Mixed	NR	WHO	DXA	Lumbar spine	30-90	376	119	Yes	No
69	L Lin,et al.	2016	Jiangsu	Mixed	NR	WHO	Ultrasound Bone Sonometers	Mixed	40-69	1803	231	No	Yes
70	B Liu	2017	Gansu	Mixed	2016	WHO	Ultrasound Bone Sonometers	Mixed	20-80	5857	746	No	Yes
71	WX Qi	2017	Xinjiang	Mixed	2015	Other	Ultrasound Bone Sonometers	Mixed	>18	748	52	No	Yes
72	RP Yang	2017	Gansu	Mixed	NR	WHO	DXA	Mixed	18-79	12085	638	No	Yes
73	YQ Zhao	2017	Shanxi	Mixed	NR	WHO	DXA	NR	20-87	529	64	No	Yes
74	XY Ding,et al.	2017	Chongqing	Urban	2014	WHO	DXA	Femoral neck	>40	813	136	Yes	No
								Ward		813	160		
75	CZ Bing,et al.	2017	Ningxia	Mixed	2015	WHO	DXA	Lumbar spine	NR	1925	122	No	Yes
76	G Cao,et al.	2017	Ningxia	Mixed	2015	WHO	DXA	Mixed	40-59	1000	84	No	Yes
77	XX Tan,et al.	2017	Guangdong	Mixed	2016	WHO	Ultrasound Bone Sonometers	Mixed	>40	182	86	No	Yes
78	LB Yao,et al.	2017	Hebei	Rural	2015	Other	DXA	Mixed	60-90	8159	1324	No	Yes
79	J Zhang,et al.	2017	Shandong	Urban	NR	WHO	Ultrasound Bone Sonometers	Mixed	>20	507	87	No	Yes
80	SJ Tan,et al.	2017	Guangxi	Mixed	2016	WHO	DXA	Mixed	40-79	216	84	No	Yes
81	HL Zhang,et al.	2017	Hainan	Rural	2015	WHO	DXA	Mixed	65-92	2186	691	No	Yes
82	YJ lin,et al.	2017	Henan	Mixed	2016	WHO	DXA	Mixed	49-85	120	51	No	Yes
83	ZL Ye,et al.	2017	Hainan	Mixed	2014	WHO	DXA	Mixed	20-79	7286	1413	No	Yes
84	XR Wang,et al.	2017	Beijing	Mixed	2013	WHO	DXA	Mixed	>40	380	107	No	Yes

ID	Author	Year Published	Province	Study setting	Investi-gation year	Defini-tions	Method of examination	Bones	Age range (years)	Tested sample	Cases	Included in prevalence analysis	Included in associated factors analysis
85	Ko CH,et al.	2017	Taiwan	Mixed	2010	WHO	DXA	Mixed	>50	3734	362	No	Yes
86	Zhang X,et al.	2018	Beijing	Mixed	2015	WHO	DXA	Lumbar spine	50-90	1349	146	Yes	No
								Femoral neck		1349	59		
87	J Chen,et al.	2018	Yunnan	Mixed	2016	WHO	Ultrasound Bone Sonometers	Mixed	>20	9809	394	No	Yes
88	T Chen,et al.	2018	Chongqing	Urban	2016	WHO	Ultrasound Bone Sonometers	Mixed	>40	2028	127	No	Yes
89	ZH Fan,et al.	2018	Beijing	Urban	NR	WHO	DXA	Lumbar spine	50-90	1349	143	Yes	No
90	RR Li,et al.	2018	Heilongjiang	Rural	NR	WHO	DXA	Mixed	>60	1877	142	No	Yes
91	Y Li	2018	Guangxi	Mixed	NR	WHO	Ultrasound Bone Sonometers	Mixed	18-89	1225	189	No	Yes
92	Z Ma,et al.	2018	Fujian	Mixed	2016	WHO	DXA	Mixed	>45	1290	480	No	Yes
93	LM Tian,et al.	2018	Gansu	Mixed	2014	WHO	DXA	Mixed	>38	6564	583	No	Yes
94	XZ Wang,et al.	2018	Zhejiang	Mixed	2016	WHO	X-ray Bone Densitometer	Mixed	>60	354	138	No	Yes
95	JJ Xia, et al.	2018	Sichuan	Urban	NR	WHO	Ultrasound Bone Sonometers	Mixed	40-89	1389	406	No	Yes
96	LJ Yang, et al.	2018	Guangdong	Urban	2015	CHN	DXA	Lumbar spine	40-89	1529	469	Yes	No
97	QJ Zhu,et al.	2018	Henan	Mixed	2015	WHO	DXA	Mixed	31-56	560	86	No	Yes
98	B Cai	2019	Shanxi	Urban	NR	WHO	DXA	Lumbar spine	>50	260	74	Yes	No
								Femoral neck		260	79		
99	PQ Lai,et al.	2019	Guangdong	Urban	NR	WHO	DXA	Lumbar spine	45-70	746	274	Yes	Yes
100	YY Luo,et al.	2019	Sichuan	Urban	2018	WHO	DXA	Mixed	>20	9342	1856	No	Yes
101	DJ Wang，et al	2019	Shandong	Mixed	2018	WHO	DXA	Mixed	26-58	352	30	No	Yes

ID	Author	Year Published	Province	Study setting	Investi-gation year	Defini-tions	Method of examination	Bones	Age range (years)	Tested sample	Cases	Included in prevalence analysis	Included in associated factors analysis
102	XQ Wen,et al.	2019	Shanxi	Mixed	2018	WHO	DXA	Mixed	40-60	484	95	No	Yes
103	J Zeng,et al.	2020	Hunan	Urban	2019	WHO	DXA	Femoral neck	>40	1245	357	No	Yes
104	QP Gu,et al.	2020	Zhejiang	Mixed	2019	WHO	DXA	Mixed	58-83	1562	535	No	Yes
105	CH Lan,et al.	2020	Guangdong	Mixed	NR	WHO	Ultrasound Bone Sonometers	NR	>40	2195	457	No	Yes
106	JL Li	2020	Fujian	Urban	NR	WHO	DXA	Mixed	50-70	260	84	No	Yes
107	X Li,et al.	2020	Xinjiang	Urban	2018	WHO	Ultrasound Bone Sonometers	Mixed	>50	502	104	No	Yes
108	Y Liu,et al.	2020	Jiangsu	Rural	2015	WHO	Ultrasound Bone Sonometers	Mixed	>60	5987	805	No	Yes
109	D Qiao	2020	Henan	Rural	2016	WHO	Ultrasound Bone Sonometers	Mixed	18-79	8033	1636	No	Yes
110	NH Sang,et al.	2020	Shanghai	Urban	NR	Other	X-ray Bone Densitometer	Mixed	65-96	1680	501	No	Yes
111	B Wu,et al.	2020	Fujian	Urban	2018	WHO	DXA	Mixed	58.80 ± 5.33	464	120	No	Yes
112	LB Yao,et al.	2020	Hebei	Rural	2018	WHO	Ultrasound Bone Sonometers	Mixed	>65	2413	870	No	Yes
113	YH Zhang,et al.	2020	Beijing	Urban	2018	WHO	DXA	Mixed	24-88	346	93	No	Yes
114	XW Zhu,et al.	2020	Jiangsu	Mixed	2019	WHO	Ultrasound Bone Sonometers	Mixed	40-79	3138	1157	No	Yes
115	X Zong,et al.	2020	Shandong	Urban	2016	WHO	DXA	Mixed	60-89	1180	359	No	Yes
116	ZY Cao,et al.	2021	Hebei	Mixed	2018	WHO	DXA	Mixed	>60	1639	524	No	Yes
117	XM Chen,et al.	2021	Sichuan	Rural	2019	WHO	DXA	Mixed	>40	620	155	No	Yes
118	LR Hong	2021	Guangdong	Rural	2019	WHO	DXA	Mixed	20-91	16382	1156	No	Yes
119	WJ Qiao	2021	Jilin	Mixed	2017	WHO	DXA	Mixed	>40	1517	318	No	Yes
120	J Shen,et al.	2021	Xinjiang	Urban	2018	WHO	Ultrasound Bone Sonometers	Mixed	45-87	956	207	No	Yes

ID	Author	Year Published	Province	Study setting	Investi-gation year	Defini-tions	Method of examination	Bones	Age range (years)	Tested sample	Cases	Included in prevalence analysis	Included in associated factors analysis
121	XM Sun,et al.	2021	Jiangsu	Mixed	2020	WHO	DXA	Mixed	45-75	8550	1303	No	Yes
122	MJ Xia,et al.	2021	Zhejiang	Mixed	2020	WHO	DXA	Mixed	50-70	220	90	No	Yes
123	XL Xing,et al.	2021	Hainan	Mixed	2020	Other	DXA	Mixed	45-77	2123	722	No	Yes
124	XX Yang,et al.	2021	Chongqing	Mixed	2018	WHO	DXA	Mixed	>40	1599	240	No	Yes
125	W Zhang,et al.	2021	Xinjiang	Urban	2018	Other	Ultrasound Bone Sonometers	Mixed	>60	7391	3154	No	Yes
126	HJ Zhou,et al.	2021	Zhejiang	Mixed	2019	WHO	DXA	Mixed	>45	482	254	No	Yes
127	XL Ren,et al.	2021	Gansu	Mixed	2016	Other	Ultrasound Bone Sonometers	Mixed	20-80	929	130	No	Yes
128	XX Yang,et al.	2021	Chongqing	Mixed	2018	WHO	DXA	Mixed	20-87	1855	241	No	Yes
129	YL Zhang,et al.	2020	Beijing	Urban	2018	WHO	DXA	Lumbar spine	45-80	1520	424	Yes	No

Notes: WHO: T-score of <-2.5 SD, CHN: T-score of <-2.0 SD, other: Not WHO criteria or CHN criteria, DXA: Dual-energy X-ray Absorptiometry, pDXA: peripheral Dual Energy X-ray Absorptiometry, NR: n.

Table S9. Estimated age- and sex-specific prevalence of osteoporosis in China in 2020

Age (years)	Prevalence (%, 95%CI)								
	Male			Female			Overall		
	Lumbar spine	Femoral neck	Ward	Lumbar spine	Femoral neck	Ward	Lumbar spine	Femoral neck	Ward
**WHO Criteria**									
**20-24 years**	4.75 (2.90-7.71)	0.68 (0.29-1.62)	0.62 (0.28-1.39)	2.18 (1.65-2.88)	0.12 (0.06-0.22)	0.16 (0.10-0.24)	3.53 (2.30-5.42)	0.41 (0.18-0.95)	0.40 (0.19-0.85)
**25-29 years**	5.31 (3.35-8.33)	0.94 (0.41-2.14)	0.93 (0.42-2.03)	3.10 (2.37-4.05)	0.21 (0.12-0.39)	0.33 (0.22-0.49)	4.26 (2.88-6.29)	0.59 (0.27-1.30)	0.64 (0.32-1.30)
**30-34 years**	5.94 (3.86-9.02)	1.29 (0.58-2.84)	1.38 (0.64-2.95)	4.40 (3.41-5.67)	0.39 (0.22-0.70)	0.68 (0.46-1.01)	5.19 (3.64-7.39)	0.85 (0.41-1.80)	1.04 (0.55-2.00)
**35-39 years**	6.63 (4.45-9.77)	1.77 (0.82-3.76)	2.04 (0.96-4.27)	6.21 (4.87-7.89)	0.72 (0.41-1.26)	1.41 (0.96-2.05)	6.42 (4.65-8.86)	1.26 (0.62-2.55)	1.73 (0.96-3.19)
**40-44 years**	7.39 (5.10-10.60)	2.42 (1.16-5.00)	3.00 (1.44-6.17)	8.69 (6.90-10.89)	1.32 (0.76-2.27)	2.89 (2.01-4.12)	8.02 (5.98-10.74)	1.89 (0.97-3.67)	2.95 (1.72-5.17)
**45-49 years**	8.23 (5.82-11.54)	3.31 (1.62-6.63)	4.41 (2.15-8.84)	12.04 (9.68-14.87)	2.40 (1.41-4.05)	5.83 (4.14-8.15)	10.10 (7.71-13.17)	2.86 (1.52-5.37)	5.11 (3.13-8.50)
**50-54 years**	9.17 (6.61-12.58)	4.51 (2.26-8.79)	6.43 (3.19-12.55)	16.44 (13.40-20.00)	4.33 (2.59-7.16)	11.42 (8.31-15.50)	12.77 (9.97-16.26)	4.42 (2.42-7.98)	8.91 (5.73-14.02)
**55-59 years**	10.19 (7.46-13.77)	6.11 (3.12-11.61)	9.29 (4.70-17.56)	22.05 (18.24-26.39)	7.69 (4.69-12.35)	21.18 (15.95-27.57)	16.11 (12.84-20.07)	6.90 (3.90-11.98)	15.23 (10.31-22.55)
**60-64 years**	11.31 (8.37-15.12)	8.23 (4.28-15.26)	13.24 (6.85-24.05)	28.90 (24.31-33.98)	13.29 (8.33-20.54)	35.90 (28.36-44.20)	20.06 (16.3-24.5)	10.75 (6.29-17.88)	24.51 (17.55-34.07)
**65-69 years**	12.55 (9.34-16.65)	11.00 (5.81-19.87)	18.53 (9.87-32.07)	36.89 (31.57-42.54)	22.00 (14.35-32.21)	53.85 (45.16-62.31)	24.93 (20.65-29.82)	16.60 (10.15-26.14)	36.49 (27.82-47.45)
**70-74 years**	13.89 (10.35-18.40)	14.56 (7.80-25.55)	25.31 (14.00-41.37)	45.66 (39.83-51.61)	34.18 (23.54-46.70)	70.85 (63.06-77.59)	30.14 (25.43-35.39)	24.60 (15.85-36.37)	48.61 (39.10-59.90)
**75-79 years**	15.35 (11.39-20.37)	19.02 (10.36-32.32)	33.55 (19.43-51.40)	54.71 (48.67-60.61)	48.87 (36.06-61.83)	83.51 (77.93-87.91)	36.08 (31.03-41.57)	34.74 (23.90-47.86)	59.87 (50.24-70.63)
**80-84 years**	16.94 (12.47-22.59)	24.45 (13.57-40.03)	42.93 (26.27-61.36)	63.46 (57.56-68.98)	63.76 (50.76-75.02)	91.35 (87.92-93.87)	42.55 (37.30-48.14)	46.10 (34.05-59.30)	69.59 (60.22-79.26)
**85-89 years**	18.65 (13.58-25.07)	30.85 (17.51-48.39)	52.85 (34.45-70.51)	71.40 (65.95-76.29)	76.40 (65.26-84.80)	95.65 (93.74-97.00)	49.89 (44.60-55.41)	57.83 (45.79-69.96)	78.20 (69.56-86.20)
**Total**	**8.63 (6.10-12.12)**	**5.11 (2.65-9.48)**	**7.96 (4.28-13.90)**	**17.75 (15.01-20.82)**	**9.37 (6.54-12.97)**	**19.26 (16.37-22.43)**	**13.14 (10.50-16.42)**	**7.21 (4.57-11.20)**	**13.54 (10.25-18.11)**

**CHN Criteria**									
**20-24 years**	2.71 (1.11-6.48)	5.43 (3.50-8.35)	9.85 (3.80-23.17)	1.96 (1.21-3.17)	1.16 (0.41-3.24)	0.61 (0.26-1.46)	2.36 (1.15-4.91)	3.40 (2.03-5.93)	5.47 (2.12-12.87)
**25-29 years**	3.45 (1.56-7.45)	6.96 (4.66-10.28)	12.64 (5.09-28.07)	3.02 (1.91-4.76)	1.83 (0.66-4.99)	1.16 (0.50-2.64)	3.25 (1.73-6.16)	4.51 (2.74-7.75)	7.15 (2.90-15.92)
**30-34 years**	4.39 (2.20-8.56)	8.87 (6.16-12.60)	16.08 (6.76-33.62)	4.63 (2.99-7.09)	2.89 (1.06-7.64)	2.17 (0.98-4.76)	4.50 (2.59-7.85)	5.96 (3.68-10.19)	9.32 (3.95-19.59)
**35-39 years**	5.56 (3.08-9.83)	11.24 (8.11-15.39)	20.25 (8.92-39.71)	7.02 (4.66-10.45)	4.53 (1.70-11.54)	4.05 (1.89-8.45)	6.27 (3.85-10.13)	7.98 (4.99-13.52)	12.38 (5.50-24.53)
**40-44 years**	7.02 (4.30-11.28)	14.15 (10.56-18.71)	25.17 (11.64-46.21)	10.53 (7.17-15.19)	7.03 (2.70-17.08)	7.42 (3.62-14.63)	8.73 (5.70-13.18)	10.68 (6.73-17.91)	16.52 (7.73-30.81)
**45-49 years**	8.84 (5.95-12.93)	17.67 (13.61-22.61)	30.83 (15.02-52.92)	15.48 (10.85-21.61)	10.76 (4.27-24.58)	13.21 (6.76-24.22)	12.10 (8.35-17.19)	14.28 (9.03-23.58)	22.19 (10.97-38.84)
**50-54 years**	11.06 (8.15-14.84)	21.83 (17.30-27.15)	37.12 (19.12-59.59)	22.19 (16.05-29.86)	16.13 (6.68-34.08)	22.43 (12.24-37.48)	16.58 (12.06-22.29)	19.01 (12.04-30.59)	29.84 (15.71-48.63)
**55-59 years**	13.76 (10.98-17.12)	26.66 (21.65-32.34)	43.89 (23.96-66.00)	30.76 (23.02-39.75)	23.47 (10.27-45.11)	35.46 (21.06-53.08)	22.24 (16.99-28.41)	25.07 (15.97-38.71)	39.68 (22.52-59.55)
**60-64 years**	17.00 (14.38-19.98)	32.11 (26.61-38.16)	50.89 (29.52-71.94)	40.89 (31.79-50.65)	32.84 (15.45-56.69)	51.07 (33.65-68.22)	28.88 (23.04-35.23)	32.48 (21.06-47.39)	50.98 (31.58-70.09)
**65-69 years**	20.81 (17.99-23.94)	38.11 (32.09-44.51)	57.86 (35.69-77.25)	51.85 (42.00-61.56)	43.81 (22.54-67.63)	66.47 (48.94-80.39)	36.60 (30.20-43.08)	41.01 (27.23-56.27)	62.24 (42.43-78.85)
**70-74 years**	25.22 (21.37-29.49)	44.48 (37.94-51.23)	64.52 (42.29-81.86)	62.64 (52.85-71.50)	55.42 (31.63-76.97)	79.02 (64.29-88.73)	44.37 (37.48-50.99)	50.08 (34.71-64.40)	71.94 (53.55-85.38)
**75-79 years**	30.20 (24.56-36.51)	51.05 (44.00-58.06)	70.67 (49.10-85.75)	72.31 (63.35-79.77)	66.47 (42.32-84.27)	87.73 (77.08-93.83)	52.38 (45.00-59.30)	59.17 (43.12-71.87)	79.66 (63.84-90.01)
**80-84 years**	35.70 (27.75-44.52)	57.58 (50.09-64.73)	76.15 (55.87-88.95)	80.26 (72.66-86.15)	75.97 (53.74-89.59)	93.14 (86.21-96.72)	60.24 (52.48-67.44)	67.71 (52.10-78.42)	85.51 (72.58-93.23)
**85-89 years**	41.60 (31.04-53.00)	63.85 (56.07-70.98)	80.88 (62.36-91.52)	86.36 (80.30-90.77)	83.45 (64.72-93.27)	96.27 (92.04-98.29)	68.11 (60.21-75.37)	75.46 (61.19-84.18)	89.99 (79.94-95.53)
**Total**	**10.87 (8.08-14.94)**	**20.44 (16.35-25.29)**	**32.97 (18.11-52.08)**	**23.47 (18.51-29.16)**	**19.02 (9.93-32.10)**	**25.92 (18.47-34.72)**	**17.10 (13.23-21.96)**	**19.74 (13.18-28.65)**	**29.49 (18.29-43.50)**

Notes: WHO: T-score of <-2.5 SD, CHN: T-score of <-2.0 SD.

Table S10. Estimated age- and sex-specific number of cases of osteoporosis in China in 2020

Age (years)	Cases (million, 95%CI)								
	**Male**			**Female**			**Overall**		
	**Lumbar spine**	**Femoral neck**	**Ward**	**Lumbar spine**	**Femoral neck**	**Ward**	**Lumbar spine**	**Femoral neck**	**Ward**
**WHO Criteria**									
**20-24 years**	2.00	0.29	0.26	0.83	0.04	0.06	2.83	0.33	0.32
	(1.22-3.25)	(0.12-0.68)	(0.12-0.59)	(0.63-1.09)	(0.02-0.08)	(0.04-0.09)	（1.84-4.34）	（0.14-0.76）	（0.16-0.68）
**25-29 years**	2.57	0.46	0.45	1.38	0.09	0.15	3.95	0.55	0.59
	(1.62-4.03)	(0.20-1.04)	(0.20-0.98)	(1.05-1.79)	(0.05-0.17)	(0.10-0.22)	（2.67-5.83）	（0.25-1.21）	（0.30-1.20）
**30-34 years**	3.75	0.82	0.87	2.63	0.23	0.41	6.38	1.05	1.27
	(2.44-5.69)	(0.37-1.79)	(0.40-1.86)	(2.04-3.39)	(0.13-0.42)	(0.27-0.60)	（4.48-9.08）	（0.50-2.21）	（0.68-2.46）
**35-39 years**	3.47	0.93	1.07	3.08	0.36	0.70	6.55	1.29	1.76
	(2.33-5.12)	(0.43-1.97)	(0.50-2.24)	(2.41-3.91)	(0.20-0.63)	(0.48-1.01)	（4.74-9.03）	（0.63-2.60）	（0.98-3.25）
**40-44 years**	3.65	1.20	1.48	4.08	0.62	1.35	7.72	1.81	2.84
	(2.51-5.23)	(0.57-2.47)	(0.71-3.04)	(3.24-5.11)	(0.36-1.07)	(0.94-1.94)	（5.75-10.34）	（0.93-3.53）	（1.65-4.98）
**45-49 years**	4.77	1.92	2.56	6.71	1.34	3.25	11.48	3.26	5.81
	(3.37-6.68)	(0.94-3.84)	(1.24-5.12)	(5.40-8.30)	(0.79-2.26)	(2.31-4.55)	（8.77-14.98）	（1.73-6.11）	（3.55-9.67）
**50-54 years**	5.40	2.66	3.79	9.52	2.51	6.62	14.93	5.17	10.41
	(3.89-7.42)	(1.33-5.18)	(1.88-7.40)	(7.76-11.59)	(1.50-4.15)	(4.82-8.98)	（11.66-19.01）	（2.83-9.33）	（6.70-16.38）
**55-59 years**	5.05	3.03	4.60	10.87	3.79	10.45	15.92	6.82	15.05
	(3.69-6.82)	(1.55-5.75)	(2.33-8.69)	(9.00-13.02)	(2.31-6.09)	(7.87-13.60)	（12.69-19.83）	（3.86-11.84）	（10.19-22.29）
**60-64 years**	4.12	3.00	4.82	10.40	4.78	12.92	14.52	7.78	17.74
	(3.05-5.50)	(1.56-5.55)	(2.49-8.75)	(8.75-12.23)	(3.00-7.39)	(10.21-15.91)	（11.8-17.73）	（4.56-12.94）	（12.70-24.66）
**65-69 years**	4.44	3.89	6.55	13.50	8.05	19.71	17.94	11.94	26.26
	(3.30-5.89)	(2.05-7.02)	(3.49-11.34)	(11.56-15.57)	(5.25-11.79)	(16.53-22.80)	（14.86-21.46）	（7.30-18.81）	（20.02-34.15）
**70-74 years**	3.29	3.45	5.99	11.32	8.48	17.57	14.61	11.92	23.56
	(2.45-4.35)	(1.85-6.05)	(3.31-9.79)	(9.88-12.80)	(5.84-11.58)	(15.64-19.24)	（12.33-17.15）	（7.68-17.63）	（18.95-29.03）
**75-79 years**	2.22	2.75	4.86	8.81	7.87	13.45	11.03	10.62	18.31
	(1.65-2.95)	(1.50-4.68)	(2.81-7.44)	(7.84-9.76)	(5.81-9.96)	(12.55-14.16)	（9.49-12.71）	（7.31-14.64）	（15.36-21.60）
**80-84 years**	1.50	2.17	3.81	6.91	6.94	9.94	8.41	9.11	13.76
	(1.11-2.01)	(1.21-3.56)	(2.33-5.45)	(6.27-7.51)	(5.53-8.17)	(9.57-10.22)	（7.37-9.52）	（6.73-11.72）	（11.91-15.67）
**85-89 years**	0.79	1.31	2.25	4.42	4.73	5.92	5.21	6.04	8.17
	(0.58-1.07)	(0.75-2.06)	(1.47-3.00)	(4.08-4.72)	(4.04-5.25)	(5.80-6.00)	（4.66-5.79）	（4.78-7.31）	（7.27-9.00）
**Total**	**47.02**	**27.85**	**43.36**	**94.46**	**49.84**	**102.49**	**141.49**	**77.69**	**145.86**
	**(33.22-66.01)**	**(14.42-51.64)**	**(23.30-75.71)**	**(79.89-110.79)**	**(34.82-69.00)**	**(87.12-119.32)**	**（113.11-176.80）**	**（49.24-120.64）**	**（110.41-195.03）**

**CHN Criteria**									
**20-24 years**	1.14	2.29	4.15	0.75	0.44	0.23	1.89	2.73	4.38
	(0.47-2.73)	(1.47-3.52)	(1.60-9.76)	(0.46-1.21)	(0.15-1.23)	(0.10-0.55)	（0.92-3.93）	（1.63-4.75）	（1.70-10.31）
**25-29 years**	1.67	3.37	6.12	1.34	0.81	0.51	3.01	4.18	6.63
	(0.76-3.61)	(2.25-4.97)	(2.46-13.59)	(0.84-2.11)	(0.29-2.21)	(0.22-1.17)	（1.60-5.71）	（2.54-7.19）	（2.69-14.76）
**30-34 years**	2.77	5.60	10.16	2.76	1.73	1.30	5.53	7.33	11.45
	(1.39-5.41)	(3.89-7.96)	(4.27-21.23)	(1.79-4.23)	(0.63-4.56)	(0.58-2.84)	（3.18-9.64）	（4.53-12.52）	（4.86-24.07）
**35-39 years**	2.91	5.89	10.62	3.48	2.24	2.01	6.39	8.14	12.62
	(1.62-5.15)	(4.25-8.07)	(4.67-20.82)	(2.31-5.18)	(0.84-5.71)	(0.94-4.18)	（3.92-10.33）	（5.09-13.78）	（5.61-25.00）
**40-44 years**	3.46	6.98	12.42	4.84	3.30	3.48	8.40	10.28	15.90
	(2.12-5.56)	(5.21-9.23)	(5.74-22.79)	(3.37-7.13)	(1.27-8.02)	(1.70-6.86)	（5.49-12.69）	（6.48-17.24）	（7.44-29.66）
**45-49 years**	5.12	10.23	17.86	8.64	6.00	7.37	13.76	16.24	25.23
	(3.45-7.49)	(7.89-13.10)	(8.70-30.66)	(6.05-12.06)	(2.38-13.71)	(3.77-13.51)	（9.50-19.55）	（10.27-26.81）	（12.47-44.17）
**50-54 years**	6.52	12.87	21.89	12.86	9.35	13.00	19.38	22.22	34.89
	(4.81-8.75)	(10.20-16.01)	(11.27-35.13)	(9.30-17.3)	(3.87-19.75)	(7.10-21.72)	（14.10-26.05）	（14.07-35.75）	（18.37-56.86）
**55-59 years**	6.81	13.20	21.73	15.17	11.58	17.49	21.99	24.78	39.22
	(5.44-8.47)	(10.72-16.01)	(11.87-32.68)	(11.35-19.61)	(5.07-22.25)	(10.39-26.18)	（16.79-28.08）	（15.25-34.29）	（22.26-58.86）
**60-64 years**	6.19	11.69	18.52	14.72	11.82	18.38	20.90	23.51	36.9
	(5.23-7.27)	(9.69-13.89)	(10.74-26.18)	(11.44-18.23)	(5.56-20.40)	(12.11-24.56)	（16.68-25.50）	（15.25-34.29）	（22.86-50.74）
**65-69 years**	7.36	13.47	20.46	18.98	16.03	24.33	26.34	29.51	44.79
	(6.36-8.47)	(11.35-15.74)	(12.62-27.32)	(15.37-22.53)	(8.25-24.75)	(17.91-29.42)	（21.73-31.00）	（19.60-40.49）	（30.53-56.74）
**70-74 years**	5.97	10.53	15.27	15.53	13.74	19.59	21.50	24.27	34.87
	(5.06-6.98)	(8.98-12.13)	(10.01-19.38)	(13.11-17.73)	(7.84-19.09)	(15.94-22.00)	（18.16-24.71）	（16.82-31.21）	（25.96-41.38）
**75-79 years**	4.37	7.39	10.23	11.65	10.71	14.13	16.02	18.10	24.36
	(3.55-5.28)	(6.37-8.40)	(7.11-12.41)	(10.21-12.85)	(6.82-13.57)	(12.42-15.11)	（13.76-18.13）	（13.18-21.98）	（19.52-27.52）
**80-84 years**	3.17	5.11	6.76	8.74	8.27	10.14	11.91	13.38	16.90
	(2.47-3.95)	(4.45-5.75)	(4.96-7.90)	(7.91-9.38)	(5.85-9.75)	(9.38-10.53)	（10.38-13.33）	（10.30-15.50）	（14.35-18.43）
**85-89 years**	1.77	2.72	3.44	5.34	5.16	5.96	7.11	7.88	9.40
	(1.32-2.26)	(2.39-3.02)	(2.66-3.90)	(4.97-5.61)	(4.00-5.77)	(5.69-6.08)	（6.29-7.87）	（6.39-8.79）	（8.35-9.98）
**Total**	**59.24**	**111.35**	**179.62**	**124.89**	**101.19**	**137.92**	**184.13**	**212.53**	**317.54**
	**(44.03-81.39)**	**(89.10-137.79)**	**(98.69-283.74)**	**(98.47-155.15)**	**(52.83-170.78)**	**(98.26-184.73)**	**（142.51-236.54）**	**（141.94-308.57）**	**（196.95-468.47）**

Notes: WHO: T-score of <-2.5 SD, CHN: T-score of <-2.0 SD.

Table S11. Estimated geographical regions- and sex-specific prevalence of osteoporosis in China in 2020

Geographical regions	Prevalence (%, 95%CI)								
	Male			Female			Total		
	Lumbar spine	Femoral neck	Ward	Lumbar spine	Femoral neck	Ward	Lumbar spine	Femoral neck	Ward
**WHO Criteria**									
**East**	8.60 (6.09-12.04)	5.23 (2.71-9.66)	8.19 (4.41-14.22)	18.34 (15.54-21.47)	9.88 (6.94-13.62)	20.16 (17.19-23.41)	13.41 (10.76-16.70)	7.53 (4.80-11.62)	14.11 (10.73-18.77)
**North**	8.37 (5.92-11.73)	4.99 (2.58-9.25)	7.77 (4.17-13.60)	17.64 (14.91-20.70)	9.23 (6.41-12.86)	19.28 (16.33-22.51)	12.97 (10.38-16.18)	7.09 (4.48-11.04)	13.48 (10.20-18.02)
**Northeast**	8.85 (6.31-12.31)	5.53 (2.87-10.21)	8.68 (4.66-15.16)	19.85 (16.81-23.23)	10.57 (7.32-14.74)	22.22 (18.80-25.95)	14.39 (11.59-17.81)	8.06 (5.11-12.49)	15.50 (11.78-20.59)
**Northwest**	8.34 (5.87-11.76)	4.71 (2.43-8.80)	7.27 (3.88-12.79)	15.80 (13.3-18.63)	7.88 (5.45-11.04)	16.49 (13.92-19.34)	12.01 (9.52-15.14)	6.27 (3.91-9.90)	11.80 (8.81-16.01)
**South Central**	8.54 (6.01-12.05)	4.84 (2.50-9.03)	7.47 (4.00-13.12)	16.86 (14.23-19.82)	8.70 (6.08-12.05)	17.83 (15.14-20.81)	12.62 (10.04-15.86)	6.74 (4.26-10.51)	12.56 (9.46-16.89)
**Southwest**	9.13 (6.45-12.83)	5.48 (2.84-10.14)	8.57 (4.62-14.88)	18.16 (15.38-21.27)	9.76 (6.84-13.45)	19.86 (16.97-23.02)	13.60 (10.87-17.00)	7.60 (4.82-11.77)	14.16 (10.73-18.90)
**China**	**8.63 (6.10-12.12)**	**5.11 (2.65-9.48)**	**7.96 (4.28-13.90)**	**17.75 (15.01-20.82)**	**9.37 (6.54-12.97)**	**19.26 (16.37-22.43)**	**13.14 (10.50-16.42)**	**7.21 (4.57-11.20)**	**13.54 (10.25-18.11)**
**CHN Criteria**									
**East**	11.00 (8.22-15.00)	20.61 (16.55-25.42)	33.12 (18.33-51.89)	24.27 (19.20-30.04)	19.75 (10.39-33.05)	26.94 (19.31-35.86)	17.56 (13.65-22.44)	20.19 (13.50-29.19)	30.06 (18.81-43.97)
**North**	10.61 (7.93-14.49)	19.95 (15.99-24.66)	32.18 (17.71-50.65)	23.40 (18.43-29.07)	18.94 (9.83-32.02)	26.00 (18.45-34.80)	16.95 (13.14-21.72)	19.45 (12.94-28.31)	29.12 (18.08-42.79)
**Northeast**	11.59 (8.80-15.51)	21.75 (17.54-26.68)	34.90 (19.43-53.94)	26.50 (20.94-32.78)	21.55 (11.20-36.14)	29.86 (21.22-39.76)	19.10 (14.91-24.20)	21.65 (14.35-31.44)	32.36 (20.33-46.80)
**Northwest**	10.23 (7.51-14.25)	19.31 (15.37-24.04)	31.38 (17.02-50.28)	20.82 (16.27-26.12)	16.68 (8.54-28.80)	22.64 (15.88-30.83)	15.83 (11.81-20.08)	18.02 (12.02-26.38)	27.09 (16.46-40.73)
**South Central**	10.48 (7.69-14.61)	19.77 (15.73-24.61)	32.08 (17.42-51.42)	22.19 (17.43-27.71)	17.89 (9.30-30.51)	24.22 (17.16-32.72)	16.23 (12.47-21.04)	18.85 (12.57-27.50)	28.22 (17.29-42.24)
**Southwest**	11.57 (8.59-15.89)	21.67 (17.36-26.80)	34.85 (19.23-54.93)	23.98 (18.96-29.71)	19.49 (10.26-32.66)	26.48 (19.02-35.26)	17.70 (13.72-22.73)	20.60 (13.85-29.70)	30.71 (19.13-45.20)
**China**	**10.87 (8.08-14.94)**	**20.44 (16.35-25.29)**	**32.97 (18.11-52.08)**	**23.47 (18.51-29.16)**	**19.02 (9.93-32.10)**	**25.92 (18.47-34.72)**	**17.10 (13.23-21.96)**	**19.74 (13.18-28.65)**	**29.49 (18.29-43.50)**

Notes: WHO: T-score of <-2.5 SD, CHN: T-score of <-2.0 SD.

Table S12. Estimated geographical regions- and sex-specific number of cases of osteoporosis in China in 2020

Geographical regions	Cases (million, 95%CI)								
	Male			Female			Total		
	Lumbar spine	Femoral neck	Ward	Lumbar spine	Femoral neck	Ward	Lumbar spine	Femoral neck	Ward
**WHO Criteria**									
**East**	14.27	8.69	13.60	29.80	16.06	32.76	44.07	24.74	46.36
	(10.11-19.99)	(4.51-16.04)	(7.33-23.62)	(25.25-34.88)	(11.27-22.13)	(27.93-38.04)	（35.36-55.87）	（15.78-38.17）	（35.26-61.66）
**North**	5.58	3.33	5.18	11.56	6.05	12.64	17.15	9.38	17.82
	(3.95-7.82)	(1.72-6.17)	(2.78-9.07)	(9.78-13.57)	(4.20-8.43)	(10.71-14.75)	（13.72-21.39）	（5.93-14.60）	（13.48-23.82）
**Northeast**	3.66	2.28	3.59	8.31	4.42	9.31	11.97	6.71	12.89
	(2.61-5.08)	(1.19-4.22)	(1.92-6.26)	(7.04-9.73)	(3.07-6.17)	(7.87-10.87)	（9.64-14.81）	（4.25-10.39）	（9.80-17.13）
**Northwest**	3.30	1.86	2.87	6.03	3.01	6.29	9.33	4.87	9.17
	(2.32-4.65)	(0.96-3.48)	(1.53-5.06)	(5.08-7.11)	(2.08-4.21)	(5.31-7.38)	（7.40-11.76）	（3.04-7.69）	（6.85-12.44）
**South Central**	13.13	7.44	11.48	24.98	12.89	26.42	38.11	20.34	37.90
	(9.23-18.52)	(3.84-13.88)	(6.15-20.17)	(21.08-29.36)	(9.01-17.86)	(22.43-30.82)	（30.32-47.89）	（12.85-31.73）	（28.57-50.99）
**Southwest**	7.08	4.25	6.64	13.78	7.40	15.07	20.86	11.66	21.71
	(5.00-9.95)	(2.20-7.86)	(3.58-11.53)	(11.67-16.14)	(5.19-10.20)	(12.87-17.46)	（16.67-26.08）	（7.39-18.06）	（16.45-28.99）
**China**	**47.02**	**27.85**	**43.36**	**94.46**	**49.84**	**102.49**	**141.49**	**77.69**	**145.86**
	**(33.22-66.01)**	**(14.42-51.64)**	**(23.30-75.71)**	**(78.89-110.79)**	**(34.82-69.00)**	**(87.12-119.32)**	**（113.11-176.80）**	**（49.24-120.64）**	**（110.41-195.03）**
**CHN Criteria**									
**East**	18.27	34.23	55.00	39.44	32.09	43.77	57.71	66.32	98.77
	(13.66-24.91)	(27.48-42.22)	(30.44-86.18)	(31.20-48.81)	(16.88-53.69)	(31.37-58.27)	（44.86-73.72）	（44.36-95.92）	（61.81-144.45）
**North**	7.07	13.30	21.45	15.34	12.42	17.04	22.41	25.72	38.5
	(5.28-9.66)	(10.66-16.44)	(11.81-33.76)	(12.08-19.06)	(6.45-20.99)	(12.1-22.81)	（17.37-28.72）	（17.10-37.43）	（23.90-56.58）
**Northeast**	4.79	8.98	14.41	11.10	9.02	12.50	15.89	18.00	26.92
	(3.63-6.41)	(7.24-11.02)	(8.03-22.28)	(8.77-13.73)	(4.69-15.13)	(8.88-16.65)	（12.40-20.13）	（11.93-26.15）	（16.91-38.93）
**Northwest**	4.04	7.64	12.41	7.95	6.37	8.64	11.99	14.00	21.05
	(2.97-5.63)	(6.08-9.51)	(6.73-19.88)	(6.21-9.97)	(3.26-10.99)	(6.06-11.77)	（9.18-15.60）	（9.34-20.50）	（12.79-31.65）
**South Central**	16.10	30.39	49.33	32.88	26.51	35.88	48.99	56.89	85.20
	(11.82-22.46)	(24.19-37.83)	(26.78-79.05)	(25.83-41.05)	(13.78-45.19)	(25.42-48.48)	（37.65-63.51）	（37.96-83.02）	（52.20-127.53）
**Southwest**	8.97	16.80	27.02	18.19	14.79	20.09	27.16	31.59	47.11
	(6.66-12.32)	(13.46-20.77)	(14.91-42.59)	(14.38-22.54)	(7.78-24.78)	(14.43-26.75)	（21.05-34.86）	（21.24-45.55）	（29.34-69.34）
**China**	**59.24**	**111.35**	**179.62**	**124.89**	**101.19**	**137.92**	**184.13**	**212.53**	**317.54**
	**(44.03-81.39)**	**(89.10-137.79)**	**(98.69-283.74)**	**(98.47-155.15)**	**(52.83-170.78)**	**(98.26-184.73)**	**（142.51-236.54）**	**（141.94-308.57）**	**（196.95-468.47）**

Notes: WHO: T-score of <-2.5 SD, CHN: T-score of <-2.0 SD.

Table S13. Estimated provinces- and sex-specific prevalence of osteoporosis in China in 2020

Province	Prevalence (%, 95%CI)								
	Male			Female			Total		
	Lumbar spine	Femoral neck	Ward	Lumbar spine	Femoral neck	Ward	Lumbar spine	Femoral neck	Ward
**WHO Criteria**									
**Beijing**	8.57 (6.02-12.09)	4.88 (2.52-9.08)	7.53 (4.05-13.19)	17.57 (14.85-20.62)	9.24 (6.47-12.77)	18.97 (16.12-22.08)	12.98 (10.35-16.27)	7.02 (4.46-10.89)	13.14 (9.97-17.55)
**Anhui**	8.69 (6.16-12.14)	5.47 (2.85-10.05)	8.62 (4.68-14.85)	18.52 (15.72-21.65)	10.17 (7.17-13.93)	20.47 (17.53-23.67)	13.60 (10.94-16.89)	7.82 (5.01-11.99)	14.54 (11.10-19.26)
**Fujian**	8.58 (6.04-12.08)	4.84 (2.49-9.03)	7.44 (3.98-13.11)	16.90 (14.24-19.90)	8.52 (5.94-11.86)	17.61 (14.88-20.64)	12.66 (10.06-15.92)	6.64 (4.18-10.42)	12.43 (9.32-16.80)
**Guangdong**	8.22 (5.72-11.74)	4.15 (2.12-7.85)	6.23 (3.30-11.13)	15.09 (12.66-17.86)	7.25 (5.05-10.11)	14.93 (12.58-17.58)	11.46 (8.99-14.63)	5.61 (3.50-8.92)	10.34 (7.68-14.17)
**Guizhou**	9.11 (6.42-12.82)	5.32 (2.75-9.87)	8.26 (4.44-14.42)	17.43 (14.74-20.44)	9.25 (6.48-12.76)	18.83 (16.06-21.86)	13.25 (10.56-16.62)	7.27 (4.60-11.31)	13.52 (10.22-18.12)
**Heilongjiang**	8.96 (6.38-12.47)	5.49 (2.85-10.17)	8.58 (4.59-15.06)	19.58 (16.56-22.95)	10.27 (7.10-14.36)	21.67 (18.30-25.36)	14.29 (11.49-17.73)	7.89 (4.98-12.27)	15.15 (11.47-20.23)
**Liaoning**	8.88 (6.34-12.34)	5.67 (2.95-10.44)	8.95 (4.82-15.55)	20.25 (17.18-23.66)	11.00 (7.66-15.28)	22.99 (19.51-26.75)	14.61 (11.80-18.05)	8.36 (5.32-12.88)	16.03 (12.23-21.20)
**Jiangxi**	8.79 (6.21-12.32)	5.21 (2.70-9.66)	8.11 (4.35-14.18)	17.98 (15.20-21.11)	9.39 (6.56-13.02)	19.34 (16.41-22.56)	13.35 (10.67-16.68)	7.29 (4.61-11.33)	13.68 (10.34-18.34)
**Jilin**	8.66 (6.16-12.05)	5.32 (2.76-9.86)	8.33 (4.46-14.61)	19.49 (16.48-22.85)	10.18 (7.03-14.27)	21.60 (18.22-25.29)	14.12 (11.37-17.49)	7.77 (4.91-12.08)	15.02 (11.40-20.00)
**Jiangsu**	8.86 (6.29-12.38)	5.57 (2.90-10.24)	8.78 (4.76-15.16)	19.27 (16.37-22.50)	10.68 (7.53-14.64)	21.59 (18.49-24.96)	14.05 (11.31-17.42)	8.12 (5.21-12.43)	15.17 (11.60-20.04)
**Hunan**	9.20 (6.54-12.82)	5.81 (3.03-10.69)	9.17 (4.96-15.84)	19.39 (16.45-22.67)	10.59 (7.44-14.58)	21.59 (18.43-25.03)	14.25 (11.46-17.70)	8.19 (5.22-12.62)	15.33 (11.64-20.40)
**Ningxia**	7.69 (5.40-10.88)	4.18 (2.15-7.85)	6.38 (3.39-11.32)	14.99 (12.58-17.72)	7.23 (4.97-10.17)	15.18 (12.77-17.89)	11.30 (8.95-14.26)	5.69 (3.54-8.99)	10.73 (8.02-14.56)
**Inner Mongolia**	8.24 (5.84-11.53)	4.86 (2.51-9.04)	7.53 (4.02-13.26)	17.11 (14.43-20.11)	8.68 (5.99-12.18)	18.36 (15.45-21.57)	12.60 (10.06-15.75)	6.74 (4.22-10.58)	12.85 (9.64-17.35)
**Qinghai**	8.25 (5.78-11.68)	4.35 (2.22-8.20)	6.57 (3.47-11.76)	14.93 (12.52-17.67)	7.10 (4.89-10.00)	14.89 (12.50-17.58)	11.52 (9.08-14.61)	5.70 (3.53-9.08)	10.64 (7.89-14.61)
**Shaanxi**	8.77 (6.20-12.3)	5.23 (2.71-9.70)	8.16 (4.38-14.25)	17.63 (14.90-20.68)	9.27 (6.45-12.88)	19.22 (16.31-22.39)	13.13 (10.49-16.43)	7.22 (4.55-11.26)	13.61 (10.26-18.26)
**Chongqing**	9.18 (6.53-12.8)	5.95 (3.10-10.89)	9.44 (5.13-16.21)	19.74 (16.77-23.03)	11.02 (7.75-15.11)	22.32 (19.13-25.76)	14.45 (11.64-17.90)	8.48 (5.42-13.00)	15.87 (12.12-20.98)
**Sichuan**	9.47	6.10	9.67	20.09	11.19	22.67	14.76	8.64	16.14

	(6.73-13.20)	(3.18-11.18)	(5.25-16.61)	(17.07-23.44)	(7.87-15.35)	(19.43-26.17)	(11.88-18.30)	(5.52-13.26)	(12.31-21.37)
**Zhejiang**	8.57 (6.05-12.05)	5.01 (2.59-9.29)	7.76 (4.17-13.57)	17.80 (15.04-20.90)	9.26 (6.48-12.84)	19.05 (16.16-22.24)	13.00 (10.37-16.30)	7.05 (4.46-10.99)	13.19 (9.93-17.73)
**Tianjin**	8.63 (6.09-12.10)	5.15 (2.67-9.54)	8.04 (4.32-14.04)	18.27 (15.46-21.40)	9.73 (6.77-13.53)	20.30 (17.23-23.63)	13.33 (10.66-16.64)	7.39 (4.67-11.49)	14.02 (10.62-18.71)
**Shanghai**	8.31 (5.86-11.69)	4.97 (2.58-9.19)	7.77 (4.19-13.49)	18.93 (16.06-22.12)	10.41 (7.34-14.29)	21.17 (18.09-24.48)	13.44 (10.79-16.72)	7.60 (4.88-11.65)	14.24 (10.90-18.79)
**Gansu**	8.95 (6.34-12.53)	5.35 (2.77-9.92)	8.34 (4.47-14.58)	17.51 (14.78-20.58)	9.04 (6.27-12.59)	18.78 (15.91-21.95)	13.21 (10.54-16.53)	7.18 (4.51-11.25)	13.53 (10.15-18.24)
**Shandong**	8.33 (5.92-11.62)	5.19 (2.7-9.57)	8.16 (4.40-14.18)	18.33 (15.56-21.43)	10.02 (7.02-13.81)	20.54 (17.51-23.83)	13.36 (10.76-16.55)	7.62 (4.87-11.70)	14.38 (10.99-19.03)
**Hebei**	8.12 (5.76-11.35)	4.95 (2.57-9.16)	7.75 (4.16-13.54)	17.85 (15.11-20.92)	9.45 (6.56-13.16)	19.77 (16.76-23.04)	13.02 (10.46-16.17)	7.21 (4.57-11.17)	13.80 (10.50-18.32)
**Tibet**	7.50 (5.18-10.77)	3.38 (1.7-6.53)	4.92 (2.55-9.05)	12.75 (10.63-15.20)	5.62 (3.84-7.99)	11.91 (9.93-14.16)	9.96 (7.74-12.85)	4.43 (2.71-7.22)	8.20 (6.01-11.45)
**Guangxi**	8.64 (6.10-12.12)	5.12 (2.65-9.49)	7.97 (4.29-13.89)	18.36 (15.57-21.48)	10.01 (7.07-13.70)	20.10 (17.20-23.26)	13.39 (10.73-16.69)	7.51 (4.81-11.55)	13.90 (10.60-18.47)
**Hainan**	8.58 (6.01-12.15)	4.70 (2.42-8.80)	7.19 (3.85-12.67)	16.51 (13.94-19.40)	8.57 (6.05-11.77)	17.23 (14.66-20.07)	12.35 (9.78-15.59)	6.54 (4.14-10.21)	11.96 (8.99-16.18)
**Shanxi**	8.72 (6.17-12.22)	5.16 (2.67-9.58)	8.03 (4.30-14.07)	17.37 (14.67-20.41)	8.97 (6.23-12.51)	18.72 (15.83-21.90)	12.97 (10.35-16.25)	7.04 (4.42-11.02)	13.29 (9.97-17.92)
**Henan**	8.72 (6.18-12.20)	5.35 (2.78-9.88)	8.40 (4.53-14.60)	17.86 (15.13-20.90)	9.62 (6.74-13.28)	19.71 (16.80-22.89)	13.40 (10.76-16.66)	7.54 (4.81-11.62)	14.20 (10.81-18.84)
**Xinjiang**	7.25 (5.02-10.39)	3.47 (1.76-6.62)	5.16 (2.71-9.29)	11.35 (9.41-13.59)	4.69 (3.15-6.78)	10.22 (8.45-12.24)	9.22 (7.13-11.94)	4.06 (2.43-6.70)	7.60 (5.48-10.71)
**Yunnan**	8.61 (5.97-12.31)	4.26 (2.17-8.08)	6.37 (3.36-11.42)	14.07 (11.78-16.69)	6.64 (4.59-9.31)	13.76 (11.61-16.17)	11.25 (8.78-14.43)	5.41 (3.34-8.68)	9.95 (7.35-13.72)
**Hubei**	8.19 (5.74-11.61)	4.35 (2.23-8.19)	6.60 (3.49-11.79)	14.96 (12.54-17.72)	7.11 (4.90-10.01)	14.91 (12.52-17.60)	11.55 (9.11-14.64)	5.72 (3.55-9.09)	10.72 (7.97-14.67)
**China**	8.63 (6.10-12.12)	5.11 (2.65-9.48)	7.96 (4.28-13.90)	17.75 (15.01-20.82)	9.37 (6.54-12.97)	19.26 (16.37-22.43)	13.14 (10.50-16.42)	7.21 (4.57-11.20)	13.54 (10.25-18.11)
**CHN Criteria**									
**Beijing**	10.52 (7.72-14.67)	19.81 (15.77-24.66)	32.12 (17.46-51.47)	23.16 (18.25-28.80)	18.75 (9.80-31.67)	25.50 (18.17-34.10)	16.72 (12.89-21.60)	19.29 (12.84-28.10)	28.87 (17.81-42.95)
**Anhui**	11.32 (8.50-15.34)	21.11 (17.01-25.94)	33.72 (18.85-52.35)	24.49 (19.43-30.23)	19.99 (10.60-33.22)	27.17 (19.60-36.04)	17.90 (13.96-22.78)	20.55 (13.81-29.58)	30.44 (19.22-44.20)
**Fujian**	10.51 (7.71-14.64)	19.86 (15.81-24.73)	32.31 (17.50-51.80)	22.26 (17.41-27.89)	17.86 (9.20-30.75)	24.19 (16.98-32.96)	16.27 (12.47-21.14)	18.88 (12.57-27.68)	28.33 (17.25-42.56)
**Guangdong**	9.40 (6.68-13.65)	17.93 (14.06-22.67)	29.57 (15.56-49.16)	19.68 (15.27-24.91)	15.63 (7.96-27.46)	20.87 (14.49-28.89)	14.26 (10.74-18.96)	16.84 (11.18-24.93)	25.46 (15.05-39.59)
**Guizhou**	11.36	21.37	34.53	22.98	18.63	25.25	17.14	20.01	29.91

	(8.39-15.72)	(17.06-26.50)	(18.90-54.86)	(18.13-28.55)	(9.76-31.40)	(18.06-33.79)	(13.24-22.11)	(13.43-28.94)	(18.48-44.38)
**Heilongjiang**	11.62<br>(8.78-15.60)	21.86<br>(17.59-26.86)	35.20<br>(19.49-54.69)	26.13<br>(20.60-32.41)	21.18<br>(10.95-35.77)	29.32<br>(20.73-39.28)	18.90<br>(14.71-24.04)	21.52<br>(14.26-31.33)	32.25<br>(20.11-46.96)
**Liaoning**	11.77<br>(8.97-15.67)	22.00<br>(17.78-26.93)	35.16<br>(19.71-54.01)	27.04<br>(21.44-33.33)	22.08<br>(11.56-36.70)	30.61<br>(21.90-40.46)	19.47<br>(15.26-24.58)	22.04<br>(14.65-31.86)	32.86<br>(20.81-47.18)
**Jiangxi**	11.10<br>(8.26-15.20)	20.87<br>(16.71-25.81)	33.69<br>(18.51-53.13)	23.78<br>(18.71-29.59)	19.22<br>(10.00-32.60)	26.18<br>(18.58-35.24)	17.39<br>(13.45-22.34)	20.05<br>(13.38-29.18)	29.96<br>(18.54-44.25)
**Jilin**	11.25<br>(8.52-15.09)	21.15<br>(17.02-25.98)	34.02<br>(18.86-52.80)	26.03<br>(20.50-32.29)	21.09<br>(10.88-35.63)	29.23<br>(20.65-39.15)	18.70<br>(14.56-23.76)	21.12<br>(13.93-30.85)	31.61<br>(19.76-45.92)
**Jiangsu**	11.55<br>(8.69-15.62)	21.55<br>(17.36-26.48)	34.43<br>(19.24-53.43)	25.52<br>(20.29-31.43)	20.89<br>(11.10-34.53)	28.52<br>(20.63-37.61)	18.51<br>(14.47-23.50)	21.22<br>(14.24-30.49)	31.48<br>(19.93-45.55)
**Hunan**	12.05<br>(9.10-16.23)	22.51<br>(18.15-27.62)	35.97<br>(20.12-55.64)	25.73<br>(20.40-31.75)	20.99<br>(11.09-34.92)	28.72<br>(20.65-38.09)	18.84<br>(14.70-23.93)	21.76<br>(14.65-31.24)	32.37<br>(20.38-46.93)
**Ningxia**	9.23<br>(6.71-13.00)	17.52<br>(13.88-21.91)	28.64<br>(15.36-46.42)	19.69<br>(15.30-24.86)	15.67<br>(7.94-27.44)	21.16<br>(14.69-29.18)	14.40<br>(10.96-18.86)	16.60<br>(10.94-24.64)	24.94<br>(15.03-37.91)
**Inner Mongolia**	10.42<br>(7.79-14.21)	19.67<br>(15.75-24.31)	31.85<br>(17.44-50.17)	22.73<br>(17.82-28.38)	18.29<br>(9.38-31.33)	25.16<br>(17.63-34.10)	16.47<br>(12.72-21.17)	18.99<br>(12.62-27.76)	28.45<br>(17.54-42.27)
**Qinghai**	9.75<br>(7.04-13.82)	18.59<br>(14.68-23.32)	30.57<br>(16.24-49.94)	19.60<br>(15.19-24.81)	15.55<br>(7.85-27.41)	20.94<br>(14.45-29.10)	14.57<br>(11.03-19.19)	17.10<br>(11.34-25.32)	25.86<br>(15.37-39.75)
**Shaanxi**	11.10<br>(8.28-15.20)	20.85<br>(16.70-25.77)	33.60<br>(18.50-52.91)	23.34<br>(18.40-29.00)	18.91<br>(9.84-31.92)	25,.86<br>(18.40-34.60)	17.13<br>(13.26-21.99)	19.90<br>(13.32-28.80)	29.78<br>(18.45-43.89)
**Chongqing**	12.18<br>(9.23-16.34)	22.65<br>(18.30-27.72)	35.99<br>(20.29-55.31)	26.19<br>(20.85-32.19)	21.47<br>(11.42-35.36)	29.36<br>(21.29-38.60)	19.17<br>(15.03-24.25)	22.06<br>(14.87-31.53)	32.68<br>(20.79-46.97)
**Sichuan**	12.52<br>(9.47-16.83)	23.30<br>(18.82-28.54)	37.08<br>(20.86-57.09)	26.64<br>(21.20-32.77)	21.83<br>(11.61-36.00)	29.84<br>(21.63-39.28)	19.55<br>(15.31-24.77)	22.57<br>(15.23-32.26)	33.47<br>(21.25-48.22)
**Zhejiang**	10.70<br>(7.91-14.78)	20.14<br>(16.09-24.97)	32.56<br>(17.81-51.70)	23.51<br>(18.49-29.28)	18.99<br>(9.88-32.25)	25.83<br>(18.31-34.81)	16.85<br>(13.00-21.75)	19.59<br>(13.11-28.47)	29.33<br>(18.05-43.58)
**Tianjin**	10.92<br>(8.16-14.95)	20.50<br>(16.43-25.34)	33.01<br>(18.20-51.98)	24.25<br>(19.16-30.02)	19.71<br>(10.29-33.03)	27.11<br>(19.37-35.98)	17.42<br>(13.52-22.30)	20.12<br>(13.43-29.09)	30.13<br>(18.77-44.18)
**Shanghai**	10.49<br>(7.81-14.43)	19.66<br>(15.74-24.32)	31.60<br>(17.43-49.88)	25.01<br>(19.86-30.83)	20.44<br>(10.86-33.84)	27.90<br>(20.18-36.69)	17.50<br>(13.63-22.35)	20.04<br>(13.38-28.91)	29.81<br>(18.76-43.51)
**Gansu**	11.37<br>(8.49-15.52)	21.39<br>(17.15-26.42)	34.51<br>(18.99-54.25)	23.19<br>(18.22-28.91)	18.71<br>(9.67-31.87)	25.54<br>(18.04-34.50)	17.25<br>(13.32-22.17)	20.06<br>(13.43-29.13)	30.05<br>(18.52-44.43)
**Shandong**	10.85<br>(8.18-14.62)	20.31<br>(16.35-24.95)	32.54<br>(18.12-50.49)	24.36<br>(19.32-30.04)	19.89<br>(10.49-33.05)	27.29<br>(19.62-36.12)	17.63<br>(13.78-22.37)	20.10<br>(13.41-29.02)	29.90<br>(18.87-43.27)
**Hebei**	10.44<br>(7.86-14.15)	19.60<br>(15.75-24.15)	31.51<br>(17.45-49.20)	23.73<br>(18.73-29.41)	19.26<br>(10.02-32.38)	26.51<br>(18.88-35.30)	17.12<br>(13.32-21.82)	19.43<br>(12.87-28.29)	28.99<br>(18.17-42.21)
**Tibet**	8.10<br>(5.60-12.09)	15.66<br>(12.13-20.05)	26.27<br>(13.40-44.92)	16.54<br>(12.67-21.23)	12.93<br>(6.40-23.44)	17.13<br>(11.63-24.28)	12.07<br>(8.92-16.38)	14.38<br>(9.44-21.64)	21.98<br>(12.57-35.23)
**Guangxi**	10.89<br>(8.07-14.97)	20.45<br>(16.37-25.31)	33.00<br>(18.13-52.16)	24.22<br>(19.19-29.95)	19.73<br>(10.46-32.91)	26.74<br>(19.27-35.54)	17.40<br>(13.51-22.29)	20.10<br>(13.48-29.02)	29.94<br>(18.68-44.04)

**Hainan**	10.30 (7.47-14.56)	19.49 (15.44-24.38)	31.79 (17.09-51.56)	21.63 (17.00-27.02)	17.44 (9.13-29.73)	23.42 (16.62-31.70)	15.68 (12.00-20.48)	18.51 (12.44-26.92)	27.81 (16.87-42.13)
**Shanxi**	11.01 (8.21-15.07)	20.72 (16.59-25.63)	33.46 (18.38-52.75)	23.01 (18.08-28.67)	18.57 (9.60-31.59)	25.42 (17.96-34.24)	16.91 (13.06-21.75)	19.67 (13.16-28.56)	29.51 (18.17-43.65)
**Henan**	11.22 (8.43-15.24)	21.02 (16.89-25.89)	33.71 (18.72-52.62)	23.65 (18.72-29.27)	19.25 (10.12-32.19)	26.30 (18.86-34.98)	17.59 (13.70-22.42)	20.11 (13.42-29.12)	29.92 (18.79-43.58)
**Xinjiang**	8.05 (5.63-11.89)	15.42 (12.01-19.63)	25.60 (13.28-43.23)	14.66 (11.13-18.98)	11.33 (5.48-20.98)	14.96 (10.01-21.48)	11.24 (8.28-15.31)	13.45 (8.86-20.28)	20.46 (11.70-32.73)
**Yunnan**	9.73 (6.87-14.22)	18.59 (14.54-23.57)	30.73 (16.08-51.40)	18.28 (14.15-23.22)	14.47 (7.33-25.57)	19.20 (13.35-26.62)	13.87 (10.39-18.58)	16.59 (11.05-24.54)	25.15 (14.76-39.40)
**Hubei**	9.70 (7.03-13.74)	18.46 (14.59-23.15)	30.29 (16.15-49.37)	19.60 (15.19-24.82)	15.54 (7.85-27.39)	20.90 (14.46-28.97)	14.61 (11.08-19.23)	17.02 (11.25-25.25)	25.63 (15.31-39.26)
**China**	10.87 (8.08-14.94)	20.44 (16.35-25.29)	32.97 (18.11-52.08)	23.47 (18.51-29.16)	19.02 (9.93-32.10)	25.92 (18.47-34.72)	17.10 (13.23-21.96)	19.74 (13.18-28.65)	29.49 (18.29-43.50)

Notes: WHO: T-score of <-2.5 SD, CHN: T-score of <-2.0 SD.

Table S14. Estimated provinces- and sex-specific number of cases of osteoporosis in China in 2020

Province	Cases (million, 95%CI)								
	Male			Female			Total		
	Lumbar spine	Femoral neck	Ward	Lumbar spine	Femoral neck	Ward	Lumbar spine	Femoral neck	Ward
**WHO Criteria**									
**Beijing**	0.81 (0.57-1.14)	0.46 (0.24-0.86)	0.71 (0.38-1.25)	1.60 (1.35-1.88)	0.84 (0.59-1.16)	1.73 (1.47-2.01)	2.41 (1.92-3.02)	1.3 (0.83-2.02)	2.44 (1.85-3.26)
**Anhui**	1.99 (1.41-2.78)	1.25 (0.65-2.3)	1.98 (1.07-3.4)	4.24 (3.60-4.96)	2.33 (1.64-3.19)	4.69 (4.01-5.42)	6.23 (5.01-7.74)	3.58 (2.29-5.49)	6.66 (5.09-8.82)
**Fujian**	1.37 (0.97-1.93)	0.77 (0.4-1.44)	1.19 (0.64-2.1)	2.60 (2.19-3.06)	1.31 (0.91-1.82)	2.71 (2.29-3.17)	3.97 (3.16-4.99)	2.08 (1.31-3.27)	3.9 (2.92-5.27)
**Guangdong**	4.15 (2.88-5.92)	2.09 (1.07-3.96)	3.14 (1.66-5.61)	6.81 (5.71-8.06)	3.27 (2.28-4.56)	6.74 (5.68-7.93)	10.96 (8.6-13.98)	5.36 (3.35-8.52)	9.88 (7.34-13.54)
**Guizhou**	1.22 (0.86-1.72)	0.71 (0.37-1.32)	1.11 (0.59-1.93)	2.31 (1.96-2.71)	1.23 (0.86-1.69)	2.50 (2.13-2.90)	3.54 (2.82-4.43)	1.94 (1.23-3.02)	3.61 (2.73-4.83)
**Heilongjiang**	1.21 (0.86-1.68)	0.74 (0.38-1.37)	1.16 (0.62-2.03)	2.66 (2.25-3.12)	1.39 (0.96-1.95)	2.94 (2.49-3.44)	3.87 (3.11-4.8)	2.14 (1.35-3.32)	4.1 (3.1-5.47)
**Liaoning**	1.58 (1.13-2.2)	1.01 (0.53-1.86)	1.6 (0.86-2.77)	3.68 (3.12-4.29)	2.00 (1.39-2.77)	4.17 (3.54-4.86)	5.26 (4.25-6.50)	3.01 (1.92-4.64)	5.77 (4.4-7.63)
**Jiangxi**	1.42 (1.00-1.99)	0.84 (0.44-1.56)	1.31 (0.7-2.29)	2.86 (2.42-3.36)	1.49 (1.04-2.07)	3.08 (2.61-3.59)	4.28 (3.42-5.34)	2.33 (1.48-3.63)	4.38 (3.31-5.88)
**Jilin**	0.86 (0.61-1.20)	0.53 (0.28-0.98)	0.83 (0.44-1.46)	1.98 (1.67-2.32)	1.03 (0.71-1.45)	2.19 (1.85-2.57)	2.84 (2.29-3.52)	1.56 (0.99-2.43)	3.02 (2.29-4.02)
**Jiangsu**	3.02 (2.14-4.22)	1.9 (0.99-3.49)	2.99 (1.62-5.17)	6.52 (5.54-7.61)	3.61 (2.55-4.95)	7.31 (6.26-8.45)	9.54 (7.68-11.83)	5.51 (3.54-8.45)	10.3 (7.88-13.61)
**Hunan**	2.29 (1.63-3.19)	1.45 (0.75-2.66)	2.28 (1.23-3.95)	4.75 (4.03-5.56)	2.60 (1.82-3.57)	5.29 (4.52-6.13)	7.04 (5.66-8.75)	4.04 (2.58-6.24)	7.57 (5.75-10.08)
**Ningxia**	0.21 (0.14-0.29)	0.11 (0.06-0.21)	0.17 (0.09-0.3)	0.39 (0.33-0.46)	0.19 (0.13-0.27)	0.40 (0.33-0.47)	0.6 (0.47-0.75)	0.3 (0.19-0.48)	0.57 (0.42-0.77)
**Inner Mongolia**	0.82 (0.58-1.15)	0.48 (0.25-0.9)	0.75 (0.4-1.32)	1.65 (1.39-1.94)	0.84 (0.58-1.17)	1.77 (1.49-2.08)	2.47 (1.97-3.09)	1.32 (0.83-2.08)	2.52 (1.89-3.4)
**Qinghai**	0.18 (0.13-0.26)	0.1 (0.05-0.18)	0.15 (0.08-0.26)	0.32 (0.26-0.37)	0.15 (0.10-0.21)	0.32 (0.26-0.37)	0.5 (0.39-0.63)	0.25 (0.15-0.39)	0.46 (0.34-0.63)
**Shaanxi**	1.37 (0.97-1.92)	0.82 (0.42-1.52)	1.28 (0.69-2.23)	2.68 (2.26-3.14)	1.41 (0.98-1.95)	2.92 (2.48-3.40)	4.05 (3.23-5.06)	2.22 (1.4-3.47)	4.19 (3.16-5.63)
**Chongqing**	1.15 (0.82-1.61)	0.75 (0.39-1.37)	1.18 (0.64-2.03)	2.47 (2.10-2.88)	1.38 (0.97-1.89)	2.79 (2.39-3.22)	3.62 (2.92-4.49)	2.13 (1.36-3.26)	3.98 (3.04-5.26)

**Sichuan**	3.12 (2.21-4.34)	2.01 (1.05-3.68)	3.18 (1.73-5.46)	6.55 (5.57-7.65)	3.65 (2.57-5.01)	7.40 (6.34-8.54)	9.67 (7.78-11.99)	5.66 (3.62-8.69)	10.58 (8.07-14.01)
**Zhejiang**	2.35 (1.66-3.31)	1.37 (0.71-2.55)	2.13 (1.15-3.73)	4.52 (3.82-5.31)	2.35 (1.64-3.26)	4.84 (4.10-5.64)	6.87 (5.48-8.61)	3.73 (2.36-5.81)	6.97 (5.25-9.37)
**Tianjin**	0.50 (0.35-0.70)	0.3 (0.15-0.55)	0.47 (0.25-0.81)	1.01 (0.85-1.18)	0.54 (0.37-0.75)	1.12 (0.95-1.31)	1.51 (1.21-1.88)	0.84 (0.53-1.3)	1.59 (1.2-2.12)
**Shanghai**	0.93 (0.65-1.3)	0.55 (0.29-1.03)	0.87 (0.47-1.50)	1.97 (1.67-2.30)	1.08 (0.76-1.49)	2.20 (1.88-2.55)	2.90 (2.33-3.61)	1.64 (1.05-2.51)	3.07 (2.35-4.05)
**Gansu**	0.85 (0.60-1.19)	0.51 (0.26-0.94)	0.79 (0.42-1.38)	1.64 (1.38-1.92)	0.84 (0.59-1.18)	1.76 (1.49-2.05)	2.48 (1.98-3.11)	1.35 (0.85-2.12)	2.54 (1.91-3.43)
**Shandong**	3.19 (2.27-4.45)	1.99 (1.03-3.67)	3.13 (1.69-5.43)	7.09 (6.02-8.29)	3.87 (2.71-5.34)	7.95 (6.77-9.22)	10.28 (8.28-12.74)	5.86 (3.75-9.01)	11.07 (8.46-14.65)
**Hebei**	2.23 (1.58-3.12)	1.36 (0.71-2.52)	2.13 (1.14-3.72)	4.97 (4.20-5.82)	2.63 (1.82-3.66)	5.50 (4.66-6.41)	7.20 (5.79-8.94)	3.99 (2.53-6.18)	7.63 (5.81-10.13)
**Tibet**	0.10 (0.07-0.14)	0.05 (0.02-0.09)	0.07 (0.03-0.12)	0.15 (0.13-0.18)	0.07 (0.05-0.09)	0.14 (0.12-0.17)	0.25 (0.19-0.32)	0.11 (0.07-0.18)	0.21 (0.15-0.29)
**Guangxi**	1.54 (1.09-2.16)	0.91 (0.47-1.69)	1.42 (0.77-2.48)	3.13 (2.66-3.66)	1.71 (1.21-2.34)	3.43 (2.93-3.97)	4.67 (3.74-5.83)	2.62 (1.68-4.03)	4.85 (3.7-6.45)
**Hainan**	0.34 (0.24-0.48)	0.18 (0.09-0.35)	0.28 (0.15-0.50)	0.59 (0.49-0.69)	0.30 (0.21-0.42)	0.61 (0.52-0.71)	0.92 (0.73-1.16)	0.49 (0.31-0.76)	0.89 (0.67-1.21)
**Shanxi**	1.21 (0.86-1.7)	0.72 (0.37-1.33)	1.12 (0.6-1.96)	2.34 (1.97-2.75)	1.21 (0.84-1.68)	2.52 (2.13-2.95)	3.55 (2.83-4.45)	1.93 (1.21-3.02)	3.64 (2.73-4.91)
**Henan**	2.97 (2.1-4.15)	1.82 (0.95-3.36)	2.86 (1.54-4.97)	6.38 (5.40-7.47)	3.44 (2.41-4.74)	7.04 (6.00-8.17)	9.34 (7.5-11.61)	5.26 (3.35-8.1)	9.90 (7.54-13.14)
**Xinjiang**	0.69 (0.48-0.99)	0.33 (0.17-0.63)	0.49 (0.26-0.89)	1.01 (0.84-1.21)	0.42 (0.28-0.60)	0.91 (0.75-1.09)	1.70 (1.32-2.20)	0.75 (0.45-1.24)	1.40 (1.01-1.98)
**Yunnan**	1.49 (1.04-2.13)	0.74 (0.38-1.4)	1.1 (0.58-1.98)	2.29 (1.92-2.71)	1.08 (0.75-1.51)	2.24 (1.89-2.63)	3.78 (2.95-4.85)	1.82 (1.12-2.92)	3.34 (2.47-4.61)
**Hubei**	1.85 (1.30-2.62)	0.98 (0.50-1.85)	1.49 (0.79-2.66)	3.32 (2.79-3.93)	1.58 (1.09-2.22)	3.31 (2.78-3.91)	5.17 (4.08-6.56)	2.56 (1.59-4.07)	4.80 (3.57-6.57)
**China**	47.02 (33.22-66.01)	27.85 (14.42-51.64)	43.36 (23.3-75.71)	94.46 (79.89-110.79)	49.84 (34.82-69.00)	102.49 (87.12-119.32)	141.49 (113.11-176.8)	77.69 (49.24-120.64)	145.86 (110.41-195.03)
**CHN Criteria**									
**Beijing**	1.00 (0.73-1.39)	1.88 (1.49-2.33)	3.04 (1.65-4.87)	2.11 (1.66-2.63)	1.71 (0.89-2.89)	2.32 (1.66-3.11)	3.11 (2.40-4.01)	3.59 (2.39-5.22)	5.37 (3.31-7.98)
**Anhui**	2.59 (1.95-3.52)	4.84 (3.90-5.95)	7.73 (4.32-12.00)	5.61 (4.45-6.92)	4.58 (2.43-7.61)	6.22 (4.49-8.25)	8.20 (6.40-10.44)	9.41 (6.32-13.55)	13.95 (8.81-20.25)
**Fujian**	1.68 (1.23-2.34)	3.18 (2.53-3.95)	5.17 (2.80-8.28)	3.42 (2.68-4.29)	2.75 (1.42-4.73)	3.72 (2.61-5.07)	5.10 (3.91-6.63)	5.92 (3.94-8.68)	8.89 (5.41-13.35)
**Guangdong**	4.74 (3.37-6.88)	9.04 (7.10-11.44)	14.92 (7.85-24.80)	8.88 (6.89-11.24)	7.05 (3.59-12.39)	9.42 (6.54-13.04)	13.62 (10.26-18.12)	16.10 (10.69-23.83)	24.33 (14.39-37.84)

**Guizhou**	1.52<br>(1.12-2.11)	2.86<br>(2.29-3.55)	4.63<br>(2.53-7.35)	3.05<br>(2.41-3.79)	2.47<br>(1.30-4.17)	3.35<br>(2.40-4.49)	4.57<br>(3.53-5.90)	5.34<br>(3.58-7.72)	7.98<br>(4.93-11.84)
**Heilongjiang**	1.57<br>(1.18-2.10)	2.95<br>(2.37-3.62)	4.75<br>(2.63-7.38)	3.55<br>(2.80-4.40)	2.88<br>(1.49-4.86)	3.98<br>(2.82-5.33)	5.12<br>(3.98-6.51)	5.82<br>(3.86-8.48)	8.73<br>(5.44-12.71)
**Liaoning**	2.10<br>(1.60-2.80)	3.93<br>(3.17-4.80)	6.27<br>(3.52-9.63)	4.91<br>(3.89-6.05)	4.01<br>(2.10-6.66)	5.56<br>(3.98-7.34)	7.01<br>(5.49-8.85)	7.93<br>(5.27-11.47)	11.83<br>(7.49-16.98)
**Jiangxi**	1.79<br>(1.33-2.45)	3.37<br>(2.70-4.17)	5.44<br>(2.99-8.58)	3.78<br>(2.98-4.70)	3.06<br>(1.59-5.18)	4.16<br>(2.95-5.60)	5.57<br>(4.31-7.16)	6.43<br>(4.29-9.35)	9.60<br>(5.94-14.18)
**Jilin**	1.12<br>(0.85-1.50)	2.11<br>(1.70-2.59)	3.39<br>(1.88-5.27)	2.64<br>(2.08-3.27)	2.14<br>(1.10-3.61)	2.96<br>(2.09-3.97)	3.76<br>(2.93-4.78)	4.25<br>(2.80-6.21)	6.36<br>(3.98-9.24)
**Jiangsu**	3.94<br>(2.96-5.33)	7.35<br>(5.92-9.03)	11.74<br>(6.56-18.22)	8.64<br>(6.87-10.64)	7.07<br>(3.76-11.69)	9.65<br>(6.98-12.73)	12.57<br>(9.83-15.96)	14.42<br>(9.68-20.71)	21.39<br>(13.54-30.94)
**Hunan**	3.00<br>(2.27-4.04)	5.61<br>(4.52-6.88)	8.96<br>(5.01-13.86)	6.30<br>(5.00-7.78)	5.14<br>(2.72-8.56)	7.04<br>(5.06-9.33)	9.31<br>(7.27-11.82)	10.75<br>(7.24-15.44)	16.00<br>(10.07-23.19)
**Ningxia**	0.25<br>(0.18-0.35)	0.47<br>(0.37-0.59)	0.77<br>(0.41-1.24)	0.51<br>(0.40-0.65)	0.41<br>(0.21-0.72)	0.55<br>(0.38-0.76)	0.76<br>(0.58-1.00)	0.88<br>(0.58-1.30)	1.32<br>(0.80-2.01)
**Inner Mongolia**	1.04<br>(0.78-1.42)	1.96<br>(1.57-2.42)	3.18<br>(1.74-5.00)	2.19<br>(1.72-2.74)	1.76<br>(0.90-3.02)	2.43<br>(1.70-3.29)	3.23<br>(2.49-4.15)	3.72<br>(2.47-5.44)	5.60<br>(3.44-8.29)
**Qinghai**	0.22<br>(0.16-0.31)	0.41<br>(0.32-0.52)	0.68<br>(0.36-1.10)	0.41<br>(0.32-0.52)	0.33<br>(0.17-0.58)	0.44<br>(0.31-0.62)	0.63<br>(0.48-0.83)	0.74<br>(0.49-1.10)	1.12<br>(0.67-1.72)
**Shaanxi**	1.74<br>(1.29-2.38)	3.26<br>(2.61-4.03)	5.25<br>(2.89-8.27)	3.54<br>(2.79-4.40)	2.87<br>(1.49-4.84)	3.92<br>(2.79-5.25)	5.28<br>(4.09-6.78)	6.13<br>(4.10-8.87)	9.18<br>(5.69-13.52)
**Chongqing**	1.53<br>(1.16-2.05)	2.84<br>(2.30-3.48)	4.52<br>(2.55-6.94)	3.28<br>(2.61-4.03)	2.69<br>(1.43-4.42)	3.67<br>(2.66-4.83)	4.80<br>(3.77-6.08)	5.53<br>(3.73-7.90)	8.19<br>(5.21-11.77)
**Sichuan**	4.12<br>(3.11-5.54)	7.67<br>(6.19-9.39)	12.20<br>(6.86-18.78)	8.70<br>(6.92-10.69)	7.12<br>(3.79-11.75)	9.74<br>(7.06-12.82)	12.81<br>(10.03-16.23)	14.79<br>(9.98-21.14)	21.94<br>(13.92-31.60)
**Zhejiang**	2.94<br>(2.17-4.06)	5.53<br>(4.42-6.86)	8.94<br>(4.89-14.20)	5.97<br>(4.69-7.43)	4.82<br>(2.51-8.19)	6.56<br>(4.65-8.83)	8.91<br>(6.87-11.49)	10.35<br>(6.93-15.04)	15.50<br>(9.54-23.03)
**Tianjin**	0.63<br>(0.47-0.87)	1.19<br>(0.95-1.47)	1.92<br>(1.06-3.02)	1.34<br>(1.06-1.66)	1.09<br>(0.57-1.82)	1.50<br>(1.07-1.99)	1.97<br>(1.53-2.53)	2.28<br>(1.52-3.30)	3.41<br>(2.13-5.00)
**Shanghai**	1.17<br>(0.87-1.61)	2.19<br>(1.76-2.71)	3.53<br>(1.95-5.57)	2.60<br>(2.07-3.21)	2.13<br>(1.13-3.52)	2.91<br>(2.10-3.82)	3.78<br>(2.94-4.82)	4.32<br>(2.89-6.24)	6.43<br>(4.05-9.39)
**Gansu**	1.08<br>(0.80-1.47)	2.02<br>(1.62-2.50)	3.27<br>(1.80-5.13)	2.17<br>(1.70-2.70)	1.75<br>(0.90-2.98)	2.39<br>(1.69-3.22)	3.24<br>(2.51-4.17)	3.77<br>(2.53-5.48)	5.65<br>(3.48-8.36)
**Shandong**	4.16<br>(3.14-5.60)	7.78<br>(6.27-9.56)	12.46<br>(6.94-19.34)	9.42<br>(7.47-11.62)	7.69<br>(4.06-12.78)	10.56<br>(7.59-13.97)	13.58<br>(10.61-17.22)	15.47<br>(10.32-22.34)	23.02<br>(14.53-33.31)
**Hebei**	2.87<br>(2.16-3.89)	5.39<br>(4.33-6.64)	8.66<br>(4.80-13.53)	6.60<br>(5.21-8.18)	5.36<br>(2.79-9.01)	7.37<br>(5.25-9.82)	9.47<br>(7.37-12.07)	10.75<br>(7.12-15.64)	16.03<br>(10.05-23.35)
**Tibet**	0.11<br>(0.07-0.16)	0.21<br>(0.16-0.27)	0.35<br>(0.18-0.60)	0.20<br>(0.15-0.25)	0.15<br>(0.08-0.28)	0.20<br>(0.14-0.29)	0.30<br>(0.22-0.41)	0.36<br>(0.24-0.54)	0.55<br>(0.32-0.89)

**Guangxi**	1.94 (1.44-2.67)	3.65 (2.92-4.52)	5.89 (3.24-9.31)	4.13 (3.27-5.11)	3.36 (1.78-5.61)	4.56 (3.29-6.06)	6.07 (4.71-7.78)	7.02 (4.71-10.13)	10.45 (6.52-15.37)
**Hainan**	0.40 (0.29-0.57)	0.76 (0.61-0.96)	1.25 (0.67-2.02)	0.77 (0.60-0.96)	0.62 (0.32-1.05)	0.83 (0.59-1.12)	1.17 (0.90-1.53)	1.38 (0.93-2.01)	2.08 (1.26-3.15)
**Shanxi**	1.53 (1.14-2.10)	2.89 (2.31-3.57)	4.66 (2.56-7.35)	3.10 (2.43-3.86)	2.50 (1.29-4.25)	3.42 (2.42-4.61)	4.63 (3.58-5.96)	5.39 (3.60-7.82)	8.08 (4.98-11.96)
**Henan**	3.82 (2.87-5.18)	7.15 (5.75-8.81)	11.47 (6.37-17.90)	8.45 (6.68-10.45)	6.87 (3.61-11.50)	9.39 (6.73-12.49)	12.26 (9.55-15.64)	14.02 (9.36-20.30)	20.86 (13.10-30.39)
**Xinjiang**	0.77 (0.54-1.14)	1.47 (1.15-1.88)	2.45 (1.27-4.13)	1.31 (0.99-1.69)	1.01 (0.49-1.87)	1.33 (0.89-1.91)	2.08 (1.53-2.83)	2.48 (1.64-3.75)	3.78 (2.16-6.05)
**Yunnan**	1.69 (1.19-2.47)	3.22 (2.52-4.09)	5.33 (2.79-8.91)	2.97 (2.30-3.78)	2.35 (1.19-4.16)	3.12 (2.17-4.33)	4.66 (3.49-6.24)	5.57 (3.71-8.24)	8.45 (4.96-13.24)
**Hubei**	2.19 (1.59-3.10)	4.17 (3.30-5.23)	6.84 (3.65-11.15)	4.35 (3.37-5.51)	3.45 (1.74-6.08)	4.64 (3.21-6.43)	6.54 (4.96-8.62)	7.62 (5.04-11.31)	11.48 (6.86-17.59)
**China**	59.24 (44.03-81.39)	111.35 (89.10-137.79)	179.62 (98.69-283.74)	124.89 (98.47-155.15)	101.19 (52.83-170.78)	137.92 (98.26-184.73)	184.13 (142.51-236.54)	212.53 (141.94-308.57)	317.54 (196.95-468.47)

Notes: WHO: T-score of <-2.5 SD, CHN: T-score of <-2.0 SD.

Figure S1. Meta-analyses of associated factors for osteoporosis

Associated factor	ID	Author	Year Published	Random-effects meta-analysis
**Factor 1-Female sex (vs male sex)**				
	55	FK Zhang,et al.	2014	
	57	XW Gong,et al.	2014	
	58	XL Huang,et al.	2014	
	61	DD Pang	2015	
	65	LF Zhao,et al.	2016	
	69	L Lin,et al.	2016	
	78	LB Yao,et al.	2017	
	83	ZL Ye,et al.	2017	
	90	RR Li,et al.	2018	
	92	Z Ma,et al.	2018	
	106	JL Li	2020	
	107	X Li,et al.	2020	
	108	Y Liu,et al.	2020	
	109	D Qiao	2020	
	110	NH Sang,et al.	2020	
	114	XW Zhu,et al.	2020	
	116	ZY Cao,et al.	2021	
	117	XM Chen,et al.	2021	
	118	LR Hong	2021	
	119	WJ Qiao	2021	
	120	J Shen,et al.	2021	
	124	XX Yang,et al.	2021	
	125	W Zhang,et al.	2021	
	126	HJ Zhou,et al.	2021	
	128	XX Yang,et al.	2021	
	51	GZ Xu, et al.	2013	
	60	J Tian,et al.	2014	
	100	YY Luo,et al.	2019	
	70	B Liu	2017	
**Factor 2-Age (per year)**				
	44	FT Wu,et al.	2011	
	54	CY Lee,et al.	2016	
	49	LY Ruan,et al.	2013	

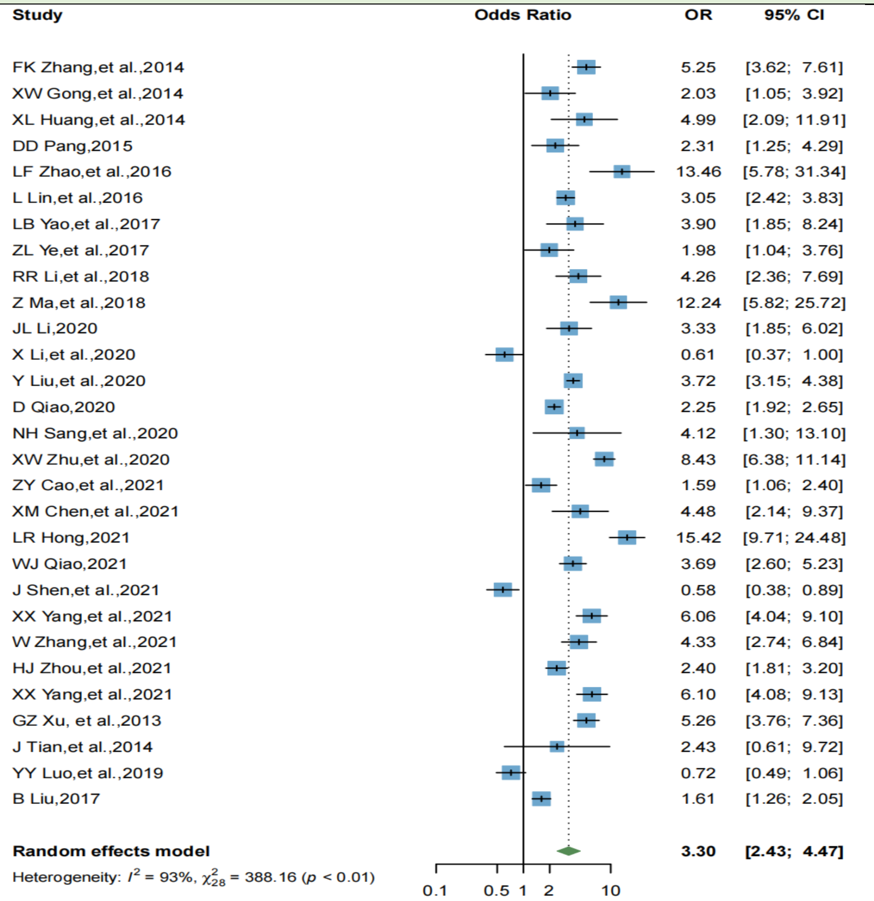

Associated factor	ID	Author	Year Published	Random-effects meta-analysis
	52	ZZ Zhang	2013	
	60	J Tian,et al.	2014	
	61	DD Pang	2015	
	65	LF Zhao,et al.	2016	
	69	L Lin,et al.	2016	
	75	CZ Bing,et al.	2017	
	76	G Cao,et al.	2017	
	77	XX Tan,et al.	2017	
	80	SJ Tan,et al.	2017	
	82	YJ lin,et al.	2017	
	85	Ko CH,et al.	2017	
	92	Z Ma,et al.	2018	
	94	XZ Wang,et al.	2018	
	104	QP Gu,et al.	2020	
	106	JL Li	2020	
	107	X Li,et al.	2020	
	108	Y Liu,et al.	2020	
	109	D Qiao	2020	
	114	XW Zhu,et al.	2020	
	120	J Shen,et al.	2021	
	121	XM Sun,et al.	2021	
	122	MJ Xia,et al.	2021	
	123	XL Xing,et al.	2021	
	56	LQ Kou	2014	
	111	B Wu,et al.	2020	
	115	X Zong,et al.	2020	
	71	WX Qi	2017	
	72	RP Yang	2017	
	79	J Zhang,et al.	2017	
	81	HL Zhang,et al.	2017	
	93	LM Tian,et al.	2018	
	46	CX Wang,et al.	2012	
	88	T Chen,et al.	2018	
**Factor 3-BMI**				
**per unit**	46	CX Wang,et al.	2012	

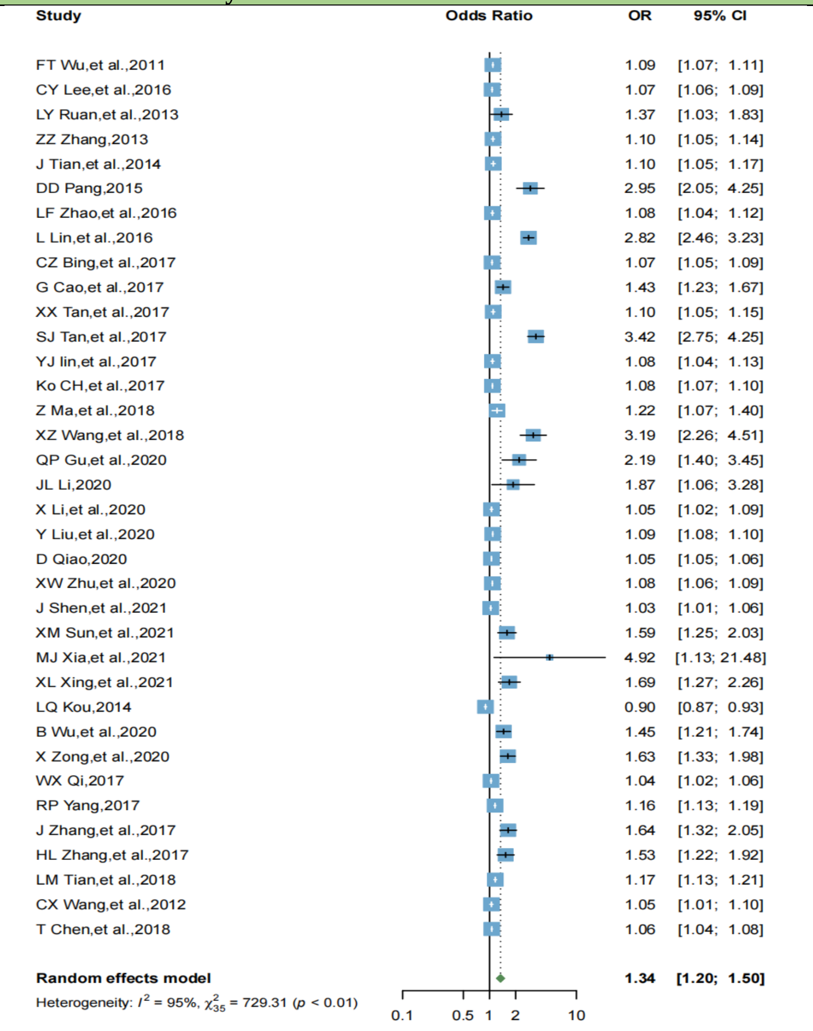

Associated factor	ID	Author	Year Published	Random-effects meta-analysis
	50	LY Wu,et al.	2013	
	52	ZZ Zhang	2013	
	65	LF Zhao,et al.	2016	
	60	J Tian,et al.	2014	
	61	DD Pang	2015	
	118	LR Hong	2021	
	122	MJ Xia,et al.	2021	
	77	XX Tan,et al.	2017	
	80	SJ Tan,et al.	2017	
	87	J Chen,et al.	2018	
	90	RR Li,et al.	2018	
	103	J Zeng,et al.	2020	
	82	YJ lin,et al.	2017	
	106	JL Li	2020	
	108	Y Liu,et al.	2020	
	56	LQ Kou	2014	
	104	QP Gu,et al.	2020	
	112	LB Yao,et al.	2020	
	71	WX Qi	2017	
	81	HL Zhang,et al.	2017	
	111	B Wu,et al.	2020	

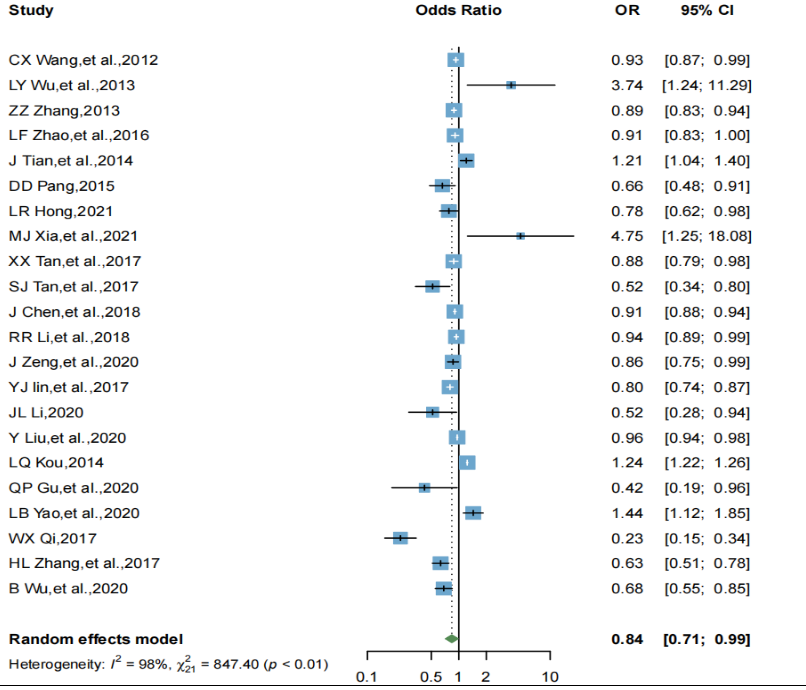

Associated factor	ID	Author	Year Published	Random-effects meta-analysis
**Under-weight (vs normal)**	59	QY Luo,et al.	2014	
	109	D Qiao	2020	
	119	WJ Qiao	2021	
	117	XM Chen,et al.	2021	
	100	YY Luo,et al.	2019	
	123	XL Xing,et al.	2021	
	121	XM Sun,et al.	2021	

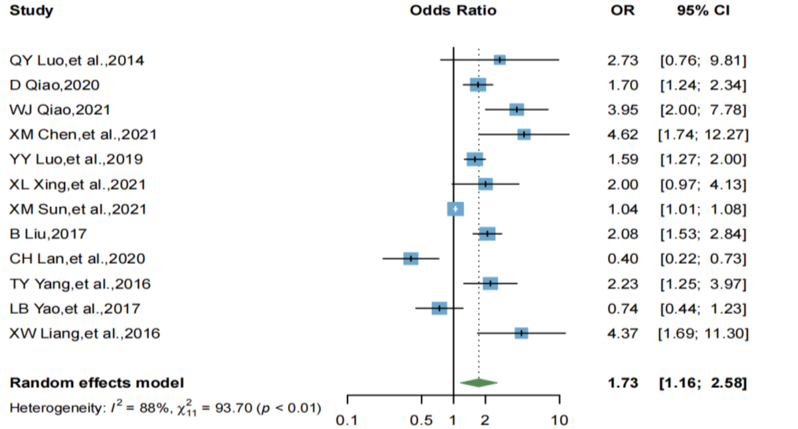

Associated factor	ID	Author	Year Published	Random-effects meta-analysis
	70	B Liu	2017	
	105	CH Lan,et al.	2020	
	66	TY Yang,et al.	2016	
	78	LB Yao,et al.	2017	
	64	XW Liang,et al.	2016	
**Over-weight (vs normal)**	59	QY Luo,et al.	2014	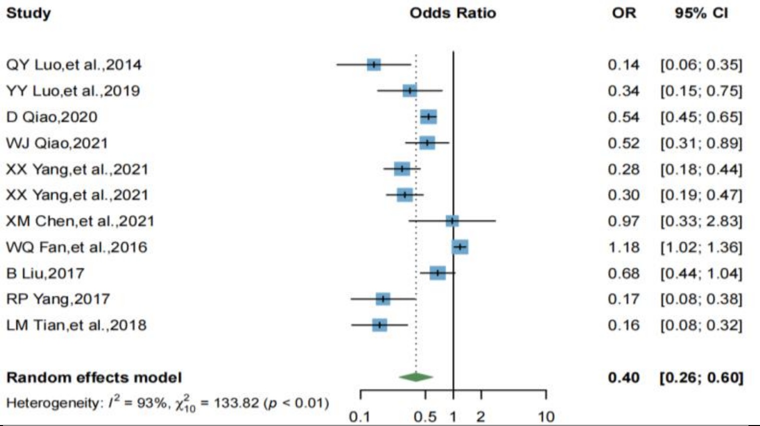
	100	YY Luo,et al.	2019	
	109	D Qiao	2020	
	119	WJ Qiao	2021	
	124	XX Yang,et al.	2021	
	128	XX Yang,et al.	2021	
	117	XM Chen,et al.	2021	
	67	WQ Fan,et al.	2016	
	70	B Liu	2017	
	72	RP Yang	2017	
	93	LM Tian,et al.	2018	
**Obesity (vs normal)**	59	QY Luo,et al.	2014	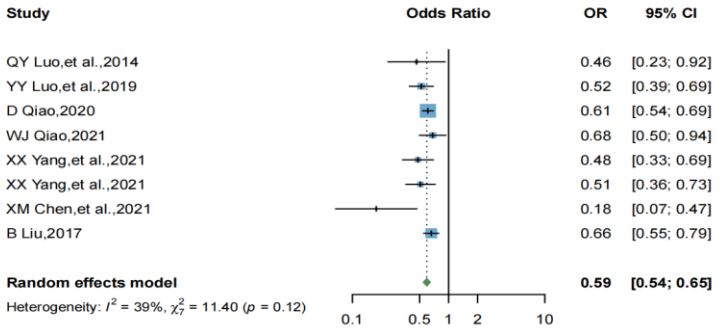
	100	YY Luo,et al.	2019	
	109	D Qiao	2020	
	119	WJ Qiao	2021	
	124	XX Yang,et al.	2021	
	128	XX Yang,et al.	2021	
	117	XM Chen,et al.	2021	
	70	B Liu	2017	
**Factor 4-Height (per unit)**				
	54	CY Lee,et al.	2016	
	60	J Tian,et al.	2014	

Associated factor	ID	Author	Year Published	Random-effects meta-analysis
	85	Ko CH,et al.	2017	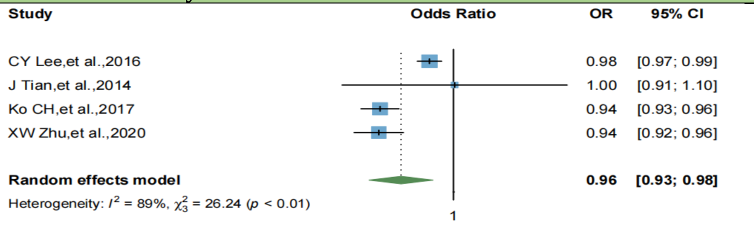
	114	XW Zhu,et al.	2020	

Factor 5-WHR (per unit)				
	46	CX Wang,et al.	2012	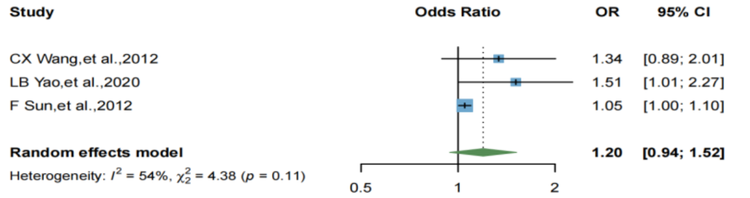
	112	LB Yao,et al.	2020	
	45	F Sun,et al.	2012	

Factor 6-Education				
**High school and above (vs primary and below)**	109	D Qiao	2020	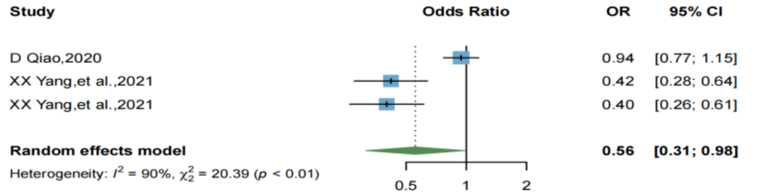
	124	XX Yang,et al.	2021	
	128	XX Yang,et al.	2021	

**Junior high school (vs primary and below)**	109	D Qiao	2020	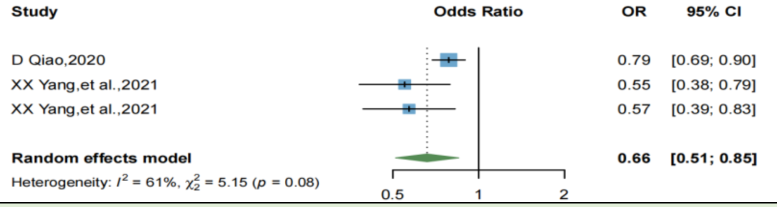
	124	XX Yang,et al.	2021	
	128	XX Yang,et al.	2021	

Factor 7- Mental work (vs manual labor)				
	60	J Tian,et al.	2014	

Associated factor	ID	Author	Year Published	Random-effects meta-analysis
	95	JJ Xia, et al.	2018	
	103	J Zeng,et al.	2020	

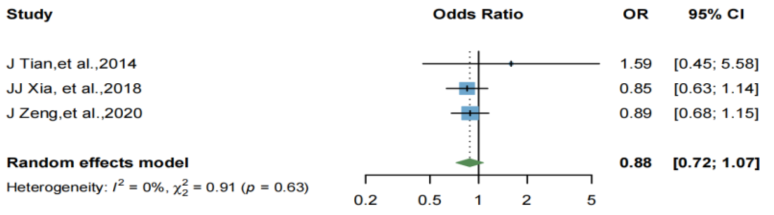

Factor 8- Mateless (vs married/cohabiting)	ID	Author	Year Published	Random-effects meta-analysis
	81	HL Zhang,et al.	2017	
	109	D Qiao	2020	
	127	XL Ren,et al.	2021	
	63	XL Li	2016	

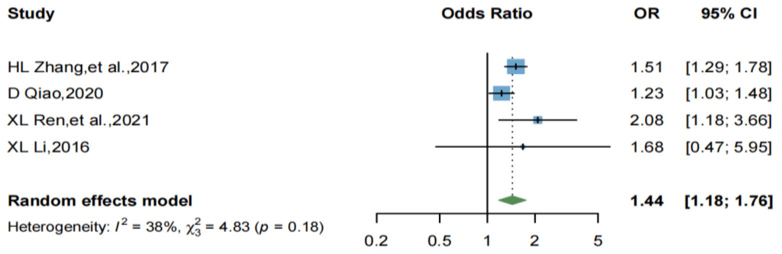

Factor 9-Current alcohol consumption (vs no current alcohol consumption)	ID	Author	Year Published	Random-effects meta-analysis
	2	J Tang,et al.	1997	
	48	R Li,et al.	2013	
	92	Z Ma,et al.	2018	
	67	WQ Fan,et al.	2016	
	60	J Tian,et al.	2014	
	76	G Cao,et al.	2017	
	107	X Li,et al.	2020	
	108	Y Liu,et al.	2020	
	109	D Qiao	2020	
	112	LB Yao,et al.	2020	
	111	B Wu,et al.	2020	
	106	JL Li	2020	
	90	RR Li,et al.	2018	
	50	LY Wu,et al.	2013	
	54	CY Lee,et al.	2016	
	61	DD Pang	2015	
	66	TY Yang,et al.	2016	

Associated factor	ID	Author	Year Published	Random-effects meta-analysis
	85	Ko CH,et al.	2017	
	73	YQ Zhao	2017	
	83	ZL Ye,et al.	2017	
	72	RP Yang	2017	
	63	XL Li	2016	
	70	B Liu	2017	

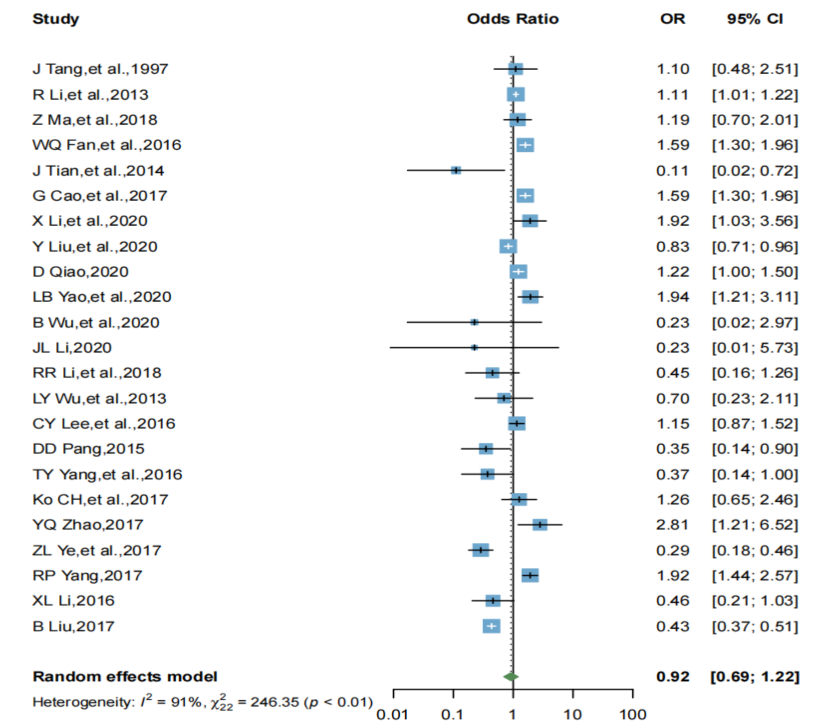

Factor 10-Current smoking (vs no current smoking)			
	54	CY Lee,et al.	2016
	85	Ko CH,et al.	2017
	2	J Tang,et al.	1997
	48	R Li,et al.	2013
	50	LY Wu,et al.	2013
	60	J Tian,et al.	2014
	120	J Shen,et al.	2021
	73	YQ Zhao	2017
	90	RR Li,et al.	2018
	97	QJ Zhu,et al.	2018

Associated factor	ID	Author	Year Published	Random-effects meta-analysis
	100	YY Luo,et al.	2019	
	106	JL Li	2020	
	107	X Li,et al.	2020	
	111	B Wu,et al.	2020	
	115	X Zong,et al.	2020	
	119	WJ Qiao	2021	
	63	XL Li	2016	
	70	B Liu	2017	
	72	RP Yang	2017	
	93	LM Tian,et al.	2018	

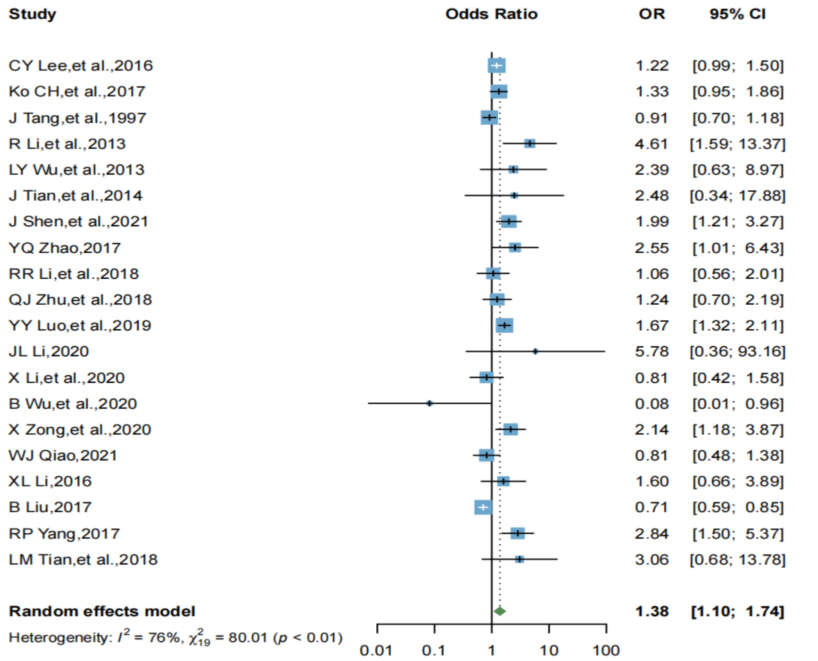

Factor 11-Exercise (vs no exercise)				
	114	XW Zhu,et al.	2020	
	53	Lo SS	2015	
	94	XZ Wang,et al.	2018	
	103	J Zeng,et al.	2020	
	50	LY Wu,et al.	2013	
	57	XW Gong,et al.	2014	
	79	J Zhang,et al.	2017	
	126	HJ Zhou,et al.	2021	
	106	JL Li	2020	
	18	M Li	1015	
	76	G Cao,et al.	2017	
	111	B Wu,et al.	2020	

Associated factor	ID	Author	Year Published	Random-effects meta-analysis
	73	YQ Zhao	2017	
	95	JJ Xia, et al.	2018	
	101	DJ Wang，et al	2019	
	2	J Tang,et al.	1997	
	93	LM Tian,et al.	2018	
	115	X Zong,et al.	2020	

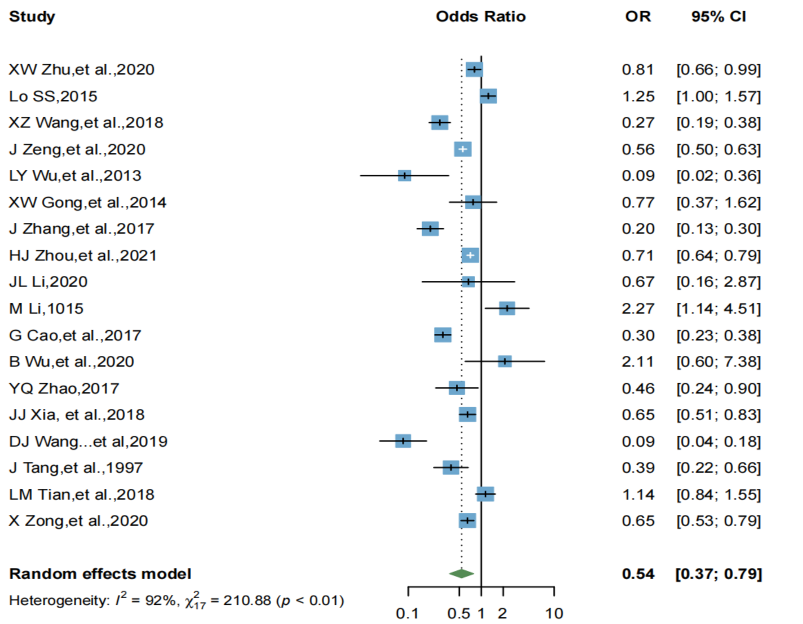

Factor 12-Milk drinking (vs no milk drinking)				
	64	XW Liang,et al.	2016	
	73	YQ Zhao	2017	
	76	G Cao,et al.	2017	
	78	LB Yao,et al.	2017	
	80	SJ Tan,et al.	2017	
	101	DJ Wang，et al	2019	
	106	JL Li	2020	
	124	XX Yang,et al.	2021	
	126	HJ Zhou,et al.	2021	
	128	XX Yang,et al.	2021	
	37	WF Sun,et al.	2009	
	48	R Li,et al.	2013	

Associated factor	ID	Author	Year Published	Random-effects meta-analysis
	50	LY Wu,et al.	2013	
	54	CY Lee,et al.	2016	
	56	LQ Kou	2014	
	57	XW Gong,et al.	2014	
	58	XL Huang,et al.	2014	
	62	NN Kou	2016	
	119	WJ Qiao	2021	
	63	XL Li	2016	
	67	WQ Fan,et al.	2016	
	81	HL Zhang,et al.	2017	
	111	B Wu,et al.	2020	
	112	LB Yao,et al.	2020	
	115	X Zong,et al.	2020	

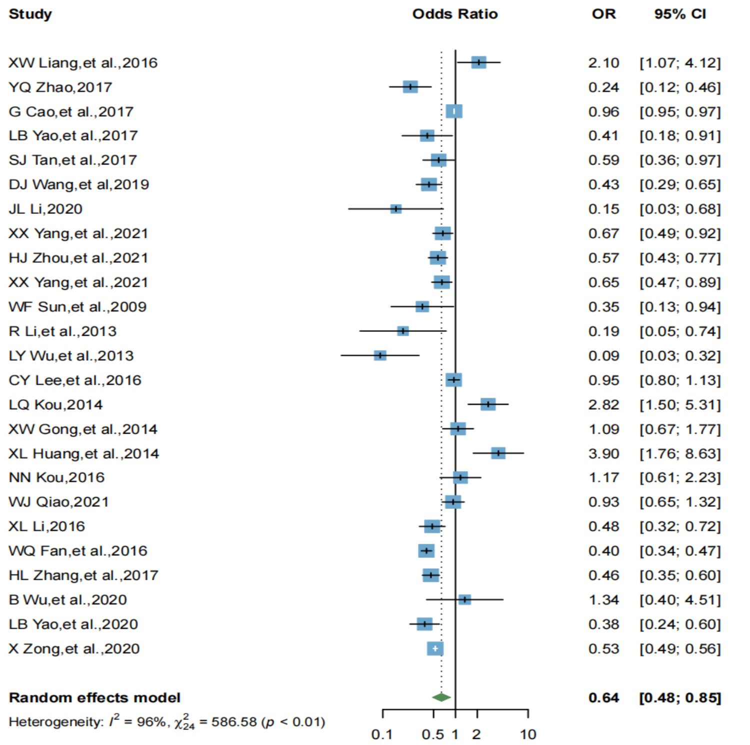

Factor 13-Tea drinking (vs no tea drinking)				
	50	LY Wu,et al.	2013	
	48	R Li,et al.	2013	
	66	TY Yang,et al.	2016	
	76	G Cao,et al.	2017	
	106	JL Li	2020	
	107	X Li,et al.	2020	
	111	B Wu,et al.	2020	
	63	XL Li	2016	

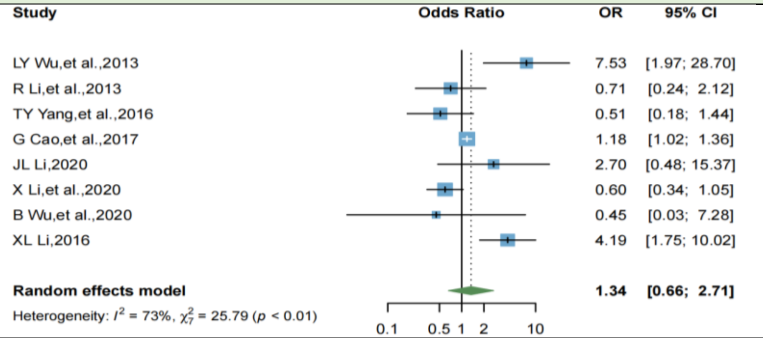

Associated factor	ID	Author	Year Published	Random-effects meta-analysis
**Factor 14-Take calcium (vs no take calcium)**				
	80	SJ Tan,et al.	2017	
	94	XZ Wang,et al.	2018	
	117	XM Chen,et al.	2021	
	28	PD Zhang,et al.	2008	
	76	G Cao,et al.	2017	
	103	J Zeng,et al.	2020	
	104	QP Gu,et al.	2020	
	72	RP Yang	2017	

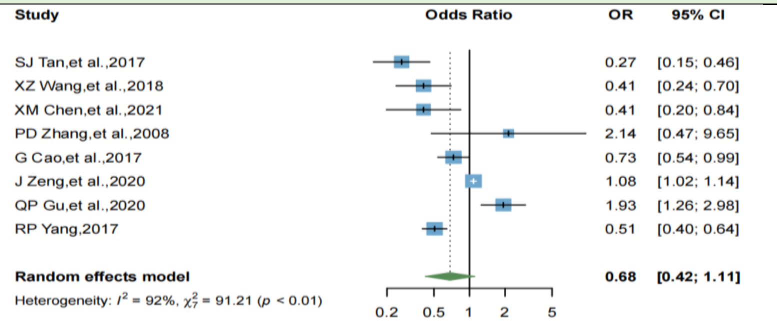

Associated factor	ID	Author	Year Published	Random-effects meta-analysis
**Factor 15-Vitamin D supplement (vs no vitamin D supplement)**				
	94	XZ Wang,et al.	2018	
	50	LY Wu,et al.	2013	
	123	XL Xing,et al.	2021	
	113	YH Zhang,et al.	2020	

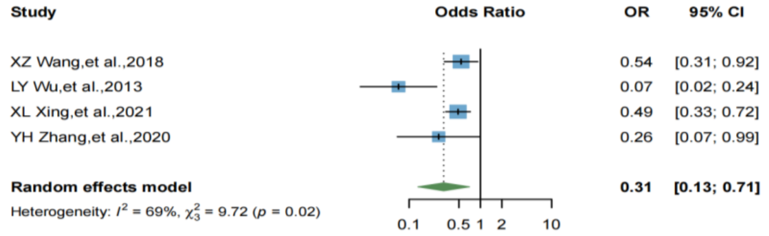

Associated factor	ID	Author	Year Published	Random-effects meta-analysis
**Factor 16-DBP (per unit)**				
	14	XW Zhu,et al.	2020	
	90	RR Li,et al.	2018	
	54	CY Lee,et al.	2016	

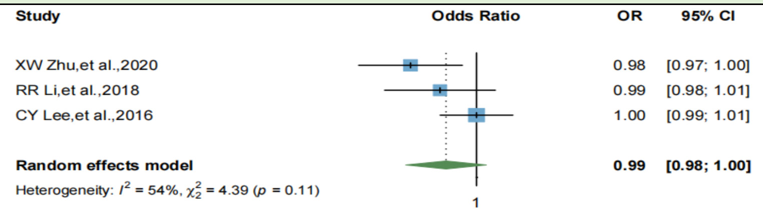

Associated factor	ID	Author	Year Published	Random-effects meta-analysis
**Factor 17-Hypertension (vs no hypertension)**				
	116	ZY Cao,et al.	2021	
	118	LR Hong	2021	
	119	WJ Qiao	2021	
	121	XM Sun,et al.	2021	
	126	HJ Zhou,et al.	2021	

Associated factor	ID	Author	Year Published	Random-effects meta-analysis
	57	XW Gong,et al.	2014	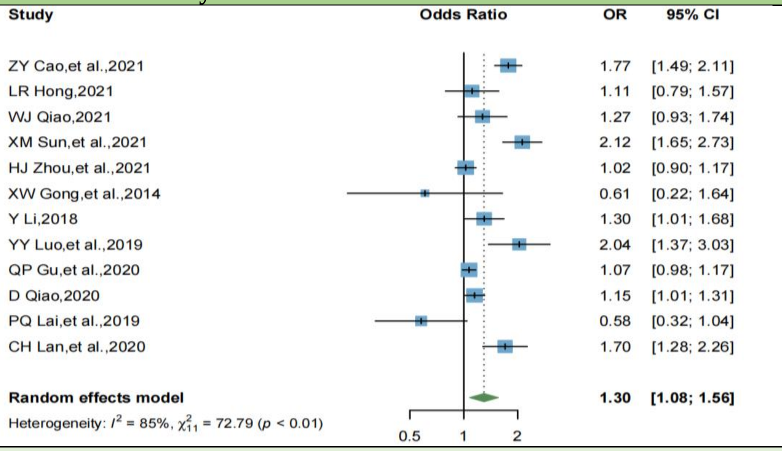
	91	Y Li	2018	
	100	YY Luo,et al.	2019	
	104	QP Gu,et al.	2020	
	109	D Qiao	2020	
	99	PQ Lai,et al.	2019	
	105	CH Lan,et al.	2020	
**Factor 18-Diabetes (vs no diabetes)**				
	100	YY Luo,et al.	2019	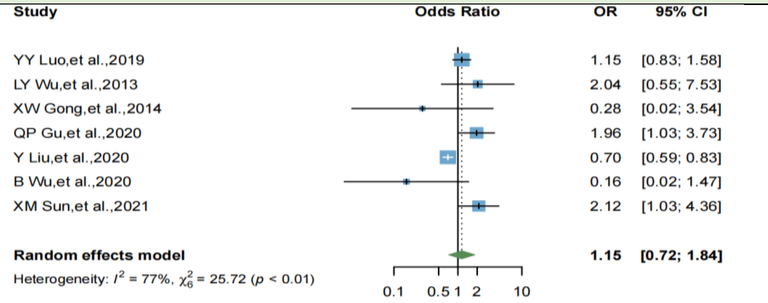
	50	LY Wu,et al.	2013	
	57	XW Gong,et al.	2014	
	104	QP Gu,et al.	2020	
	108	Y Liu,et al.	2020	
	111	B Wu,et al.	2020	
	121	XM Sun,et al.	2021	
**Factor 19-HDL-C (per unit)**				
	54	CY Lee,et al.	2016	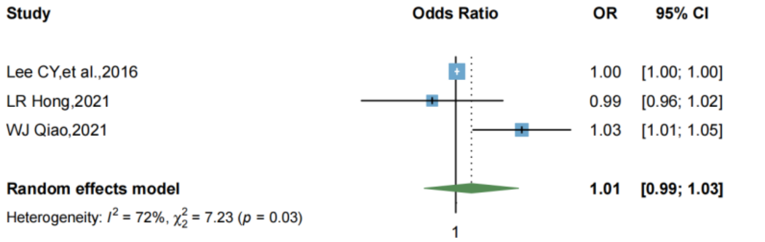
	118	LR Hong	2021	
	119	WJ Qiao	2021	
**Factor 20-Fracture history (vs no fracture history)**				

Associated factor	ID	Author	Year Published	Random-effects meta-analysis
	56	LQ Kou	2014	
	59	QY Luo,et al.	2014	
	50	LY Wu,et al.	2013	
	70	B Liu	2017	
	72	RP Yang	2017	
	73	YQ Zhao	2017	
	85	Ko CH,et al.	2017	
	92	Z Ma,et al.	2018	
	124	XX Yang,et al.	2021	
	127	XL Ren,et al.	2021	
	128	XX Yang,et al.	2021	

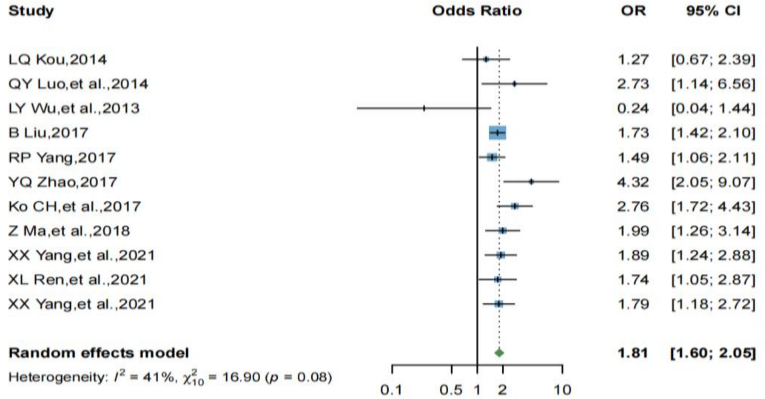

Factor 21-Age of menopause (per year)				
	56	LQ Kou	2014	
	46	CX Wang,et al.	2012	
	49	LY Ruan,et al.	2013	
	72	RP Yang	2017	
	93	LM Tian,et al.	2018	
	67	WQ Fan,et al.	2016	

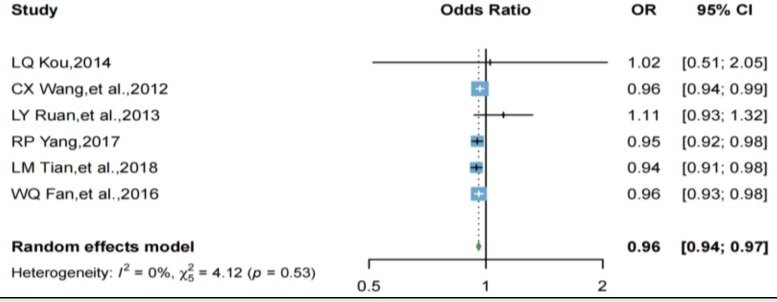

Factor 22-Menopause years (per year)				
	106	JL Li	2020	
	111	B Wu,et al.	2020	
	42	F Tian	2011	
	80	SJ Tan,et al.	2017	
	123	XL Xing,et al.	2021	
	80	SJ Tan,et al.	2017	
	123	XL Xing,et al.	2021	

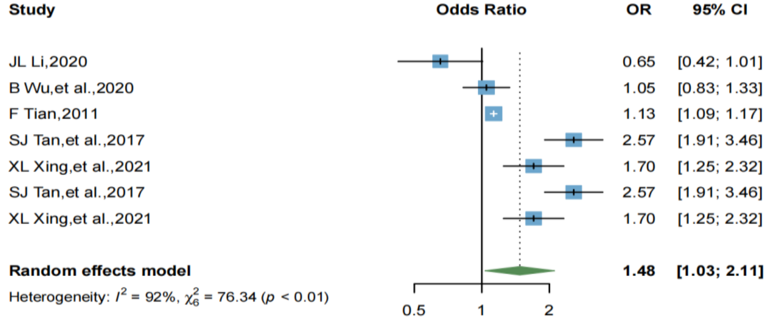

Associated factor	ID	Author	Year Published	Random-effects meta-analysis
**Factor 23-Menopause (vs no menopause)**				
	59	QY Luo,et al.	2014	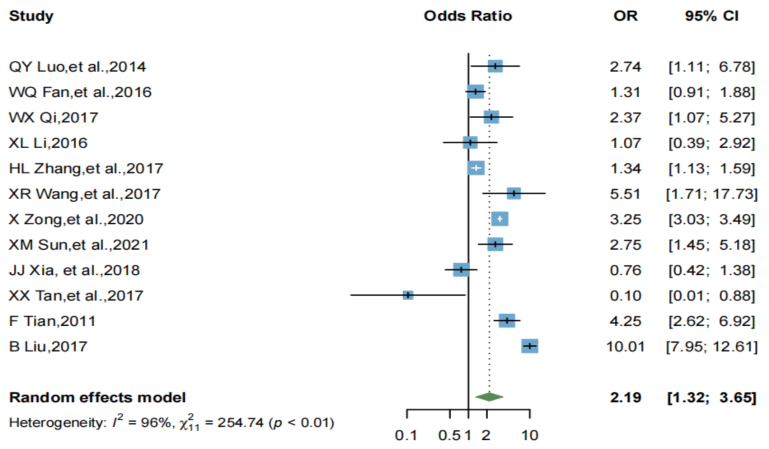
	67	WQ Fan,et al.	2016	
	71	WX Qi	2017	
	63	XL Li	2016	
	81	HL Zhang,et al.	2017	
	84	XR Wang,et al.	2017	
	115	X Zong,et al.	2020	
	121	XM Sun,et al.	2021	
	95	JJ Xia, et al.	2018	
	77	XX Tan,et al.	2017	
	42	F Tian	2011	
	70	B Liu	2017	

Associated factor	ID	Author	Year Published	Random-effects meta-analysis
**Factor 24-Parity times (per pregnancy)**				
	46	CX Wang,et al.	2012	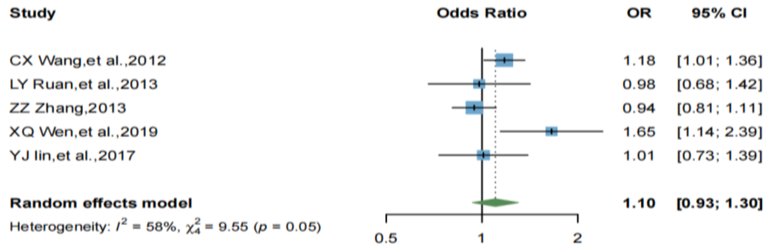
	49	LY Ruan,et al.	2013	
	52	ZZ Zhang	2013	
	102	XQ Wen,et al.	2019	
	82	YJ lin,et al.	2017	

Notes: BMI: Body mass index, WHR: Waist hip rate, DBP: Diastolic blood pressure, HDL-C: High-density lipoprotein cholesterol.

eMothed. Detailed process of the estimation of the prevalence of osteoporosis

Epidemiological modeling of the osteoporosis prevalence

To reduce heterogeneity, we only included studies that defined osteoporosis using the diagnostic criteria of WHO (T-score ⩽ -2.5 SD) or CHN (T-score ⩽ -2 SD, or 25% reduce of BMD), and categorized accordingly. In addition, the included studies assessed osteoporosis at various skeletal sites. However, we only chose those that assessed the following specific sites: lumbar spine, femoral neck, or ward's triangle.

Given that:

$$\text{Prevalence of osteoporosis} = \mathrm{p} = \frac{\text{number of cases}}{\text{number of participants}}$$

Then, the prevalence was stabilised with the logit link,

$$\mathrm{logit(p)} = \ln\left(\frac{p}{1-p}\right) = ln(odds)$$

Given the well-acknowledged high discrepancy in prevalence between males and females, the prevalence estimation models were constructed separately by sex. First, a univariable meta-regression was used to explore the association of osteoporosis prevalence and each cluster-level factor, including age, publication year, investigation year, study setting, and latitude. The results are shown in **Table S3** in the Online Supplementary Document. Considering the number of data points included in the model, Age was the only factor that was included in each multivariable model. To control the effects of multiple data points from the same study, a random effect (u_i) was added into the model to control the effects of multiple data points from the same study.

$$\mathrm{logit(p)} = \mathrm{\alpha} + \beta \times Age + u_i$$

Then,

$$\text{Prevalence of osteoporosis} = \mathrm{p} = \frac{e^{(\mathrm{\alpha}+\beta\times Age+u_i)}}{1+e^{(\mathrm{\alpha}+\beta\times Age+u_i)}}$$

Where α is the intercept term, β is the coefficient, u_i= variance of the study-level random effect.

Finally, age-specific prevalence of osteoporosis was generated by diagnostic criteria, skeletal site for males and females respectively.

Estimation of the national prevalence and cases of osteoporosis in 2020

The number of Chinese adults with osteoporosis by diagnostic criteria, skeletal site in males and females was calculated by multiplying the age-specific osteoporosis prevalence estimates with the corresponding population data obtained from the 7th National Census of Mainland China[1]. This was done for every 5-year age group, ranging from 20-89 years. Then, the overall prevalence and cases of osteoporosis among Chinese adults in the year 2020 were derived.

Meta-analysis of factors associated with osteoporosis

A random-effects meta-analysis was used to pool the ORs for major associated factors of osteoporosis. As a rule, we only included factors that had been investigated in at least three individual studies using a multivariate logistic regression. To ensure consistency among the associated factors across all included studies, we converted them to a unified unit based on the most commonly used unit in the included studies. Heterogeneity between studies was assessed using Cochran's Q statistic ($P<0{\cdot}05$ indicates significant heterogeneity) and the I^2 statistic ($\geqslant$50% indicates substantial heterogeneity)[2]. Two researchers (XH and KT) assessed the evidence credibility of associated factors and divided them into 5 categories: class I (convincing evidence), class II (highly suggestive evidence), class III (suggestive evidence), class IV (weak evidence), and NS (non-significant) (**Table S3**).

Estimation of the regional prevalence and cases of osteoporosis in 2020

We also estimated the regional prevalence and cases of osteoporosis in 2020 using an "associated factor-based model". To examine the distribution of osteoporosis in different regions, China was classified into six geographic regions (**Table S9**). Four major associated factors (overweight, obesity, hypertension and current smoking) were found to have a significant association with the prevalence of osteoporosis in the previous step and have subnational prevalence data[3-5]. Therefore, they were included in the "associated factor-based model". Using the same "associated factor-based" approach, we also

estimated the prevalence and cases of osteoporosis among 31 provinces in mainland China in 2020. The formula as follows:

$$N_{Province} = Pop_{province} \times Prev_{national} \times [1 + (Prev_{f_1\_province} - Prev_{f_1\_national}) \times (OR_{f_1} - 1) + (Prev_{f_2\_province} - Prev_{f_2\_national}) \times (OR_{f_2} - 1) + (Prev_{f_3\_province} - Prev_{f_3\_national}) \times (OR_{f_3} - 1) + (Prev_{f_4\_province} - Prev_{f_4\_national}) \times (OR_{f_4} - 1)]$$

Where $N_{Province}$and $Pop_{province}$ represent the number of osteoporosis cases and population size of adults in each province. $Prev_{national}$ indicates the estimated national prevalence of osteoporosis that were generated in the fist stage. $Prev_{f\_province}$ and $Prev_{f\_national}$ are the prevalence rates of each associated factor in each province and in China. OR_f is the estimated OR of each associated factor in China. f_1-f_4 were overweight, obesity, hypertension and current smoking.

Regional cases were generated by summing up the number of osteoporosis cases of all provinces within the region. Finally, the regional and provincial prevalence of osteoporosis were generated by dividing the number of osteoporosis cases by the corresponding paediatric population in 2020.

Reference

1. National Bureau of Statistics. *China Population Census Yearbook 2020*. (China Statistics Press, Beijing, 2022).

2.Higgins JP. *et al.* Measuring inconsistency in meta-analyses. *BMJ*. **557–60,** 327 (2003).

3. Chen, K. *et al.* Prevalence of obesity and associated complications in China: A cross-sectional, real-world study in 15.8 million adults. *Diabetes Obes. Metab.* **25**, 3390–3399 (2023).

4. Wang, Z. *et al.* Status of Hypertension in China: Results From the China Hypertension Survey, 2012-2015. *Circulation* **137**, 2344–2356 (2018).

5. Zhang, M. *et al.* Trends in smoking prevalence in urban and rural China, 2007 to 2018: Findings from 5 consecutive nationally representative cross-sectional surveys. *PLoS Med.* **19**, e1004064 (2022).
